# The necroptosis-inducing kinase RIPK3 dampens adipose tissue inflammation and glucose intolerance

**DOI:** 10.1038/ncomms11869

**Published:** 2016-06-21

**Authors:** Jérémie Gautheron, Mihael Vucur, Anne T. Schneider, Ilenia Severi, Christoph Roderburg, Sanchari Roy, Matthias Bartneck, Peter Schrammen, Mauricio Berriel Diaz, Josef Ehling, Felix Gremse, Felix Heymann, Christiane Koppe, Twan Lammers, Fabian Kiessling, Niels Van Best, Oliver Pabst, Gilles Courtois, Andreas Linkermann, Stefan Krautwald, Ulf P. Neumann, Frank Tacke, Christian Trautwein, Douglas R. Green, Thomas Longerich, Norbert Frey, Mark Luedde, Matthias Bluher, Stephan Herzig, Mathias Heikenwalder, Tom Luedde

**Affiliations:** 1 Department of Medicine III, University Hospital RWTH Aachen, Aachen 52074, Germany; 2 Division of GI and Hepatobiliary Oncology, University Hospital RWTH Aachen, Aachen 52074, Germany; 3 Department of Experimental and Clinical Medicine, University of Ancona, Ancona 60020, Italy; 4 Institute for Diabetes and Cancer IDC Helmholtz Center Munich, Neuherberg 85764 and Joint Heidelberg-IDC Translational Diabetes Program, Inner Medicine I, Heidelberg University, Heidelberg 69120, Germany; 5 Department for Experimental Molecular Imaging, University Clinic and Helmholtz Institute for Biomedical Engineering RWTH Aachen, Aachen 52074, Germany; 6 Institut of Medical Microbiology, University Hospital RWTH Aachen, Aachen 52074, Germany; 7 Inserm U1038, BIG, CEA, Grenoble 38054, France; 8 Division of Nephrology and Hypertension, Christian-Albrechts-University, Kiel 24105, Germany; 9 Department of Visceral and Transplantation Surgery, University Hospital RWTH Aachen, Aachen 52074, Germany; 10 Department of Immunology, St Jude Children's Research Hospital, Memphis, Tennessee 38105, USA; 11 Institute of Pathology, University Hospital RWTH Aachen, Aachen 52074, Germany; 12 Department of Cardiology and Angiology, University Hospital Schleswig-Holstein, Campus Kiel, Kiel 24105, Germany; 13 Department of Medicine, University of Leipzig, Leipzig 04103, Germany; 14 German Center for Diabetes Research (DZD), Neuherberg 85764, Germany; 15 Division of Chronic Inflammation and Cancer, German Cancer Research Center (DKFZ), Heidelberg 69120, Germany

## Abstract

Receptor-interacting protein kinase 3 (RIPK3) mediates necroptosis, a form of programmed cell death that promotes inflammation in various pathological conditions, suggesting that it might be a privileged pharmacological target. However, its function in glucose homeostasis and obesity has been unknown. Here we show that RIPK3 is over expressed in the white adipose tissue (WAT) of obese mice fed with a choline-deficient high-fat diet. Genetic inactivation of *Ripk3* promotes increased Caspase-8-dependent adipocyte apoptosis and WAT inflammation, associated with impaired insulin signalling in WAT as the basis for glucose intolerance. Similarly to mice, in visceral WAT of obese humans, RIPK3 is overexpressed and correlates with the body mass index and metabolic serum markers. Together, these findings provide evidence that RIPK3 in WAT maintains tissue homeostasis and suppresses inflammation and adipocyte apoptosis, suggesting that systemic targeting of necroptosis might be associated with the risk of promoting insulin resistance in obese patients.

## Abstract (web summary)

The kinase RIPK3 initiates necroptosis, which has been reported to promote inflammation in various pathological conditions. Here, the authors show that genetic ablation of *Ripk3* results in adipocyte apoptosis and white adipose tissue inflammation in obese mice, which promotes glucose intolerance.

## Graphical abstract

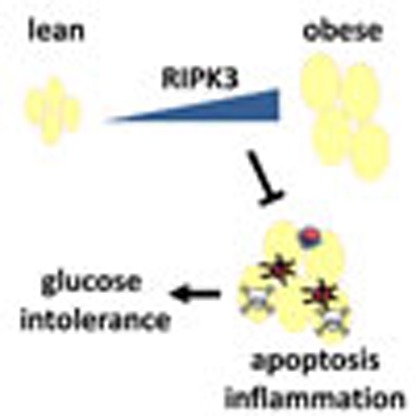

Diabetes mellitus is increasing at an alarming rate and represents a major global health burden affecting 8.3% of the adult population worldwide^1^. Type 2 diabetes mellitus has been associated with the pandemic spreading of overweight and obesity owing to the transition in lifestyle and dietary habits as well as ageing of the population in the setting of a genetical predisposition^2^. Excess adipose tissue predisposes towards the development of insulin resistance by an increased secretion of various adipocyte-derived proteins (referred as adipokines)^3^,^4^. Thereby, adipose tissue acts on many systemic processes including energy metabolism, inflammation and the complications of the metabolic syndrome. In mouse models of obesity, high-fat diet (HFD) feeding results in an adipose tissue inflammatory response characterized by the invasion of pro-inflammatory macrophages (also referred to as M1), accompanied by the secretion of a variety of inflammatory cytokines into the circulation^5^. This inflammatory cascade is believed to impair insulin signal transduction, thereby causing glucose intolerance^6^. In parallel, obesity triggers the organization of macrophages around dead and/or dying adipocytes in so-called adipocyte crown-like structures (CLSs)^7^. On the basis of morphologic criteria, it was shown that adipocytes within the CLS structures display both characteristics of necrosis^8^ and apoptosis^9^. However, up to now the exact contribution of distinct forms of regulated cell death within white adipose tissue (WAT) to glucose intolerance is not well understood.

Historically, cell death was divided into two forms: the programmed cell death apoptosis, widely considered to prevent inflammation, and the unregulated and accidental cell death—necrosis—which was considered to induce inflammation^10^. Apoptosis represents a highly synchronized intracellular signalling pathway depending on activation of aspartate-specific proteases known as caspases^11^. Of these, Caspase-8 represents a key upstream Caspase that engages to the death-inducing signalling complex via the adaptor molecule Fas-Associated protein with Death Domain^11^. The receptor-interacting protein kinase 3 (RIPK3) and its substrate mixed lineage kinase-like (MLKL)^12^ mediate necroptosis, a newly discovered form of programmed cell death, which is activated upon tumour necrosis factor (TNF)-, antigen- or Toll-like-receptor stimulation^13^. Necroptosis contributes to various pathological conditions such as alcoholic- and methionine choline-deficient-diet-induced non-alcoholic steatohepatitis^14^,^15^, atherosclerosis^16^, Gaucher's disease^17^ and ischaemic heart and kidney injuries^18^,^19^, suggesting that this pathway might be a privileged pharmacological target in various diseases. However, the role of this pathway in obesity, metabolic syndrome and type 2 diabetes (T2D) has remained elusive. Our results show that obesity triggers RIPK3 overexpression in the WAT of mice and humans, where it dampens adipocyte apoptosis and inflammation, thereby preventing impaired insulin signalling as the basis of glucose intolerance. These data provide evidence that—in contrast to its pro-inflammatory functions in other organs and diseases—RIPK3 maintains WAT homeostasis.

## Results

### RIPK3 dampens glucose intolerance in obese mice

**Figure 1:**
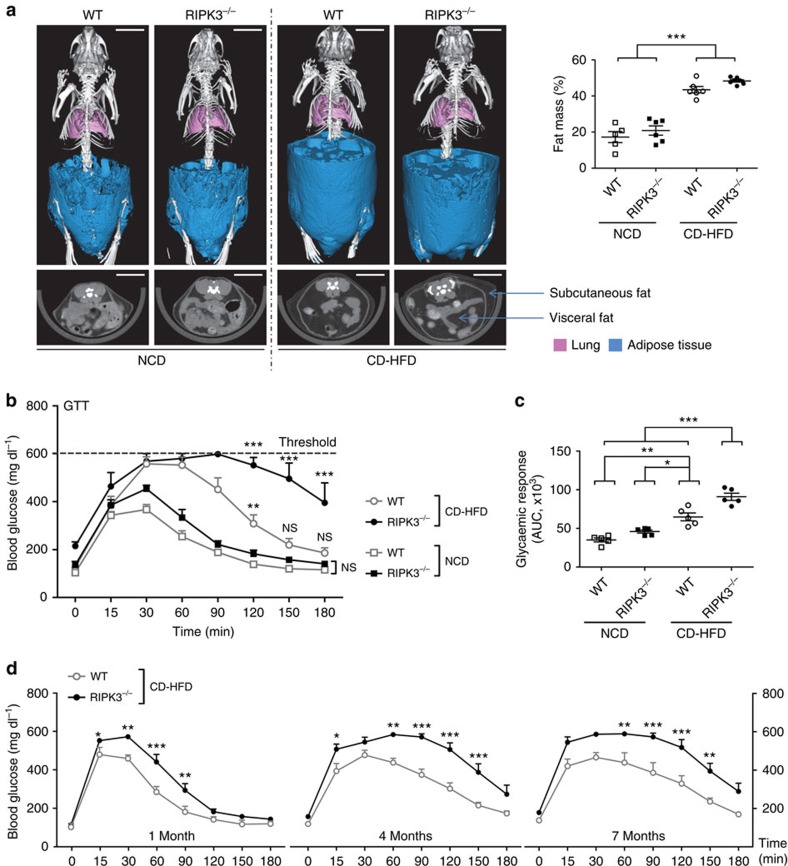
*Ripk3*deficiency induces glucose intolerance in obese mice. Data were obtained from *Ripk3* constitutive knockout mice (referred to as KO in the main text and RIPK3^−/−^ in the figures) and WT control mice fed with a NCD or a CD-HFD for 16 weeks. Differences between WT and KO mice were determined by analysis of variance (ANOVA) with Bonferroni's *post hoc* test. All data are expressed as mean±s.e.m. (**a**) Left panel: three-dimensional volume renderings of segmented bones (white), lungs (pink) and fat (blue) upon *in vivo* μCT imaging (upper panel) as well as 2D cross-sectional μCT images in transversal planes of the abdomen of the mice (lower panel). Subcutaneous and visceral fat tissue is indicated with blue arrows. Scale bar, 1 cm. Right panel: fat mass quantification (*n*=6 in each group) of WT and RIPK3^−/−^ mice after 16 weeks of NCD or CD-HFD. ***P<0.001. (**b**) Mice were examined by glucose tolerance test (GTT). Results are expressed as mean with s.e.m., **P<0.01, ***P<0.001, n.s.: not significant. (**c**) Area under the curve (AUC) for the glycaemic response was calculated using the trapezoidal rule (*n*=5 in each group). *P<0.05, **P<0.01, ***P<0.001, n.s., not significant. (**d**) WT and RIPK3^−/−^ mice fed with CD-HFD were examined at different time points 1 (*n*=4), 4 (*n*=6) and 7 (*n*=6) months with GTT. *P<0.05, **P<0.01, ***P<0.001.

To explore the functional role of RIPK3 in obesity and associated metabolic disorders, 6-week-old male C57BL/6 mice genetically ablated for *Ripk3* (knockout, KO)^20^, and age- and sex-matched wild-type (WT) control mice were fed for 16 weeks with either normal chow diet (NCD) or a choline-deficient HFD (CD-HFD), which is known to efficiently recapitulate the key features of human metabolic syndrome and non-alcoholic steatohepatitis (NASH)^21^,^22^. As shown previously^23^, KO mice on NCD displayed a slightly but significantly reduced body weight gain compared with WT controls, while upon CD-HFD feeding, WT and KO mice showed similar body weight gain over time (Supplementary Fig. 1a,b). In line, X-ray computed tomography revealed a similar and significant gain in whole-body fat mass, subcutaneous and visceral fat, upon CD-HFD feeding in WT and KO mice (Fig. 1a).

We next examined the effect of *Ripk3* deficiency on glucose homeostasis in CD-HFD-fed mice. As shown previously^21^,^22^, WT mice fed with CD-HFD displayed an impaired glucose tolerance in a standard glucose tolerance test compared with mice on NCD (Fig. 1b,c). Strikingly, CD-HFD-fed KO mice developed more pronounced glucose intolerance than WT animals (Fig. 1b,c). To evaluate the time point of impaired glucose tolerance development, we examined new independent groups of mice at 1, 4 and 7 months of CD-HFD feeding and could confirm impaired glucose tolerance in KO mice compared with WT mice at all examined time points (values in KO mice often exceeded the indicated threshold of 600 mg dl^−1^ of blood glucose (Fig. 1d,e)). Thus, genetic ablation of *Ripk3* promotes the development of glucose intolerance in obese mice. Of note, KO mice did not show significant changes in ghrelin levels and food intake compared with WT mice on CD-HFD (Supplementary Fig. 1c,d), arguing against an influence of *Ripk3* deletion on hypothalamic appetite regulation or calorie consumption as a basis for the observed phenotype. Moreover, gut microbiota composition in WT versus KO mice kept under CD-HFD was similar but differed significantly from an independent cohort of WT mice kept under NCD (Supplementary Fig. 2), arguing against a major influence of the microbiota on the phenotypic differences between CD-HFD-fed WT and KO mice.

### Impaired insulin signalling in *Ripk3*-deficient obese mice

**Figure 2:**
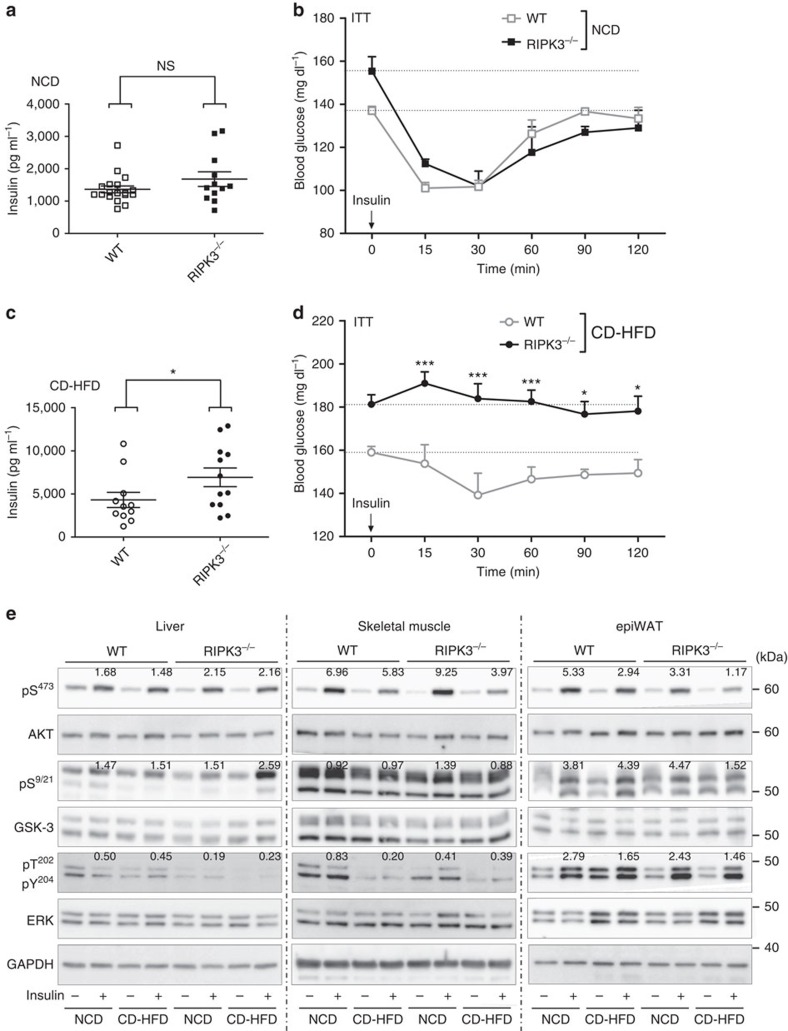
*Ripk3* deficiency promotes insulin resistance in obese mice. Data were obtained from RIPK3^−/−^ and WT mice fed with NCD or CD-HFD for different time points. Differences between WT and RIPK3^−/−^ mice were determined by ANOVA with Bonferroni's *post hoc* test. All data are shown as mean±s.e.m. (**a**) Fasting serum concentrations of insulin in NCD-fed WT (*n*=18) and RIPK3^−/−^ (*n*=12) mice. n.s., not significant. The feeding period was 16 weeks. (**b**) WT and KO mice fed with NCD diet were examined by insulin tolerance test (ITT). (*n*=3) n.s., not significant. (**c**) Fasting serum concentrations of insulin in CD-HFD-fed WT (*n*=11) and RIPK3^−/−^ (*n*=12) mice. *P<0.05. The feeding period was 16 weeks. (**d**) Obese mice fed for 24 weeks with CD-HFD were examined by ITT. *n*=6 for WT and *n*=7 for KO. *P<0.05, ***P<0.001. (**e**) WT and RIPK3^−/−^ mice fed with NCD and CD-HFD for 16 weeks were injected intraperitoneally with 1 U kg^−1^ insulin or NaCl. Ten minutes later mice were euthanized and tissue samples harvested. Immunoblot analyses of skeletal muscle (left), liver (middle) and epiWAT (right) tissue samples using antibodies specific for AKT, phospho-AKT (pS473), GSK-3, phospho-GSK-3 (pS9/21), ERK, phospho-ERK (pT202, pY204) and GAPDH as loading control. Numbers in the blot indicate relative insulin induction of AKT, GSK-3 and ERK phosphorylation.

Current theories of the pathogenesis of glucose intolerance and T2D include a defect in glucose uptake and insulin action in the skeletal muscle and liver as well as disturbed adipocyte functions^24^. Therefore, to evaluate whether one of these tissues contributes to the impaired glucose tolerance in *Ripk3*-deficient mice, we measured serum insulin levels and performed insulin tolerance tests (ITTs) in WT and KO mice fed with NCD or CD-HFD. In NCD-fed mice, we detected no significant differences in insulin levels (Fig. 2a) and ITT (Fig. 2b) between WT and KO mice. In contrast, basic insulin levels were significantly higher in obese KO compared with WT mice (Fig. 2c). Moreover, obese KO mice showed a significantly more impaired ITT than WT mice (Fig. 2d), suggesting a higher degree of insulin resistance upon *Ripk3* ablation after CD-HFD feeding.

To further characterize insulin-dependent signalling, mice were injected intraperitoneally with 1 U kg^−1^ insulin and euthanized 10 min later. Liver, skeletal muscle and epididymal WAT (epiWAT) protein samples were analysed by western blot analysis for phosphorylation of AKT, GSK3 and ERK—known intracellular mediators of insulin signalling^25^ (Fig. 2e). As expected from human T2D (ref. 26), CD-HFD feeding resulted in impaired phosphorylation of all examined mediators as a surrogate for insulin resistance in the skeletal muscle. In contrast, no clear difference was detected between WT and KO mice (Fig. 2e). In contrast, AKT and GSK3 phosphorylations in the liver tissue were not significantly influenced by CD-HFD feeding in both WT and KO mice, whereas ERK phosphorylation was impaired in KO mice (Fig. 2e). Interestingly, in epiWAT, KO mice showed a decrease in insulin-induced AKT and GSK phosphorylation on CD-HFD feeding compared with WT mice, while effects on ERK phosphorylation were not different (Fig. 2e). These findings indicated a broad spectrum of aberrations in the phosphorylation of mediators of insulin signalling in different tissues of KO mice, but also showed that *Ripk3* deletion influenced insulin sensitivity mainly in the adipose tissue.

### RIPK3 in WAT of obese mice prevents inflammation

**Figure 3:**
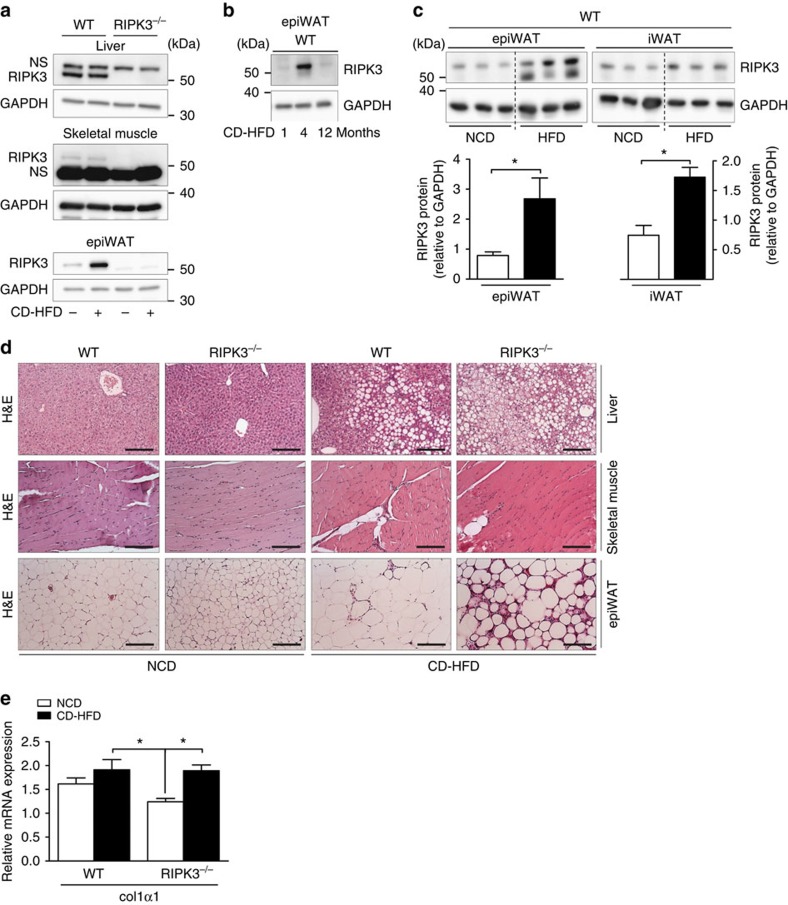
Obesity induces RIPK3 overexpression. Data were obtained from RIPK3^−/−^ and WT mice fed with NCD, CD-HFD or HFD for different time points. Differences between WT and RIPK3^−/−^ mice were determined by Student's *t*-test. All data are expressed as mean±s.e.m. (**a**) Protein levels of RIPK3 in the liver, skeletal muscle and epiWAT from lean and diet-induced obese mice. GAPDH was used as loading control. n.s., not specific. Western blots are representative of three independent experiments. (**b**) Protein levels of RIPK3 in epiWAT of mice fed for 1, 4 and 12 months with CD-HFD. (**c**) Upper panel: protein levels of RIPK3 in epiWAT and inguinal WAT (iWAT) from WT mice fed for 6 months with NCD or HFD. Lower panel: bar graphs for quantification of relative expression of RIPK3 in these respective tissues (*n*=3). *P<0.05. (**d**) Representative haematoxylin and eosin (H&E) images of liver (upper panel), skeletal muscle (middle panel) and epiWAT (lower panel) from WT and KO mice. Scale bars, 200 μm. (**e**) *col1α1* mRNA levels were assessed by RT–PCR in WT and KO fed for 16 weeks after 8 weeks of methionine choline-deficient (MCD) diet feeding. Values were calculated relative to WT mice fed with NCD, and β-actin was used as an internal standard, *n*=6 per group. Error bars represent s.e.m.

To provide additional evidence for a specific function of RIPK3 in WAT, we examined expression levels of RIPK3 in epiWAT, liver and muscle tissue of NCD- and CD-HFD-fed mice. In line with the lack of effects of *Ripk3* deletion in the liver and muscle insulin signalling, 4 months of CD-HFD feeding did not alter RIPK3 expression in the liver and skeletal muscle of WT mice. In contrast, 4 months of CD-HFD feeding led to a strong induction of RIPK3 expression in epiWAT of WT animals (Fig. 3a), suggesting a specific regulatory function in adipose tissue. Next, we tested RIPK3 expression in epiWAT at different time points of CD-HFD feeding by western blot analysis. This analysis revealed that maximum expression occurred at 4 months of age (Fig. 3b). We also wanted to examine the compartment-specific regulation of RIPK3 expression in mice fed for 6 months with a conventional HFD. As shown in Fig. 3c, RIPK3 showed a similar upregulation of RIPK3 in epiWAT on conventional HFD feeding, as seen in CD-HFD-fed mice, suggesting that, independent of choline deficiency, obesity represents a general stimulator for RIPK3 overexpression in epiWAT. Moreover, RIPK3 on 6 months of HFD was upregulated in inguinal WAT (iWAT), but less pronounced than in epiWAT, suggesting that RIPK3 upregulation in obesity occurs in a compartment-specific manner. In addition, we performed haematoxylin and eosin stainings on the liver, skeletal muscle and epiWAT to examine whether differences in RIPK3 expression went along with clear histological alterations in these respective tissues. In liver tissue, we observed a trend towards higher steatosis in KO mice compared with WT animals (Fig. 3d), while no clear differences in muscle tissue were observed between both groups (Fig. 3d). Finally, absence of *Ripk3* surprisingly was associated with a strong increase in focal areas of increased cellularity and density in epiWAT upon CD-HFD feeding compared with WT mice (Fig. 3d). This finding was not associated with increased collagen *col1α1* expression (Fig. 3e), which would indicate fibrosis, suggesting that the histological changes in the epiWAT of KO mice reflected increased inflammation.

**Figure 4:**
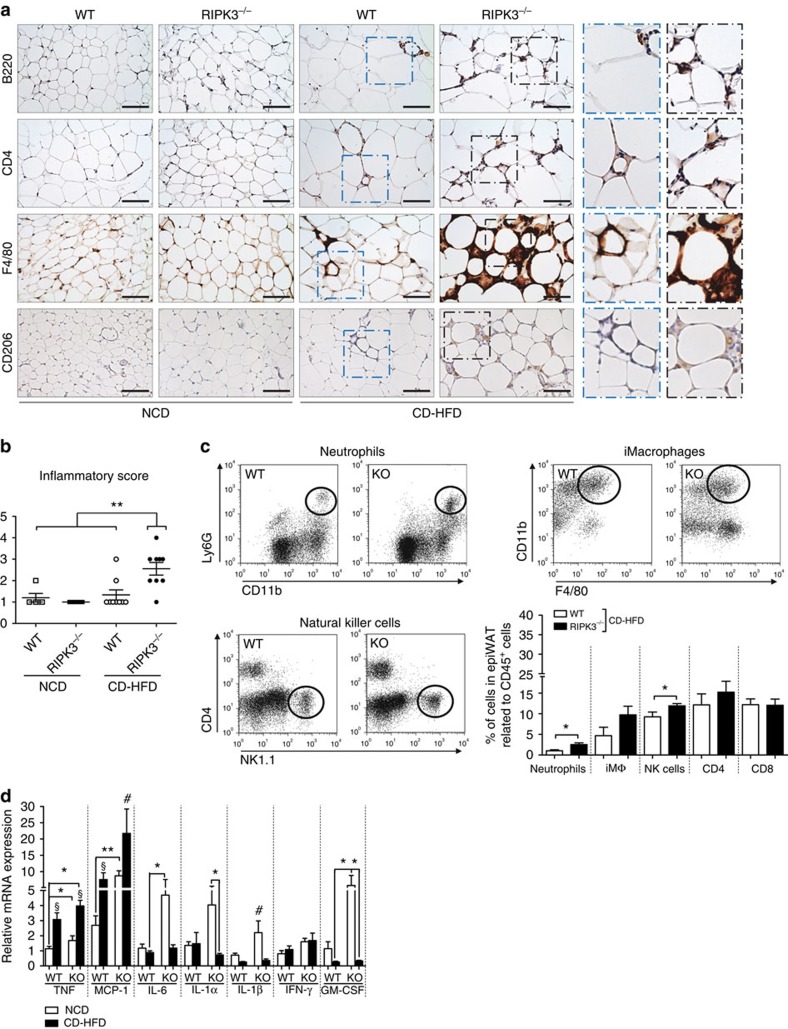
RIPK3 overexpression in obese mice prevents epiWAT inflammation. Data were obtained from RIPK3^−/−^ and WT mice fed with NCD and CD-HFD for different time points. All data are shown as mean±s.e.m. (**a**) Representative images of immunohistochemical stainings for B220^+^, CD4^+^, F4/80^+^ and CD206^+^ cells in epiWAT from WT and KO mice fed for 16 weeks with NCD and CD-HFD. Scale bars, 100 μm. (**b**) Inflammatory score of epiWAT of WT and KO mice fed for 16 weeks with NCD and CD-HFD. **P<0.01. H&E and F4/80 stains were evaluated blinded by an experienced pathologist as stated in the Methods section. Score 4, high inflammation; score 1, no inflammation. Differences between WT and KO mice were determined by ANOVA with Bonferroni's *post hoc* test. (**c**) FACS data for intra-epiWAT levels of neutrophils, inflammatory macrophages, natural killer cells, CD4-, CD8- and B-cells in WT and KO mice fed for 24 weeks with CD-HFD. *n*=6 for WT and *n*=8 for KO. *P<0.05. Differences between WT and KO mice were determined by Student's *t*-test. (**d**) *TNF*, *MCP-1*, *IL-6*, *IL-1α*, *IL-1β*, *IFN-γ* and *GM-CSF* mRNA levels were assessed by RT–PCR in WT and KO fed for 16 weeks with NCD or CD-HFD. Values were calculated relative to WT mice fed with NCD, and β-actin was used as an internal standard, *n*=6 per group. Differences between groups were determined by ANOVA with Bonferroni's *post hoc* test. § indicates that mRNA levels of *TNF* and *MCP-1* are significantly increased in KO mice compared with WT. # indicates that mRNA levels of *MCP-1* in KO mice fed with CD-HFD were significantly increased compared with all other groups. *P<0.05, **P<0.01.

Next, we performed immunohistochemical (IHC) stainings for immune cell subsets to characterize local inflammatory responses in epiWAT of WT and KO mice. As shown in Fig. 4a, obese KO mice displayed multiple focal areas comprising numerous adipocytes surrounded by macrophages and B and T lymphocytes. Inflammatory infiltrates in the WAT of these mice comprised activated M1 and M2 macrophages as demonstrated by IHC stainings for F4/80 and CD206 (Fig. 4a). In line, blinded pathological examination revealed a significantly increased inflammatory score only in the WAT of obese KO mice compared with obese WT mice and lean WT and KO mice (Fig. 4b). Moreover, fluorescence-activated cell sorting (FACS) analysis confirmed the increase in neutrophils, macrophages and natural killer (NK) cells in the WAT of obese KO mice compared with WT mice (Fig. 4c). Interestingly, it was recently shown that NK cells link obesity-induced adipose stress to inflammation and insulin resistance^27^, suggesting that NK cells might contribute to glucose intolerance in KO mice. Correlating with increased inflammation, we detected increased levels of TNF and monocyte chemoattractant protein-1 (MCP-1)—a fundamental chemokine-regulating macrophage infiltration into WAT^28^—on qRT–PCR in epiWAT of KO mice compared with WT mice (Fig. 4d). Interestingly, other cytokines such as interleukin (IL)-6, IL-1α, IL-1β and granulocyte–macrophage colony-stimulating factor (GM-CSF) were moderately upregulated in NCD-fed KO mice, but not in CD-HFD-fed KO and WT mice (Fig. 4d). While these factors obviously are not involved in the mediation of the phenotype of obese RIPK3-KO mice, they indicate that lean KO mice have an aberrant cytokine pattern in their WAT, which does not induce a significant phenotype. Of note, this difference in WAT inflammation was not accompanied by significant changes in systemic inflammation; levels of circulating cytokines, such as TNF or IL-1β, were not altered between WT and KO mice after CD-HFD feeding (Supplementary Fig. 3). Together, these data suggest that *Ripk3* deletion induced inflammation in the adipose tissue of obese mice.

### Increased adipocyte apoptosis in obese *Ripk3*-KO mice

**Figure 5:**
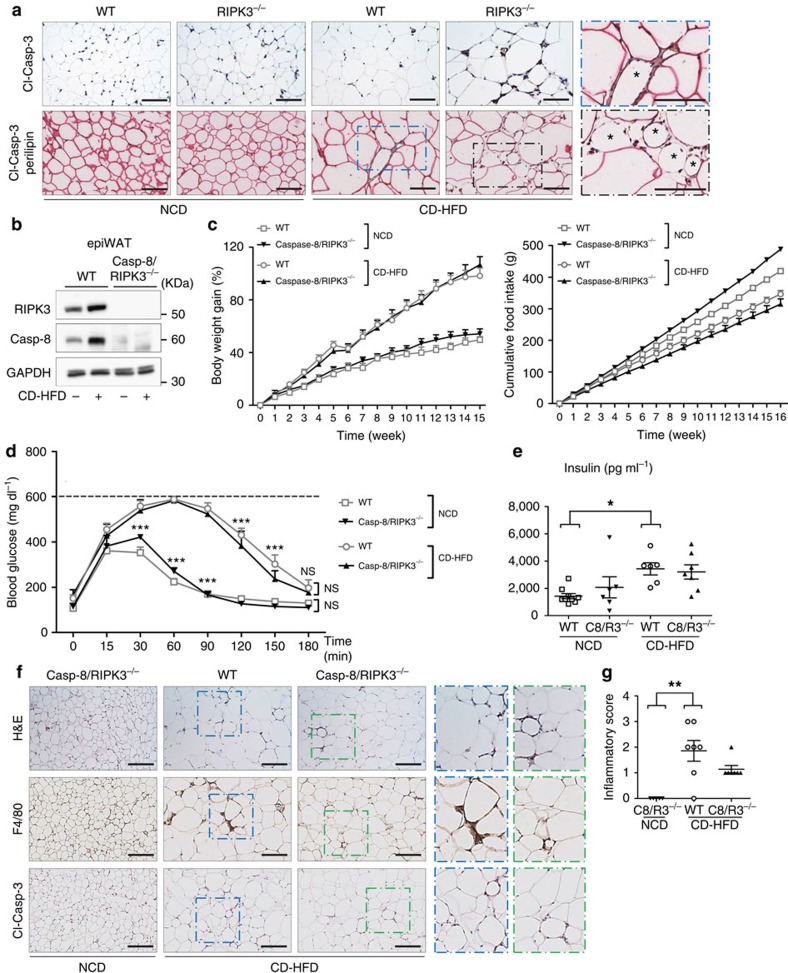
Additional *Caspase-8* deletion rescues the phenotype of *Ripk3*-KO mice. Data were obtained from Caspase-8/RIPK3 constitutive DKO (Caspase-8/RIPK3^−/−^) mice, from WT control mice and from RIPK3^−/−^/Caspase-8^LPC-KO^ fed with NCD or CD-HFD for 16 weeks. Differences between WT and DKO mice were determined by ANOVA with Bonferroni's *post hoc* test. All data are shown as mean±s.e.m. (**a**) Immunohistochemical analyses of cleaved-Caspase-3 and double stainings of cleaved Caspase-3 (brown stained) with perilipin (pink stained) in epiWAT. The asterisks designate dying adipocytes stained negatively for perilipin and positively for cleaved-Caspase-3. Scale bars, 100 μm. (**b**) Western blot analyses of RIPK3 and Caspase-8 in WT and Caspase-8/RIPK3^−/−^ mice fed with NCD or CD-HFD for 16 weeks. GAPDH is used as loading control. (**c**) Relative body weight gain (%) of WT (*n*=6) and Caspase-8/RIPK3^−/−^ (*n*=7) mice (left panel) and analysis of cumulative food intake (g) in WT (*n*=6) and DKO (*n*=7) mice after 16 weeks of feeding (right panel). (**d**) WT and Caspase-8/RIPK3^−/−^ mice were examined by GTT (*n*=5). ***P<0.001, n.s., not significant. (**e**) Fasting blood concentrations of insulin in NCD-fed WT (*n*=8) and Caspase-8/RIPK3^−/−^ (*n*=6) mice and in CD-HFD-fed WT (*n*=6) and Caspase-8/RIPK3^−/−^ (*n*=7) mice. *P<0.05. (**f**) Representative images of immunohistochemical stainings for H&E, F4/80 and cleaved-Caspase-3 in epiWAT from WT and Caspase-8/RIPK3^−/−^ mice. Scale bars, 100 μm. (**g**) Histological quantification of inflammation from IHC stains of EpiWAT of WT and Caspase-8/RIPK3^−/−^ mice. H&E and F4/80 stains were evaluated blinded by an experienced pathologist as stated in the Methods section. Score 4: high inflammation; score 1, no inflammation. **P<0.01.

It was suggested that adipocyte apoptosis is an important contributor to inflammation and insulin resistance^9^,^29^. As such, genetic inactivation of the apoptosis mediator Bid prevented adipose tissue macrophage infiltration and systemic insulin resistance in obese mice^9^. Thus, to analyse whether ablation of *Ripk3* might be associated with increased adipocyte apoptosis in WAT upon CD-HFD feeding, we performed IHC stainings for the cleaved form of Caspase-3 (Cl-Casp-3), showing an increased presence of Cl-Casp-3^+^ cells in CD-HFD-fed KO mice compared with WT mice (Fig. 5a). Apoptosis of adipocytes is associated with loss of the adipocyte-specific lipid droplet protein perilipin^9^,^30^. Therefore, to specifically identify apoptotic adipocytes, we performed a double staining for cleaved Cl-Casp-3 and perilipin, revealing numerous regions of adipocytes that were devoid of perilipin labelling and had Cl-Casp-3^+^ nuclei in KO mice when compared with WT mice (Fig. 5a). In addition, we performed a transmission electron microscopy analysis on the WAT of obese WT mice, which revealed that adipocytes showed ultrastructural signs of degeneration and cell death in CLSs (Supplementary Fig. 4). Together, these data suggest that, upon CD-HFD feeding, deletion of *Ripk3* sensitizes WAT adipocytes to apoptosis *in vivo*.

To evaluate whether glucose intolerance and inflammation in adipose tissue of KO mice are associated with the increased presence of apoptosis in the WAT of KO mice, we next used mice with combined constitutive ablation of both *Ripk3* and *Caspase-8* (double-knockout (DKO))^31^ (Fig. 5b). DKO mice are viable but develop lymphadenopathy and splenomegaly because of impaired apoptosis signalling through the death receptor Fas^31^,^32^,^33^. DKO mice showed similar body weight gain and cumulative food intake as age-, sex- and background-matched WT controls (Fig. 5c) and similar systemic levels of inflammatory cytokines (Supplementary Fig. 5). Additional genetic inactivation of *Caspase-8* protected KO mice from glucose intolerance and insulin resistance (Fig. 5d,e). In line, DKO displayed neither significant inflammation nor cleaved-Casp-3^+^ cells in their WAT (Fig. 5f,g).

We aimed at providing further evidence that *Ripk3* ablation might sensitize adipocytes towards apoptosis activation. For this, we used 3T3-L1 cells generated from murine fibroblasts that can be *trans*-activated into an adipocyte-like phenotype and are a well-established model system to study fat cells in culture^34^. Of note, stimulation of *trans*-differentiated 3T3-L1 cells with TNF alone or in combination with inhibitors of apoptosis (zVAD), RIPK1-dependent necroptosis (Nec-1s) and RIPK3 (GSK-872) did not induce any cell death (Supplementary Fig. 6a,b), suggesting that additional factors other than TNF putatively present in the peri-adipocyte milieu in obese WAT might be necessary for induction of adipocyte cell death. We therefore used another murine fibroblast cell line, L929, which is known to be sensitive to necroptosis upon TNF stimulation^35^. On TNF stimulation or in combination with zVAD, L929 cell death showed morphological signs of necroptosis but not apoptosis (Supplementary Movies 1 and 2). As shown in Supplementary Fig. 7, treatment of L929 cells with GSK-872 at a low dose (without spontaneous toxicity at this dose) induced apoptosis upon TNF treatment, which was rescued by additional zVAD treatment (Supplementary Fig. 7). Moreover, western blot analysis of epiWAT at 4 months of CD-HFD feeding showed that, similarly to RIPK3, Caspase-8 expression was strongly induced (Supplementary Fig. 6c), suggesting that absence of RIPK3 might tip the balance of cell death towards apoptosis. Finally, based on our *in vivo* data on RIPK3 upregulation in WT WAT, we engineered an adenoviral vector for overexpression of murine RIPK3 before and after *trans*-differentiation of 3T3-L1 cells and measured necroptosis using an antibody detecting the phosphorylated version of the executer MLKL (Supplementary Fig. 6d,e). Interestingly, phosphorylation of MLKL was only detected before *trans*-differentiation, arguing that RIPK3 can be overexpressed in adipocytes without cell toxicity (Supplementary Fig. 6d,e). Collectively, these findings provide evidence that RIPK3 prevents glucose intolerance in obese mice by inhibiting Caspase-8-dependent adipocyte apoptosis in WAT.

### The *Ripk3*-KO phenotype is not mediated by immune cells

**Figure 6:**
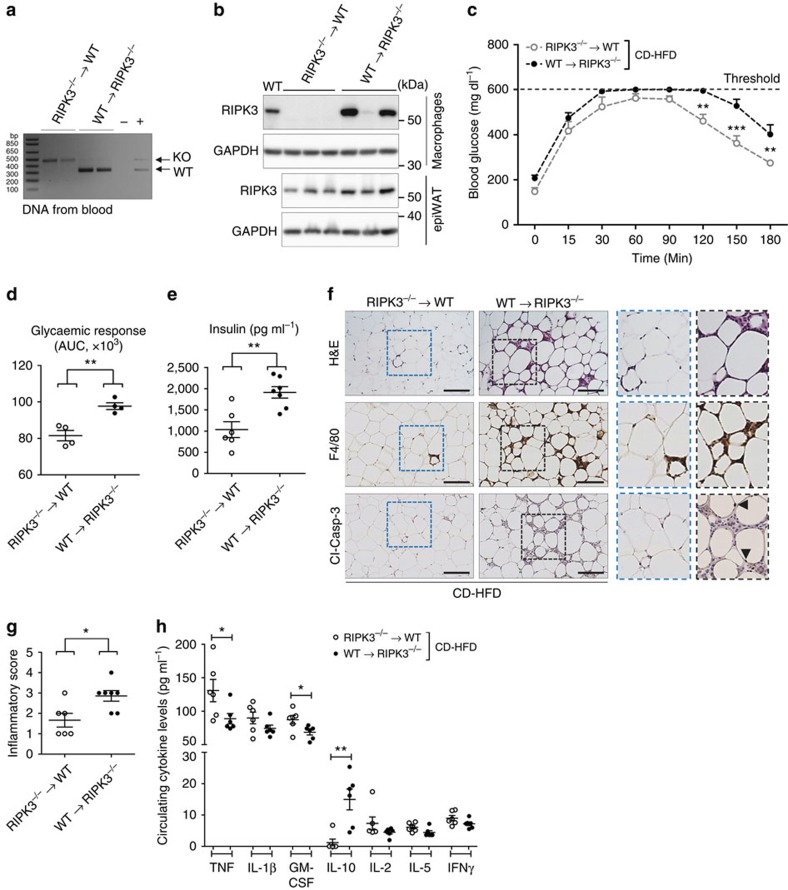
The function of immune cells in the metabolic *Ripk3*-KO phenotype. Data were obtained from RIPK3^−/−^ mice reconstituted with WT bone marrow upon irradiation at the age of 6 weeks (WT→RIPK3^−/−^) and WT mice reconstituted with *Ripk3*-deficient bone marrow (RIPK3^−/−^→WT). Two weeks after BM transfer, mice were put on CD-HFD for 16 weeks. Differences between groups were determined by Student's *t*-test. All data are expressed as mean±s.e.m. (**a**) PCR analysis from whole-blood DNA using primers indicating the presence or absence (KO) of WT *Ripk3*. (**b**) Western blot analysis of RIPK3 from bone marrow-derived macrophages and adipose tissue of WT→RIPK3^−/−^ and RIPK3^−/−^→WT animals. GAPDH is used as a control loading. (**c**) Mice were examined by GTT. **P<0.01, ***P<0.001. (**d**) AUC for the glycaemic response was calculated using the trapezoidal rule (*n*=4 in each group). **P<0.01. (**e**) Fasting blood concentrations of insulin in RIPK3^−/−^→WT (*n*=6) and WT→RIPK3^−/−^ (*n*=7). **P<0.01. (**f**) Representative images of histological and immunohistochemical stainings for H&E, F4/80^+^ and Cl-Casp-3^+^ cells in epiWAT from RIPK3^−/−^→WT and WT→RIPK3^−/−^ animals. Arrowheads indicate cl-Casp-3^+^ nuclei of adipocytes. Scale bars, 100 μm. (**g**) Histological quantification of inflammation from the IHC stains H&E and F4/80 stains were evaluated blinded by an experienced pathologist as stated in the Methods section. Score 4: high inflammation; score 1, no inflammation. *P<0.05. (**h**) Fasting serum concentrations of TNF, IL-1β, GM-CSF, IL-10, IL-2, IL-5 and IFN-γ in CD-HFD-fed RIPK3^−/−^→WT (*n*=6) and WT→RIPK3^−/−^ (*n*=7) mice were assessed using Bio-plex Assay. *P<0.05, **P<0.01.

Despite the results in DKO mice, it is possible that RIPK3 signalling in immune cells contributes to the metabolic phenotype of KO mice on CD-HFD feeding. As such, in CD-HFD-fed WT mice, macrophages in WAT could undergo necroptosis, which may limit inflammation. To functionally test this hypothesis, we transferred WT bone marrow (BM) into irradiated KO mice (WT→RIPK3^−/−^) as well as the BM from *Ripk3*-KO mice into WT mice (RIPK3^−/−^→WT; Fig. 6a). As expected, isolated macrophages from the BM of RIPK3^−/−^→WT mice at the end of CD-HFD feeding (18 weeks after bone marrow transfer (BMT)) showed no RIPK3 expression, while macrophages from WT→RIPK3^−/−^ mice showed RIPK3 (Fig. 6b). More importantly, all WT→RIPK3^−/−^ mice showed RIPK3 expression in their epiWAT on western blot analysis (Fig. 6b), arguing that WT bone-marrow-derived immune cells infiltrated into the WAT of obese WT→RIPK3^−/−^ mice. Interestingly, WT→RIPK3^−/−^ mice after 16 weeks of CD-HFD presented with a significantly stronger glucose intolerance and higher insulin levels than RIPK3^−/−^→WT animals (Fig. 6c–e). In line with this, they displayed more macrophage infiltration and apoptosis in their WAT (Fig. 6f,g). On a systemic level, both BMT lines showed similar levels of inflammatory cytokines (Fig. 6h), and TNF levels in RIPK3^−/−^→WT were even slightly increased compared with WT→RIPK3^−/−^ mice, while IL-10 showed a reciprocal downregulation. Importantly, primary macrophages isolated from WT and KO mice did not show any difference in the expression of inflammatory cytokines (TNF, IL-1α and IL-6) or Cyclin D1 when stimulated with TNF (Supplementary Fig. 8a,b), arguing against altered cytokine expression of *Ripk3*-deficient macrophages. Moreover, supernatants from scratched cultured 3T3-L1 cells only without clearance of cell debris resulted in a similar transcriptional activation of IL-6 and Cyclin D1 (Supplementary Fig. 8a,b), suggesting that debris from dead adipocytes might be involved in the inflammatory activation in the WAT of KO mice (Supplementary Fig. 8c). Together, these findings provided evidence that adipocyte apoptosis is an important driver of glucose intolerance in CD-HFD-fed KO mice.

### *Ripk3*-deficient hepatocyte apoptosis in glucose intolerance

**Figure 7:**
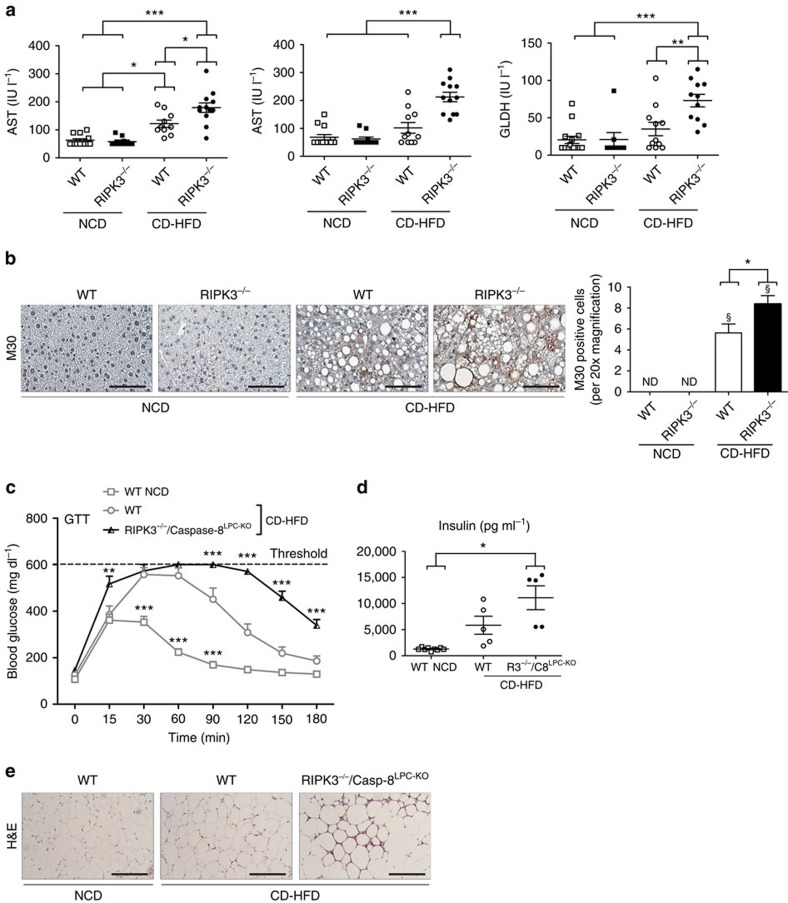
Increased liver injury and apoptosis in *Ripk3*-deficient livers. Data were obtained from WT, KO and RIPK3^−/−^/Caspase-8^LPC-KO^ mice fed with NCD or CD-HFD for 16 weeks. Differences between the mice were determined by ANOVA with Bonferroni's *post hoc* test. All data are shown as mean±s.e.m. (**a**) Analysis of serum levels of AST, ALT and GLDH after 16 weeks of NCD or CD-HFD for WT and KO mice (*n*=11). *P<0.05, **P<0.01, ***P<0.001. (**b**) Left panel: representative images of immunohistochemical staining for M30 cells in the liver from WT and KO mice fed for 16 weeks with NCD and CD-HFD. Scale bars, 100 μm. Right panel: statistical analysis of M30-positive hepatocytes (*n*=6). *P<0.05, ND, non detectable. (**c**) RIP3^−/−^/Caspase-8^LPC-KO^ mice fed with CD-HFD were examined by GTT and compared with WT animals fed either with NCD or CD-HFD (*n*=5). **P<0.01, ***P<0.001. (**d**) Fasting serum concentrations of insulin in NCD-fed WT mice (*n*=9) and in CD-HFD-fed WT and RIPK3^−/−^/Caspase-8^LPC-KO^ mice (*n*=5) mice. *P<0.05. (**e**) Representative H&E images of epiWAT from WT animals fed with NCD and WT and RIPK3^−/−^/Caspase-8^LPC-KO^ mice fed with CD-HFD for 16 weeks. Scale bars, 100 μm.

On the basis of these previous findings, we next examined whether systemic glucose intolerance in KO mice was associated with increased signs of NAFLD and NASH. Blinded histological analysis (NAS score) revealed a nonsignificant trend to higher NAS score in KO mice compared with WT mice after 4 months of CD-HFD feeding (Supplementary Table 1). While, in marked difference to WAT, inflammation was not increased in KO livers, they showed a higher lipid content than WT livers (Supplementary Table 1). Further analysis of serum markers of liver injury (aspartate aminotransferase (AST), alanine aminotransferase (ALT) and glutamate dehydrogenase (GLDH)) revealed a moderate increase in KO mice (Fig. 7a) that went along with increased numbers of hepatocytes that stained positive for the apoptosis marker cytokeratin-18-cleavage product M30 (Fig.7b). This finding indicated that—similarly to WAT—ablation of *Ripk3* led to a switch towards apoptosis in liver cells. On the basis of these findings, we next tested whether additional deletion of Caspase-8 in hepatocytes (Caspase-8^LPC-KO^) could also rescue KO mice from glucose intolerance (RIPK3^−/−^/Caspase-8^LPC-KO^). Importantly, this approach did not rescue glucose intolerance and insulin resistance of KO mice (Fig. 7c,d). Moreover, RIPK3^−/−^/Caspase-8^LPC-KO^ mice still showed the same level of WAT inflammation as single KO mice (Fig. 7e). These findings suggested that *Ripk3* ablation has a similar effect in adipocytes and hepatocytes upon CD-HFD feeding. However, our genetic and functional data indicated that the effects of *Ripk3* deletion in WAT are the primary determinants of the metabolic phenotype on CD-HFD feeding.

### RIPK3 is overexpressed in WAT of obese humans and mice

**Figure 8:**
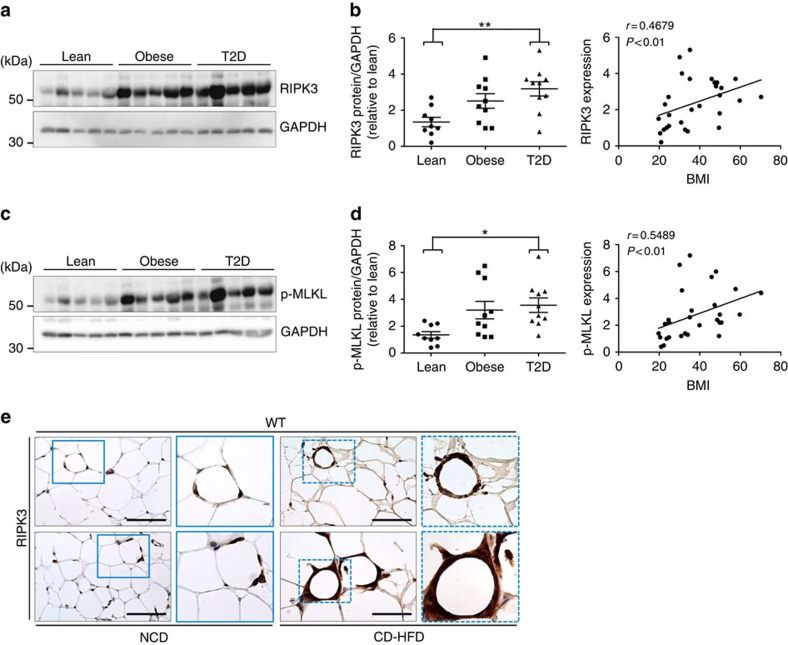
Activation of RIPK3-dependent signalling pathways in obesity. Data were obtained from lean controls, obese non-diabetic patients and obese patients with T2D. WT mice were fed with CD-HFD for 4 months. Differences between groups were determined by ANOVA with Bonferroni's *post hoc* test and correlations were assessed by non-parametric Spearman's test. All data are shown as mean±s.e.m. (**a**) Representative western blot analysis of RIPK3 on protein lysates of the visWAT from lean controls and obese and T2D patients (*n*=5 for each group). GAPDH is used as a loading control. (**b**) Left panel: relative protein quantification of RIPK3 levels in the different groups of human samples (*n*=10 for each group in total), standardized to the loading control GAPDH. **P<0.01. Right panel: correlation analysis between RIPK3 protein levels and body mass index (BMI). **P<0.01. (**c**) Representative western blot analysis of p-MLKL in the visWAT of lean controls and obese and T2D patients (*n*=5 for each group). GAPDH is used as a loading control. (**d**) Left panel: relative protein quantification of p-MLKL levels in the different groups of human samples (*n*=10 for each group), standardized to the loading control GAPDH. *P<0.05. Right panel: correlation analysis between p-MLKL protein levels and the BMI. **P<0.01. (**e**) Representative images of immunohistochemical stainings for RIPK3 in epiWAT from WT mice fed 16 weeks with NCD and CD-HFD. Scale bars, 100 μm.

We finally assessed whether RIPK3 expression is also altered in the visceral fat tissue (visWAT) of human patients with obesity and T2D (Supplementary Table 2). Similarly to obese mice, we found an upregulation of RIPK3 in the visWAT of obese patients with and without T2D compared with non-obese controls (Fig. 8a,b). We also evaluated whether RIPK3 upregulation in the visWAT of obese and diabetic patients was associated with activation of necroptosis. To test this, we performed western blot analyses with an antibody against phosphorylated MLKL, which is considered to be the best available biomarker for activation of necroptosis^36^. Interestingly, MLKL phosphorylation closely followed RIPK3 overexpression in the visWAT of these patients (Fig. 8c,d and Supplementary Fig. 9a) and it also showed a correlation with metabolic serum markers (Supplementary Fig. 9b), suggesting that necroptosis is activated in the WAT of obese humans. Of note, RIPK3 expression correlated with metabolic serum markers, such as HbA1c and levels of circulating insulin, in these patients (Supplementary Fig. 9c); however, it is presently unclear whether the downstream effects of RIPK3 inhibition in humans would be similar to our findings in mice, requiring further investigations. Given the upregulation that we had detected in western blot analyses on WAT of obese humans and mice, we finally wanted to confirm histologically in murine WAT that adipocytes can upregulate RIPK3. As hypothesized, this analysis revealed high expression of RIPK3 (Fig. 8e) in WT mice, suggesting that, next to apoptosis, obesity triggers activation of RIPK3-dependent signalling in adipocytes.

## Discussion

**Figure 9:**
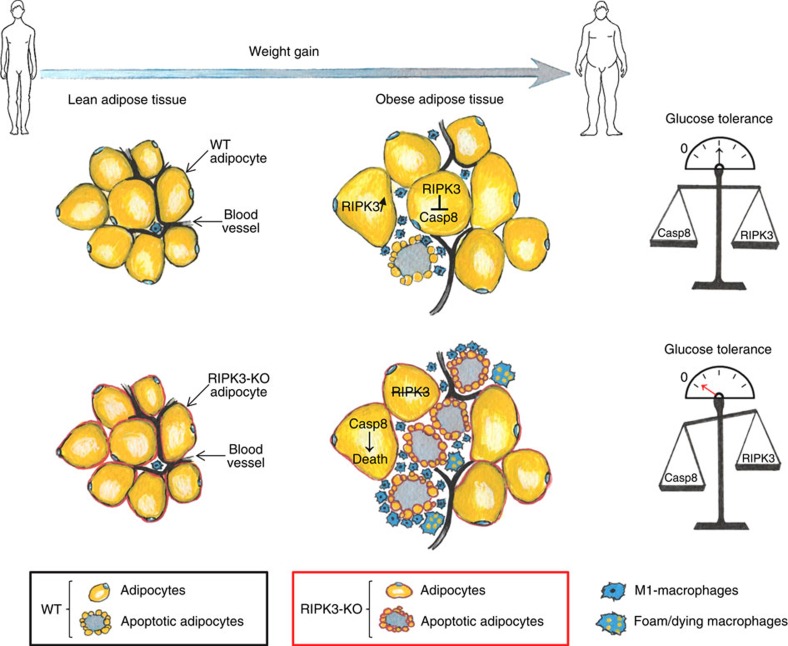
RIPK3 dampens WAT apoptosis and systemic glucose intolerance. In WT mice, weight gain induced overexpression of RIPK3 in adipocytes that counterbalanced the activation of Caspase-8-dependent apoptosis, thereby dampening WAT inflammation and glucose intolerance.

Our data in obese mice suggest that RIPK3 in adipose tissue maintains homeostasis and dampens inflammation by inhibiting Caspase-8-dependent apoptosis of adipocytes, thereby preventing glucose intolerance (Fig. 9). In many tissues, RIPK3 upregulation is known to sensitize cells to necroptosis, which triggers inflammation^37^,^38^. However, it has been suggested that in certain tissues and disease models necroptosis might not be associated with induction of inflammation^39^,^40^,^41^ or might even inhibit the pro-inflammatory response to apoptosis^42^. Thus, apoptosis and necroptosis might either trigger or block inflammation, depending on the cell type and cell death stimulus, and specific forms of programmed cell death in adipose tissue have opposing influences on systemic glucose homeostasis.

Importantly, our findings together with previous studies suggest that different forms of regulated cell death can be detected simultaneously in the WAT of obese mice and humans^8^,^9^. The finding that *Ripk3* deletion triggers increased apoptosis in mice is unexpected and might be related to the specific trigger of cell death (triglyceride overload) or the specific cell type (adipocytes). Of note, it was shown previously that mice with constitutive ablation of *Ripk1* display spontaneous apoptosis in the lymphoid and adipose tissue^43^, suggesting that adipocytes might be very sensitive to a switch towards apoptosis on genetic or pharmacological modulations of RIPK3 (ref. 44). Together, these data support the hypothesis that RIPK3 can have an anti-apoptotic function in certain circumstances, which might be of relevance when considering RIPK3 inhibition as a pharmacological strategy for treatment of necroptosis-related diseases. Interestingly, our data pointed towards an age-related expression pattern of RIPK3, with highest expression at 4 months of CD-HFD feeding. Age-specific differences in WAT expression have been shown for numerous genes^45^. Of note, expression of Caspase-8 remained high even at older age (Supplementary Fig. 10), suggesting that loss of the protective effect of even lower RIPK3 levels might be sufficient to trigger apoptosis also at that age.

In addition to adipocyte apoptosis, we detected at a lower extent the apoptosis of innate immune cells in the inflamed WAT of obese mice as demonstrated by IHC (nuclei of immune cells stained for Cl-Casp-3 (Fig. 5)) and transmission electron microscopy (macrophage with cytoplasmic signs of degeneration (Supplementary Fig. 4)). While our BMT experiments argued against a primary role of immune cells in the phenotype, it was previously demonstrated in models of atherosclerosis that macrophages can form foam cells containing lipid droplets that can undergo necroptosis as well as apoptosis^16^,^46^,^47^, thereby aggravating and perpetuating the inflammatory process. In line, our RIPK3-western blots in the WAT of mice that had undergone BMT (Fig. 6b) confirmed that RIPK3 was expressed in both adipocytes and immune cells. A similar process might therefore occur in the WAT of KO mice. Importantly, it was previously shown that adipocyte death is sufficient to initiate macrophage infiltration^48^, supporting our data that adipocyte apoptosis in KO mice is the main trigger for inflammation, which can be blunted by additional deletion of Caspase-8 (Fig. 5). In the same line, it was previously suggested that induction of fat cell apoptosis—for example, by applying Leptin, TNF or natural compounds—might represent a novel therapeutic strategy against obesity and a realistic alternative to caloric restriction in order to achieve fat loss^49^. However, our present findings in *Ripk3*-deficient mice underline that activation of apoptosis in WAT adipocytes is linked with detrimental unwanted metabolic side effects including inflammation and glucose intolerance. It is presently unclear and might be interesting to determine whether activation of necroptosis might be an alternative in this respective setting. It is unexpected that the strong local differences in WAT inflammation were not associated with a marked difference in the systemic inflammatory markers between WT and KO mice (Supplementary Fig. 3). However, it is possible that these differences might get clearer at later time points of CD-HFD or that alternative factors and adipokines other than the ones we measured connected local WAT inflammation with systemic glucose intolerance.

Our findings on the association between RIPK3 overexpression and obesity and metabolic serum parameters in humans clearly indicated that RIPK3 has a similar function in the WAT of obese humans and mice. Of note, we showed that RIPK3 overexpression in human patients strictly correlated with phosphorylation of MLKL, the direct target of RIPK3 mediating necroptosis. It was previously shown that adipocyte apoptosis is markedly increased in omental fat from obese subjects, correlating with the magnitude of adipose tissue infiltration by macrophages and systemic markers of insulin resistance^9^. Moreover, it is known that TNF is strongly expressed in adipocytes of obese subjects and correlates with body mass index (BMI)^50^, providing further evidence for the hypothesis that necroptosis in WAT might primarily act to limit immune responses triggered by inflammatory cytokines such as TNF or initiated and aggravated by adipocyte apoptosis^9^.

We and others recently showed that in patients with NASH—an important complication of obesity and major risk factor for the development of liver cirrhosis and hepatocellular carcinoma in western industrialized countries^51^,^52^—hepatocytes with the phosphorylated form of MLKL can be detected^53^,^54^. Given that pharmacological inhibition of apoptosis was even tested in clinical trials with NASH patients^55^, targeting of necroptosis appeared a promising new strategy in this respective clinical context. However, our present findings on the unexpected role of RIPK3 in WAT suggest that systemic pharmacological targeting of RIPK3 in NASH patients—as well as in other necroptosis-related diseases^10^—might cause or aggravate glucose intolerance and insulin resistance in obese individuals.

## Methods

### Human material

Samples of visceral (omental) adipose tissue were obtained from 30 individuals (19 women and 11 men; Supplementary Table 2). Their age ranged from 22 to 78 years and the BMI from 16 to 70.3 kg m^−2^. The entire cohort was subdivided into subgroups of healthy lean (BMI<25 kg m^−2^; *n*=10) individuals and patients with obesity (BMI>30 kg m^−2^; *n*=20) either with (*n*=10) or without (*n*=10) T2D. The latter two subgroups were matched for age and BMI. All adipose tissue samples were collected during open or laparoscopic abdominal surgery as described previously^56^. Adipose tissue was immediately frozen in liquid nitrogen and stored at −80 °C. The study was approved by the Ethics Committee of the University of Leipzig (approval no: 159-12-21052012), and was performed in accordance to the declaration of Helsinki. All subjects gave written informed consent before taking part in this study. Measurement of body composition, metabolic parameters and adipose tissue-related parameters was performed as described previously^56^.

### Animals

*Ripk3*-deficient mice were obtained from Genentech^20^, and *Caspase-8/Ripk3*-deficient mice were described previously^31^. For the breeding of *Ripk3*-KO- and WT mice, heterozygous co-founders (C57BL/6) were interbred. +/+ and −/− mice from the F1 generation were separated and interbred in parallel. The phenotype was analysed from the F4 generation on. Mice were housed under exactly the same conditions (cage inlays were exchanged between cages once a week during the experiments). Mice with constitutive deletion of *Ripk3* and conditional deletion of *Caspase-8* in the liver (liver parenchymal cell knockout (LPC-KO)) were described previously^14^. For these, age- and sex-matched littermates were used as controls. Animals were allocated to the experimental groups based on their genotypes. All animal experiments were approved by the Federal Ministry for Nature, Environment and Consumers' Protection of the state of North Rhine-Westphalia and were performed in accordance to the respective national, federal and institutional regulations.

### Diet

In all experiments, 6-week-old male mice were fed with a CD-HFD (Research Diets; D05010402) or with a NCD for varying time intervals as indicated. Mice were single housed, and food intake and weight body gain were measured weekly by weighing food hoppers and animals, respectively. At the end of the feeding protocol, mice were fasted at least for 6 h before being euthanized. Similarly, in Fig. 3c, fat samples were gained from mice that had been fed for 6 months with NCD or conventional HFD (Research Diets; D12492).

### Faecal Microbiota Analysis

Faecal samples were collected from mice with different genotype, diet and time points. DNA from ∼20 mg faecal pellets was isolated with the QIAamp DNA Stool Mini Kit (QIAGEN) as instructed by the manufacturer with additional bead beating steps. For in-depth microbiota profiling, 16S rRNA gene V4 region amplicons from faecal DNA isolates were amplified by the 515F/806R primers and sequenced on an Illumina MiSeq platform using 2 × 300 bp according to the established protocols^57^. Data were analysed using the QIIME software pipeline (v1.9.1)^58^. The UCLUST algorithm was used with default variables to cluster the sequences into operational taxonomic units based on 97% identity against the Greengenes reference database (version 13_8)^59^. The diversity between samples was measured by the weighted UniFrac distance, and a principal coordinate analysis of the weighted UniFracs was calculated and constructed with QIIME.

### Imaging

*In vivo* μCT imaging of WT and RIPK3^−/−^ mice fed with NCD or CD-HFD (*n*=6–8) for 16 weeks was performed using a dual-energy gantry-based flat-panel microcomputed tomography scanner (TomoScope 30s Duo, CT Imaging, Erlangen, Germany)^60^. The dual-energy X-ray tubes of the μCT were operated at voltages of 40 and 65 kV with currents of 1.0 and 0.5 mA, respectively. To cover the entire mouse, three sub-scans were performed, each of which acquired 720 projections with 1,032 × 1,012 pixels during one full rotation with durations of 90 s. Animals were anaesthetized using 2% isoflurane in air for the entire imaging protocol (flow rate 1 l min^−1^). After acquisition, volumetric data sets were reconstructed using a modified Feldkamp algorithm with a smooth kernel at an isotropic voxel size of 35 μm. The fat-containing tissue regions, which appear hypo-intense in the μCT data, were segmented using an automated segmentation method with interactive correction of segmentation errors^61^,^62^. The volumetric fat percentage was computed as the ratio of (subcutaneous and visceral) fat volume to the entire body volume.

L929 cells were seeded in standard 12-well cell culture plates with 1.5 ml of DMEM and were subjected to *in vitro* time-lapse microscopy, using an AxioObserver Z1 (Zeiss) modified with large chamber cell culture incubation unit (Pecon). After 1 h of live imaging, cells were stimulated by adding zVAD (20 μM) or dimethylsulphoxide for 1 h and then TNF (20 ng ml^−^). Cells were followed over a time period of up to 7–8 h, taking images from eight different target positions within the well every 3 min. Cells were kept at 37 °C and 5% CO_2_ atmosphere over the time course of the experiment. Time-lapse movies were generated using ZEN Blue 2.0 (Zeiss).

### Metabolic studies

Mice were fasted overnight for 16 h. Then, 2 mg of glucose per g body weight was intraperitoneally injected and tail blood was taken at the indicated time points. Glucose levels were determined using a hand-held glucose analyser (Contour XT Bayer). Insulin (1 U kg^−1^) was intraperitoneally injected for 10 min. Mice were then euthanized and tissues were collected for western blot analysis. Insulin tolerance tests were performed on tail blood taken at the indicated time points.

### Serum analysis

Insulin and ghrelin levels were measured using suspension bead array immunoassay kits following the manufacturer's specifications (Bio-Plex pro Mouse Diabetes Assay 8-plex, Bio-Rad) on a Luminex 100/200 analyser. Bio-Plex Pro Mouse Cytokine GrpI panel 8-plex kit was used to measure TNF, IL-1β, IL-2, IL-5, IL-10, GM-CSF and interferon (IFN)-γ in the serum of mice. The data were analysed with Bio-Plex Manager Instrument Control Version 6 (Bio-Rad). Measurement of body composition, metabolic parameters and adipose tissue-related parameters from human patients was performed as described previously^56^.

### BM transplantation

BM from WT and RIPK3^−/−^ donors (*n*=4 mice per group) was transplanted into 6-week-old RIPK3^−/−^ and WT recipient mice, respectively. After ablative γ-irradiation of the recipient mice, 2.5 × 10^6^ of the BM cells from donors were injected via the tail vein. After BM transfer, mice were treated with antibiotics (0.02% Borgal) for 2 weeks before the feeding experiments were started. At the end of the feeding experiment, DNA from the blood of recipient mice was extracted using the QIAamp kit (Qiagen) and analysed for confirming the success of the BM transfer.

### Western blot analysis

Tissue samples were homogenized in NP-40 lysis buffer using a tissue grind pestle (Kimble/Chase) or with a bead ruptor 12 (Omni International) to obtain protein lysates. These were separated by SDS–PAGE, transferred to polyvinylidene difluoride membrane and analysed by immunoblotting. Membranes were probed with the following antibodies: anti-p-AKT (#4060), anti-AKT (#4685), anti-p-GSK-3 (#8566), anti-GSK-3 (#5676), anti-p-ERK (#4377) and anti-ERK (#4695; Cell Signaling), anti-Caspase-8 (Enzo #ALX-804-447), anti-RIPK3 mouse (IMGENEX #IMG-5523), anti-RIPK3 human (#ab56164), anti-phospho-MLKL human (#ab187091) and mouse (#ab196436; Abcam) and anti-GAPDH (ABD Serotec #MCA4739). All primary antibodies were used at the dilution 1:2,000. As secondary antibodies, anti-rabbit-horseradish peroxidase (HRP; #NA934V) and anti-mouse-HRP (#NA931V; Amersham) and anti-rat-HRP (Santa Cruz #sa2956) were used. All secondary antibodies were used at the dilution 1:5,000. Unedited scans of all western blot images are shown in Supplementary Fig. 11.

### Quantitative real-time PCR

Total RNA was purified from fat tissue using Trizol reagent (Invitrogen) and an RNeasy Mini Kit (Qiagen). The quantity and quality of the RNA was determined spectroscopically using a nanodrop (Thermo Scientific). Total RNA (0.5 μg) was used to synthesize cDNA using the Transcriptor cDNA First-Strand Synthesis Kit (Roche) according to the manufacturer's protocol. cDNA samples (2 μl) were used for real-time PCR in a total volume of 25 μl using SYBR Green Reagent (Invitrogen) and specific primers on a qPCR machine (Applied Biosystems 7,300 Sequence Detection System). All real-time PCR reactions were performed in duplicates. Data were generated and analysed using the SDS 2.3 and RQ manager 1.2 software. Primer sequences are available on request. All values were normalized to the level of beta-actin mRNA. The expression of TNF, MCP-1, IL-6, IL-1α, IL-1β, CycD1, IFN-γ, GM-CSF and col1α1 was tested using the primers as follows: TNF: 5′- ACCACGCTCTTCTGTCTACTGA -3′ (for), 5′- TCCACTTGGTGGTTTGCTACG -3′ (rev); MCP-1: 5′- GTGTTGGCTCAGCCAGATGC -3′ (for), 5′- GACACCTGCTGCTGGTGATCC -3′ (rev); IL-6: 5′- GCTACCAAACTGGATATAATCAGGA -3′ (for), 5′- CCAGGTAGCTATGGTACTCCAGAA -3′ (rev); IL-1α: 5′- GCACCTTACACCTACCAGAGT -3′ (for), 5′- AAACTTCTGCCTGACGAGCTT -3′ (rev); IL-1β: 5′- GCAACTGTTCCTGAACTCAACT -3′ (for), 5′- ATCTTTTGGGGTCCGTCAACT -3′ (rev); CycD1: 5′- GCGTACCCTGACACCAATCTC -3′ (for), 5′- CTCCTCTTCGCACTTCTGCTC -3′ (rev); IFN-γ: 5′- ATGAACGCTACACACTGCATC -3′ (for), 5′- CCATCCTTTTGCCAGTTCCTC -3′ (rev); GM-CSF: 5′- GGCCTTGGAAGCATGTAGAGG -3′ (for), 5′- GGAGAACTCGTTAGAGACGACTT -3′ (rev); col1α1: 5′- GCTCCTCTTAGGGGCCACT -3′ (for), 5′- CCACGTCTCACCATTGGGG -3′ (rev).

### Histological examination and evaluation

Paraffin sections (2 μm) were stained with haematoxylin and eosin or various primary and secondary antibodies. Paraformaldehyde (4%) fixed and paraffin embedded liver, skeletal muscle and epiWAT were incubated in Bond Primary antibody diluent (Leica) and stainings were performed on a BOND-MAX immunohistochemistry robot (Leica Biosystems) using BOND polymer refine detection solution for DAB. The following antibodies were used: anti-F4/80 (BMA Biomedicals AG, 1:120), anti-CD206 (AbD Serotec 1:200), anti-B220 (BD Biosciences; 1:3,000), anti-CD4 (eBioscience; 1:1,000), anti-cl-Casp-3 (Cell Signaling; 1:300), anti-perilipin (RDI Division of Fitzgerald, 1:1,000), anti-RIPK3 (Abcam; 1:500) and anti-p-MLKL (Abcam; 1:500). Image acquisition was performed on an Olympus BX53 microscope with a Leica SCN400 slide scanner. Stains were evaluated blinded by an experienced pathologist and inflammatory scores were determined using the following system: 0=basically no inflammation, 1≤400-fold field of view, 2=400–200-fold field of view, 3⩾200–100-fold field of view, 4=up to 40-fold field of view. The histological scoring system for non-alcoholic fatty liver disease (NAFLD) was performed according to the NAS score system^63^.

### Transmission electron microscopy

Small adipose tissue fragments from lean and obese mice were fixed in 2% gluteraldheyde–2% paraformaldehyde in phosphate buffer for 4 h at room temperature, and then post-fixed in 1% osmium tetroxide and embedded in an Epon-Araldite mixture. Semithin sections (2 μm) were stained with toluidine blue. Thin sections were obtained with an MT-X ultratome (RMC; Tucson, AZ), stained with lead citrate and examined with a CM10 transmission electron microscope (Philips; Eindhoven, the Netherlands).

### Cell isolation and flow cytometry

To isolate cells from WAT, the tissue was minced into small pieces sizing below 1 mm. Lymph nodes were excised before mincing. Next, the tissue was digested for 30 min with 2% collagenase IV (Worthington) in Hank's buffered salt solution (HBSS). HBSS was supplemented with 5 μM ethylene-diamine-tetra-acetic acid and 0.5% bovine serum albumin to stop the collagenase activity. To prepare for flow cytometry, cells were filtered using a 100-μm mesh. Staining was performed using the following antibodies: NK1.1 (BioLegend), CD8a, CD4, F4/80 and CD11b (eBioscience), CD45, Gr1/Ly6C and Ly6G (BD Biosciences). Before analysis, count beads (Allophycocyanine calibrite beads, BD Biosciences) were added to calculate cell numbers. Flow cytometry was carried out using a FACS Canto II (BD Biosciences). Flow cytometric data are given as percentage of cells related to the number of CD45^+^ cells. Data were analysed with the FlowJo software (TreeStar Inc.).

### Cell culture

L929 (ATCC CCL-1) cells were cultured in DMEM supplemented with 10% fetal calf serum (FCS), penicillin (100 IU ml^−1^), streptomycin (0.1 mg ml^−1^) and L-glutamine (0.03%). 3T3-L1 CARΔ1 cells^64^ were maintained at a non-differentiated state in DMEM supplemented with 10% bovine calf serum, and the medium was changed every 2 days without ever reaching confluence. Adipocyte conversion was induced by treating 2-day post-confluent cultures with DMEM supplemented with 10% FCS and dexamethasone, isobutylmethylxanthine and insulin according to the manufacturer's specifications (Differentiation Kit DIF001 Sigma). Cells were treated by zVAD (Merck Millipore—20 μM), Nec-1s (Santa Cruz—10 μM) and GSK-872 (Merck Millipore—3 μM).

For isolation of macrophages, BM cells were isolated from the femur and tibia of 16-week-old C57BL6 and RIPK3^−/−^ mice. To obtain fibroblast-conditioned medium, which is known to contain the macrophage colony-stimulating factor (CSF1), L929 fibroblasts were cultured in RPMI medium containing 10% FCS for 3 days, and the supernatant was collected, filtered and stored until usage at −80 °C. BM cells were cultured in RPMI medium containing 10% FCS and 20% fibroblast-conditioned medium for 1 week on bacterial grade plastic plates (Greiner). On day 7, cells were left untreated, stimulated for additional 16 h with TNF (20 ng ml^−1^) or placed with medium containing adipocyte debris or cleared from them. All *in vitro* experiments are representative of three independent experiments. All cell lines were tested and were free of mycoplasma infection.

### Generation of a recombinant adenovirus encoding RIPK3

Cloning of mouse full-length RIP3 (NCBI Reference Sequence: NM_019955.2) into a pDonR201 gateway vector (Invitrogen) was carried out by PCR. Generation of an adenovirus encoding full-length RIP3 cDNA was carried out using the ViraPower Adenoviral Expression System (Invitrogen) according to the manufacturer's protocol. A lacZ encoding adenovirus served as control.

### Statistical analysis and general experimental design

We calculated sample size using size power analysis methods (GraphPad StatMate) for *a priori* determination based on the s.d. of previous experiments in WT mice. We calculated the minimal sample size for each group as seven animals. For some experiments, fewer animals were found sufficient to obtain statistical differences. In the serum analyses, at some time points all results from different longitudinally followed groups of mice were combined. Animals with same sex and same age were employed to minimize physiological variability and to reduce s.d. from the mean. The exclusion criteria for animals were established in consultation with a veterinarian and on the basis of experimental outcomes. In case of death or sickness, the animal was excluded from analysis. Tissue samples were excluded in cases of failure in extraction of RNA or protein of suitable quality and quantity. Animal experiments were blinded in terms of genetic background of mice by using ear number codes during the CD-HFD-feeding periods. Statistical tests were used as described in the Figure legends. Statistical analyses were performed using the GraphPad Prism software (version 5.0). All data are presented as mean±s.e.m. and were analysed by analysis of variance (ANOVA) with Bonferroni's *post hoc* multiple comparison test. Analysis of two groups of samples was performed using Student's *t*-test. Correlations were assessed by non-parametric Spearman's test. Statistical significance was indicated as follows: ***P<0.001; **P<0.01; *P<0.05; n.s., not significant.

### Data availability

The authors declare that the data supporting the findings of this study are available within the article and its Supplementary Information files.

## Additional information

**How to cite this article:** Gautheron, J. *et al*. The necroptosis-inducing kinase RIPK3 dampens adipose tissue inflammation and glucose intolerance. *Nat. Commun.* 7:11869 doi: 10.1038/ncomms11869 (2016).

## Supplementary Material

Supplementary Information Supplementary Figure 1-11 and Supplementary Tables 1-2

Supplementary Movie 1 L929 cells were incubated with TNF and activation of cell death was monitored for 8 hours.

Supplementary Movie 2 L929 cells were incubated with zVAD 1 hour prior to stimulation with TNF and activation of cell death was monitored for 8 hours.

## References

[b1] Hameed I. . Type 2 diabetes mellitus: from a metabolic disorder to an inflammatory condition. World J. Diabetes 6, 598–612 (2015). 25987957 10.4239/wjd.v6.i4.598 PMC4434080

[b2] Kahn S. E., Cooper M. E. & Del Prato S. Pathophysiology and treatment of type 2 diabetes: perspectives on the past, present, and future. Lancet 383, 1068–1083 (2014). 24315620 10.1016/S0140-6736(13)62154-6 PMC4226760

[b3] Scherer P. E. Adipose tissue: from lipid storage compartment to endocrine organ. Diabetes 55, 1537–1545 (2006). 16731815 10.2337/db06-0263

[b4] Capurso C. & Capurso A. From excess adiposity to insulin resistance: the role of free fatty acids. Vasc. Pharmacol. 57, 91–97 (2012). 10.1016/j.vph.2012.05.003 22609131

[b5] Feng D. . High-fat diet-induced adipocyte cell death occurs through a cyclophilin D intrinsic signaling pathway independent of adipose tissue inflammation. Diabetes 60, 2134–2143 (2011). 21734017 10.2337/db10-1411 PMC3142076

[b6] Musi N. & Goodyear L. J. Insulin resistance and improvements in signal transduction. Endocrine 29, 73–80 (2006). 16622294 10.1385/ENDO:29:1:73

[b7] Murano I. . Dead adipocytes, detected as crown-like structures, are prevalent in visceral fat depots of genetically obese mice. J. Lipid Res. 49, 1562–1568 (2008). 18390487 10.1194/jlr.M800019-JLR200

[b8] Cinti S. . Adipocyte death defines macrophage localization and function in adipose tissue of obese mice and humans. J. Lipid Res. 46, 2347–2355 (2005). 16150820 10.1194/jlr.M500294-JLR200

[b9] Alkhouri N. . Adipocyte apoptosis, a link between obesity, insulin resistance, and hepatic steatosis. J. Biol. Chem. 285, 3428–3438 (2010). 19940134 10.1074/jbc.M109.074252 PMC2823448

[b10] Silke J., Rickard J. A. & Gerlic M. The diverse role of RIP kinases in necroptosis and inflammation. Nat. Immunol. 16, 689–697 (2015). 26086143 10.1038/ni.3206

[b11] Ashkenazi A. & Salvesen G. Regulated cell death: signaling and mechanisms. Annu. Rev. Cell Dev. Biol. 30, 337–356 (2014). 25150011 10.1146/annurev-cellbio-100913-013226

[b12] Sun L. . Mixed lineage kinase domain-like protein mediates necrosis signaling downstream of RIP3 kinase. Cell 148, 213–227 (2012). 22265413 10.1016/j.cell.2011.11.031

[b13] Newton K. RIPK1 and RIPK3: critical regulators of inflammation and cell death. Trends Cell Biol. 25, 347–353 (2015). 25662614 10.1016/j.tcb.2015.01.001

[b14] Gautheron J. . A positive feedback loop between RIP3 and JNK controls non-alcoholic steatohepatitis. EMBO Mol. Med. 6, 1062–1074 (2014). 24963148 10.15252/emmm.201403856 PMC4154133

[b15] Roychowdhury S., McMullen M. R., Pisano S. G., Liu X. & Nagy L. E. Absence of receptor interacting protein kinase 3 prevents ethanol-induced liver injury. Hepatology 57, 1773–1783 (2013). 23319235 10.1002/hep.26200 PMC3628968

[b16] Lin J. . A role of RIP3-mediated macrophage necrosis in atherosclerosis development. Cell Rep. 3, 200–210 (2013). 23333278 10.1016/j.celrep.2012.12.012

[b17] Vitner E. B. . RIPK3 as a potential therapeutic target for Gaucher's disease. Nat. Med. 20, 204–208 (2014). 24441827 10.1038/nm.3449

[b18] Linkermann A. . Two independent pathways of regulated necrosis mediate ischemia-reperfusion injury. Proc. Natl Acad. Sci. USA 110, 12024–12029 (2013). 23818611 10.1073/pnas.1305538110 PMC3718149

[b19] Luedde M. . RIP3, a kinase promoting necroptotic cell death, mediates adverse remodelling after myocardial infarction. Cardiovasc. Res. 103, 206–216 (2014). 24920296 10.1093/cvr/cvu146

[b20] Newton K., Sun X. & Dixit V. M. Kinase RIP3 is dispensable for normal NF-kappa Bs, signaling by the B-cell and T-cell receptors, tumor necrosis factor receptor 1, and Toll-like receptors 2 and 4. Mol. Cell Biol. 24, 1464–1469 (2004). 14749364 10.1128/MCB.24.4.1464-1469.2004 PMC344190

[b21] Wolf M. J. . Metabolic activation of intrahepatic CD8+ T cells and NKT cells causes nonalcoholic steatohepatitis and liver cancer via cross-talk with hepatocytes. Cancer Cell 26, 549–564 (2014). 25314080 10.1016/j.ccell.2014.09.003

[b22] Raubenheimer P. J., Nyirenda M. J. & Walker B. R. A choline-deficient diet exacerbates fatty liver but attenuates insulin resistance and glucose intolerance in mice fed a high-fat diet. Diabetes 55, 2015–2020 (2006). 16804070 10.2337/db06-0097

[b23] Linkermann A. . Synchronized renal tubular cell death involves ferroptosis. Proc. Natl Acad. Sci. USA 111, 16836–16841 (2014). 25385600 10.1073/pnas.1415518111 PMC4250130

[b24] Lin Y. & Sun Z. Current views on type 2 diabetes. J. Endocrinol. 204, 1–11 (2010). 19770178 10.1677/JOE-09-0260 PMC2814170

[b25] Shao J., Yamashita H., Qiao L. & Friedman J. E. Decreased Akt kinase activity and insulin resistance in C57BL/KsJ-Leprdb/db mice. J. Endocrinol. 167, 107–115 (2000). 11018758 10.1677/joe.0.1670107

[b26] Nathan D. M. . Impaired fasting glucose and impaired glucose tolerance: implications for care. Diabetes Care 30, 753–759 (2007). 17327355 10.2337/dc07-9920

[b27] Wensveen F. M. . NK cells link obesity-induced adipose stress to inflammation and insulin resistance. Nat. Immunol. 16, 376–385 (2015). 25729921 10.1038/ni.3120

[b28] Suganami T. & Ogawa Y. Adipose tissue macrophages: their role in adipose tissue remodeling. J. Leukoc. Biol. 88, 33–39 (2010). 20360405 10.1189/jlb.0210072

[b29] Grant R. W. & Stephens J. M. Fat in flames: influence of cytokines and pattern recognition receptors on adipocyte lipolysis. Am. J. Physiol. Endocrinol. Metab. 309, E205–E213 (2015). 26058863 10.1152/ajpendo.00053.2015

[b30] Herrero L., Shapiro H., Nayer A., Lee J. & Shoelson S. E. Inflammation and adipose tissue macrophages in lipodystrophic mice. Proc. Natl Acad. Sci. USA 107, 240–245 (2010). 20007767 10.1073/pnas.0905310107 PMC2806777

[b31] Oberst A. . Catalytic activity of the caspase-8-FLIP(L) complex inhibits RIPK3-dependent necrosis. Nature 471, 363–367 (2011). 21368763 10.1038/nature09852 PMC3077893

[b32] Kaiser W. J. . RIP3 mediates the embryonic lethality of caspase-8-deficient mice. Nature 471, 368–372 (2011). 21368762 10.1038/nature09857 PMC3060292

[b33] Newton K. . Activity of protein kinase RIPK3 determines whether cells die by necroptosis or apoptosis. Science 343, 1357–1360 (2014). 24557836 10.1126/science.1249361

[b34] Green H. & Meuth M. An established pre-adipose cell line and its differentiation in culture. Cell 3, 127–133 (1974). 4426090 10.1016/0092-8674(74)90116-0

[b35] Vercammen D. . Inhibition of caspases increases the sensitivity of L929 cells to necrosis mediated by tumor necrosis factor. J. Exp. Med. 187, 1477–1485 (1998). 9565639 10.1084/jem.187.9.1477 PMC2212268

[b36] Wang H. . Mixed lineage kinase domain-like protein MLKL causes necrotic membrane disruption upon phosphorylation by RIP3. Mol. Cell 54, 133–146 (2014). 24703947 10.1016/j.molcel.2014.03.003

[b37] Pasparakis M. & Vandenabeele P. Necroptosis and its role in inflammation. Nature 517, 311–320 (2015). 25592536 10.1038/nature14191

[b38] Moriwaki K. & Chan F. K. Necrosis-dependent and independent signaling of the RIP kinases in inflammation. Cytokine Growth Factor Rev. 25, 167–174 (2014). 24412261 10.1016/j.cytogfr.2013.12.013 PMC3999177

[b39] Kearney C. J. . Necroptosis suppresses inflammation via termination of TNF- or LPS-induced cytokine and chemokine production. Cell Death Differ. 22, 1313–1327 (2015). 25613374 10.1038/cdd.2014.222 PMC4495357

[b40] Wang Q., Ju X., Zhou Y. & Chen K. Necroptotic cells release find-me signal and are engulfed without proinflammatory cytokine production. In vitro cellular & developmental biology Animal 51, 1033–1039 (2015). 26091630 10.1007/s11626-015-9926-7

[b41] Davidovich P., Kearney C. J. & Martin S. J. Inflammatory outcomes of apoptosis, necrosis and necroptosis. Biological chemistry 395, 1163–1171 (2014). 25153241 10.1515/hsz-2014-0164

[b42] Vucur M. . RIP3 inhibits inflammatory hepatocarcinogenesis but promotes cholestasis by controlling caspase-8- and JNK-dependent compensatory cell proliferation. Cell reports 4, 776–790 (2013). 23972991 10.1016/j.celrep.2013.07.035

[b43] Kelliher M. A. . The death domain kinase RIP mediates the TNF-induced NF-kappaB signal. Immunity 8, 297–303 (1998). 9529147 10.1016/s1074-7613(00)80535-x

[b44] Festjens N., Vanden Berghe T., Cornelis S. & P. Vandenabeele RIP1 a kinase on the crossroads of a cell's decision to live or die. Cell Death Differ. 14, 400–410 (2007). 17301840 10.1038/sj.cdd.4402085

[b45] Wronska A. . White adipose tissue depot-specific activity of lipogenic enzymes in response to fasting and refeeding in young and old rats. Gerontology 61, 448–455 (2015). 25721559 10.1159/000371578

[b46] Seimon T. & Tabas I. Mechanisms and consequences of macrophage apoptosis in atherosclerosis. J. Lipid Res. 50, (Suppl): S382–S387 (2009). 18953058 10.1194/jlr.R800032-JLR200 PMC2674693

[b47] Angelovich T. A., Hearps A. C. & Jaworowski A. Inflammation-induced foam cell formation in chronic inflammatory disease. Immunol. Cell Biol. 93, 683–693 (2015). 25753272 10.1038/icb.2015.26

[b48] Fischer-Posovszky P., Wang Q. A., Asterholm I. W., Rutkowski J. M. & Scherer P. E. Targeted deletion of adipocytes by apoptosis leads to adipose tissue recruitment of alternatively activated M2 macrophages. Endocrinology 152, 3074–3081 (2011). 21693678 10.1210/en.2011-1031 PMC3138241

[b49] Zhang Y. & Huang C. Targeting adipocyte apoptosis: a novel strategy for obesity therapy. Biochem. Biophys. Res. Commun. 417, 1–4 (2012). 22172945 10.1016/j.bbrc.2011.11.158

[b50] Kern P. A. . The expression of tumor necrosis factor in human adipose tissue. Regulation by obesity, weight loss, and relationship to lipoprotein lipase. J. Clin. Invest. 95, 2111–2119 (1995). 7738178 10.1172/JCI117899 PMC295809

[b51] Yki-Jarvinen H. Non-alcoholic fatty liver disease as a cause and a consequence of metabolic syndrome. Lancet Diabetes Endocrinol. 2, 901–910 (2014). 24731669 10.1016/S2213-8587(14)70032-4

[b52] Dyson J. . Hepatocellular cancer: the impact of obesity, type 2 diabetes and a multidisciplinary team. J. Hepatol. 60, 110–117 (2014). 23978719 10.1016/j.jhep.2013.08.011

[b53] Afonso M. B. . Necroptosis is a key pathogenic event in human and experimental murine models of non-alcoholic steatohepatitis. Clin. Sci. 129, 721–739 (2015). 26201023 10.1042/CS20140732

[b54] Gautheron J., Vucur M. & Luedde T. Necroptosis in nonalcoholic steatohepatitis. Cell. Mol. Gastroenterol. Hepatol. 1, 264–265 (2015). 10.1016/j.jcmgh.2015.02.001 PMC5301189 28210679

[b55] Ratziu V. . A phase 2, randomized, double-blind, placebo-controlled study of GS-9450 in subjects with nonalcoholic steatohepatitis. Hepatology 55, 419–428 (2012). 22006541 10.1002/hep.24747 PMC3779694

[b56] Kloting N. . Insulin-sensitive obesity. Am. J. Physiol. Endocrinol. Metab. 299, E506–E515 (2010). 20570822 10.1152/ajpendo.00586.2009

[b57] Caporaso J. G. . Ultra-high-throughput microbial community analysis on the Illumina HiSeq and MiSeq platforms. ISME J. 6, 1621–1624 (2012). 22402401 10.1038/ismej.2012.8 PMC3400413

[b58] Caporaso J. G. . QIIME allows analysis of high-throughput community sequencing data. Nat. Methods 7, 335–336 (2010). 20383131 10.1038/nmeth.f.303 PMC3156573

[b59] McDonald D. . An improved Greengenes taxonomy with explicit ranks for ecological and evolutionary analyses of bacteria and archaea. ISME J. 6, 610–618 (2012). 22134646 10.1038/ismej.2011.139 PMC3280142

[b60] Ehling J. . Micro-CT imaging of tumor angiogenesis: quantitative measures describing micromorphology and vascularization. Am. J. Pathol. 184, 431–441 (2014). 24262753 10.1016/j.ajpath.2013.10.014 PMC3920056

[b61] Gremse F. . Absorption reconstruction improves biodistribution assessment of fluorescent nanoprobes using hybrid fluorescence-mediated tomography. Theranostics 4, 960–971 (2014). 25157277 10.7150/thno.9293 PMC4142290

[b62] Gremse F. . Hybrid microCT-FMT imaging and image analysis. J. Vis. Exp. 100, e52770 (2015). 26066033 10.3791/52770 PMC4512251

[b63] Kleiner D. E. . Design and validation of a histological scoring system for nonalcoholic fatty liver disease. Hepatology 41, 1313–1321 (2005). 15915461 10.1002/hep.20701

[b64] Orlicky D. J., DeGregori J. & Schaack J. Construction of stable coxsackievirus and adenovirus receptor-expressing 3T3-L1 cells. J. Lipid Res. 42, 910–915 (2001). 11369798

