# Supplementary material for: The necroptosis-inducing kinase RIPK3 dampens adipose tissue inflammation and glucose intolerance
Source: Nat Commun. 2016 Jun 21;7:11869. doi: 10.1038/ncomms11869 (PMC4919522; doi:10.1038/ncomms11869)
Supplement: Supplementary Information — Supplementary Figure 1-11 and Supplementary Tables 1-2 [file ncomms11869-s1.pdf]

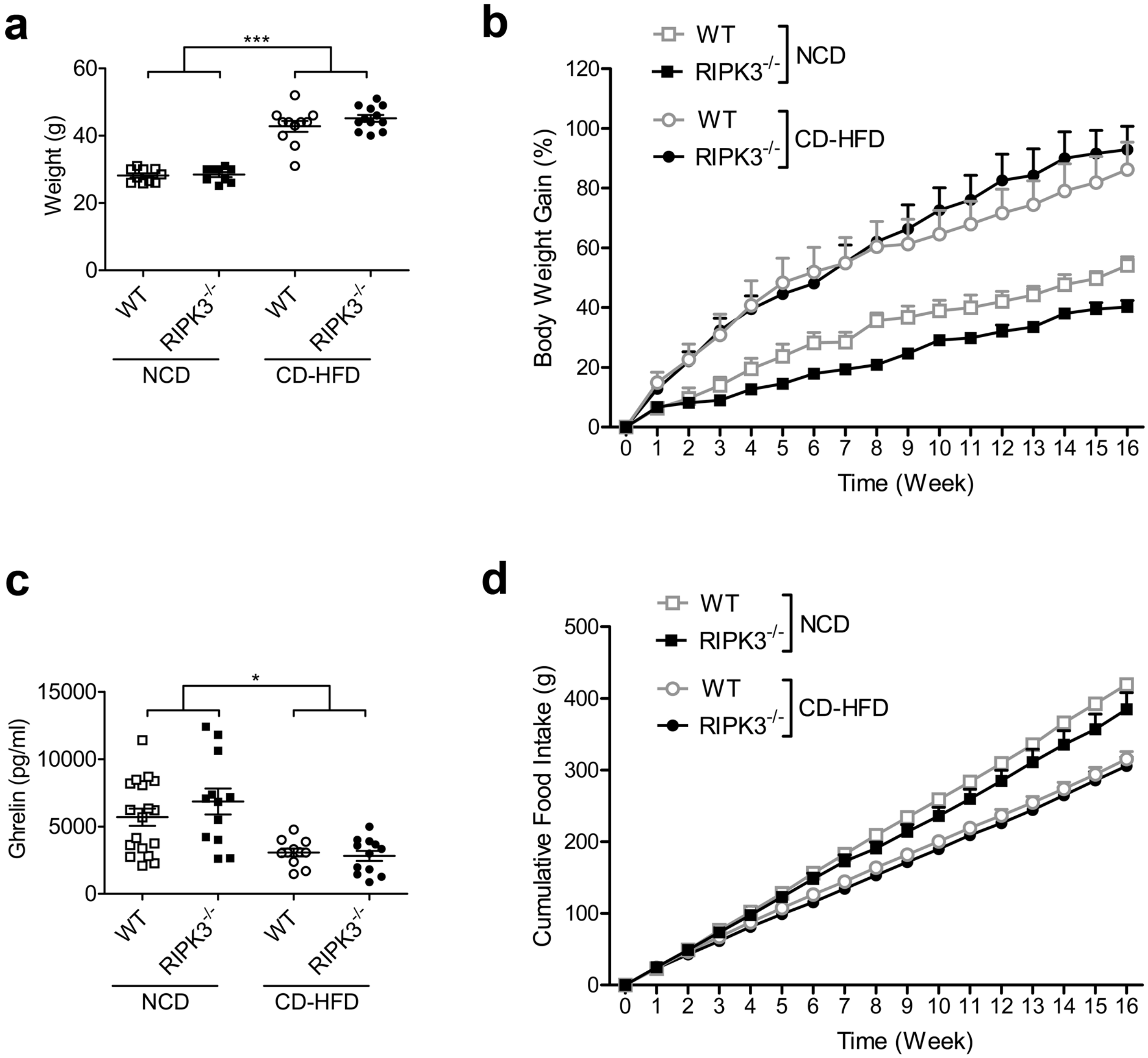

Supplementary Figure 1. Basic parameters of weight gain and food intake in WT and RIPK3$^{-/-}$ mice on normal chow diet (NCD) and choline-deficient high-fat diet (CD-HFD). (**a**) Absolute weight (*n* = 10 in each group) of WT and RIPK3$^{-/-}$ fed with CD-HFD or NCD for 16 weeks. *** *P* < 0.001. (**b**) Relative body weight gain [%] (*n* = 6 in each group) of WT and RIPK3$^{-/-}$ fed with CD-HFD or NCD for 16 weeks. (**c**) Fasting serum concentrations of ghrelin in NCD-fed WT (*n* = 18) and RIPK3$^{-/-}$ (*n* = 12) mice and in CD-HFD-fed WT (*n* = 11) and RIPK3$^{-/-}$ (*n* = 12) mice. * *P* < 0.05. (**d**) Cumulative food intake [g] (*n* = 6 in each group) of WT and RIPK3$^{-/-}$ fed with CD-HFD or NCD for 16 weeks. Differences between groups were determined by ANOVA with Bonferroni's post hoc test. All data are expressed as mean ± SEM.

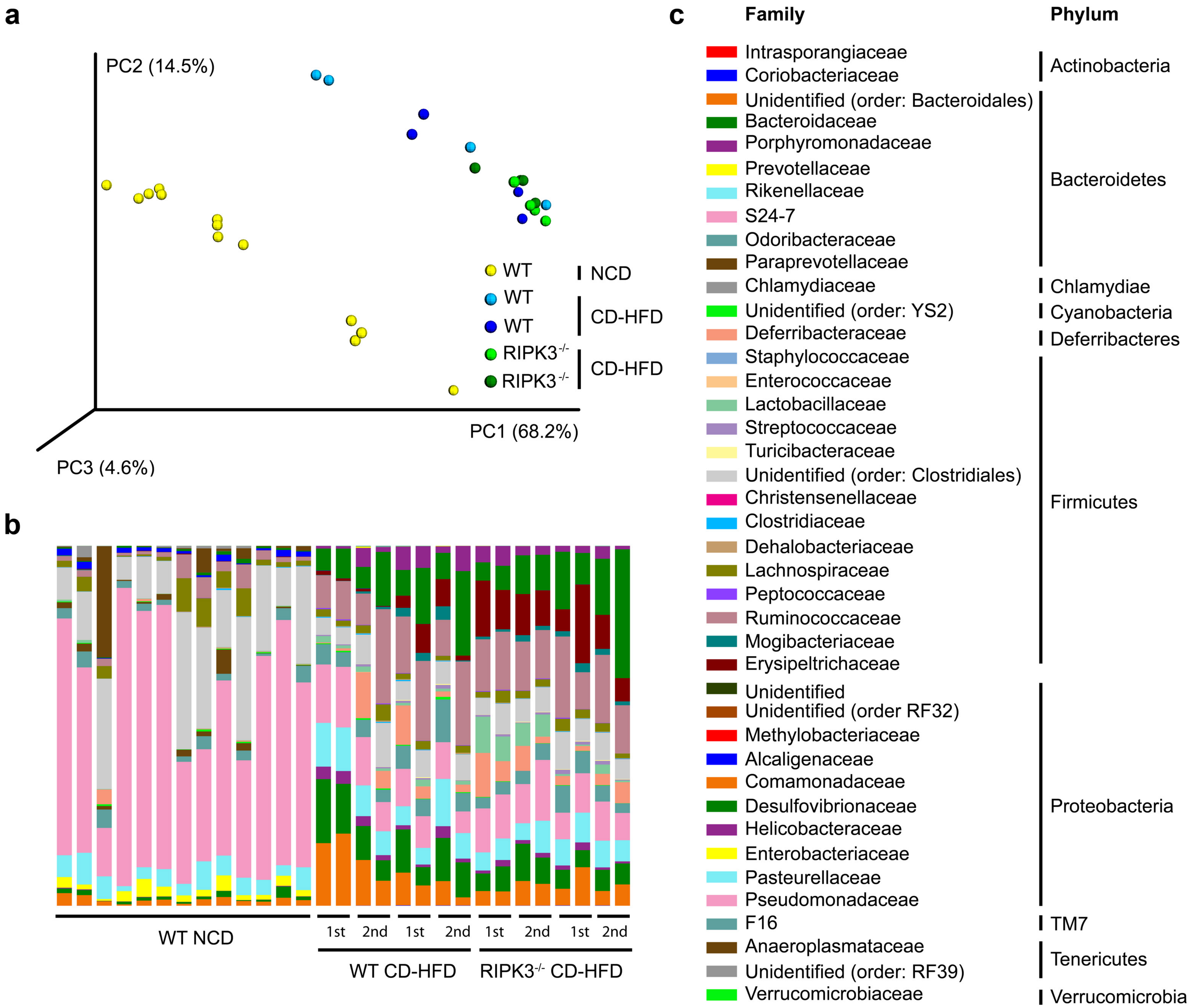

a
PC2 (14.5%)
PC1 (68.2%)
PC3 (4.6%)
WT
WT
WT
RIPK3-/-
RIPK3-/-
NCD
CD-HFD
CD-HFD
b
WT NCD
1st
2nd
1st
2nd
1st
2nd
1st
2nd
WT CD-HFD
RIPK3-/- CD-HFD
c
Family
Phylum
Intrasporangiaceae
Coriobacteriaceae
Unidentified (order: Bacteroidales)
Bacteroidaceae
Porphyromonadaceae
Prevotellaceae
Rikenellaceae
S24-7
Odoribacteraceae
Paraprevotellaceae
Chlamydiaceae
Unidentified (order: YS2)
Deferribacteraceae
Staphylococcaceae
Enterococcaceae
Lactobacillaceae
Streptococcaceae
Turicibacteraceae
Unidentified (order: Clostridiales)
Christensenellaceae
Clostridiaceae
Dehalobacteriaceae
Lachnospiraceae
Peptococcaceae
Ruminococcaceae
Mogibacteriaceae
Erysipeltrichaceae
Unidentified
Unidentified (order RF32)
Methylobacteriaceae
Alcaligenaceae
Comamonadaceae
Desulfovibrionaceae
Helicobacteraceae
Enterobacteriaceae
Pasteurellaceae
Pseudomonadaceae
F16
Anaeroplasmataceae
Unidentified (order: RF39)
Verrucomicrobiaceae
Actinobacteria
Bacteroidetes
Chlamydiae
Cyanobacteria
Deferribacteres
Firmicutes
Proteobacteria
TM7
Tenericutes
Verrucomicrobia

Supplementary Figure 2. Fecal microbiota analysis of WT and KO mice fed with CD-HFD. Bacterial 16S rDNA sequence-based analyses were performed on 29 fecal samples. Fresh fecal samples were collected from 4 different cages containing 4 animals each 1 month [1st] and 4 months [2nd] after starting CD-HFD. The WT control group contains 13 animals divided into 5 cages and kept under NCD. Cage inleds were exchanged between cages once a week. (**a**) Principal coordinates analysis (PCoA) based on weighted UniFrac metrics. Light and dark blue/green coloring represent 1st and 2nd sampling time point. (**b,c**) Relative abundance of bacteria classified at a family-level taxonomy.

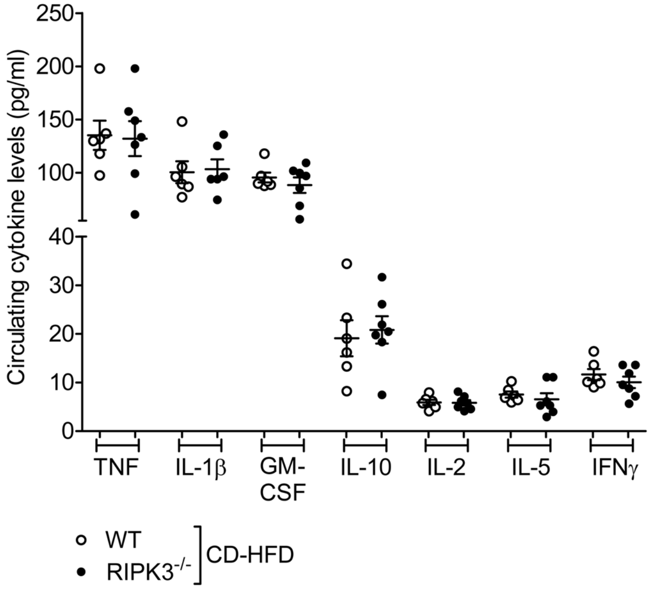

Supplementary Figure 3. Systemic inflammation induced by CD-HFD is unchanged between WT and KO mice. Fasting serum concentrations of TNF, IL-1β, GM-CSF, IL-10, IL-2, IL-5 and IFNγ in CD-HFD-fed WT (*n* = 6) and KO (*n* = 7) mice (16 weeks of CD-HFD feeding) were assessed by Bioplex Assay to evaluate the systemic inflammation. Differences between WT and KO mice were determined by Student *t* test. These data are shown as mean ± SEM.

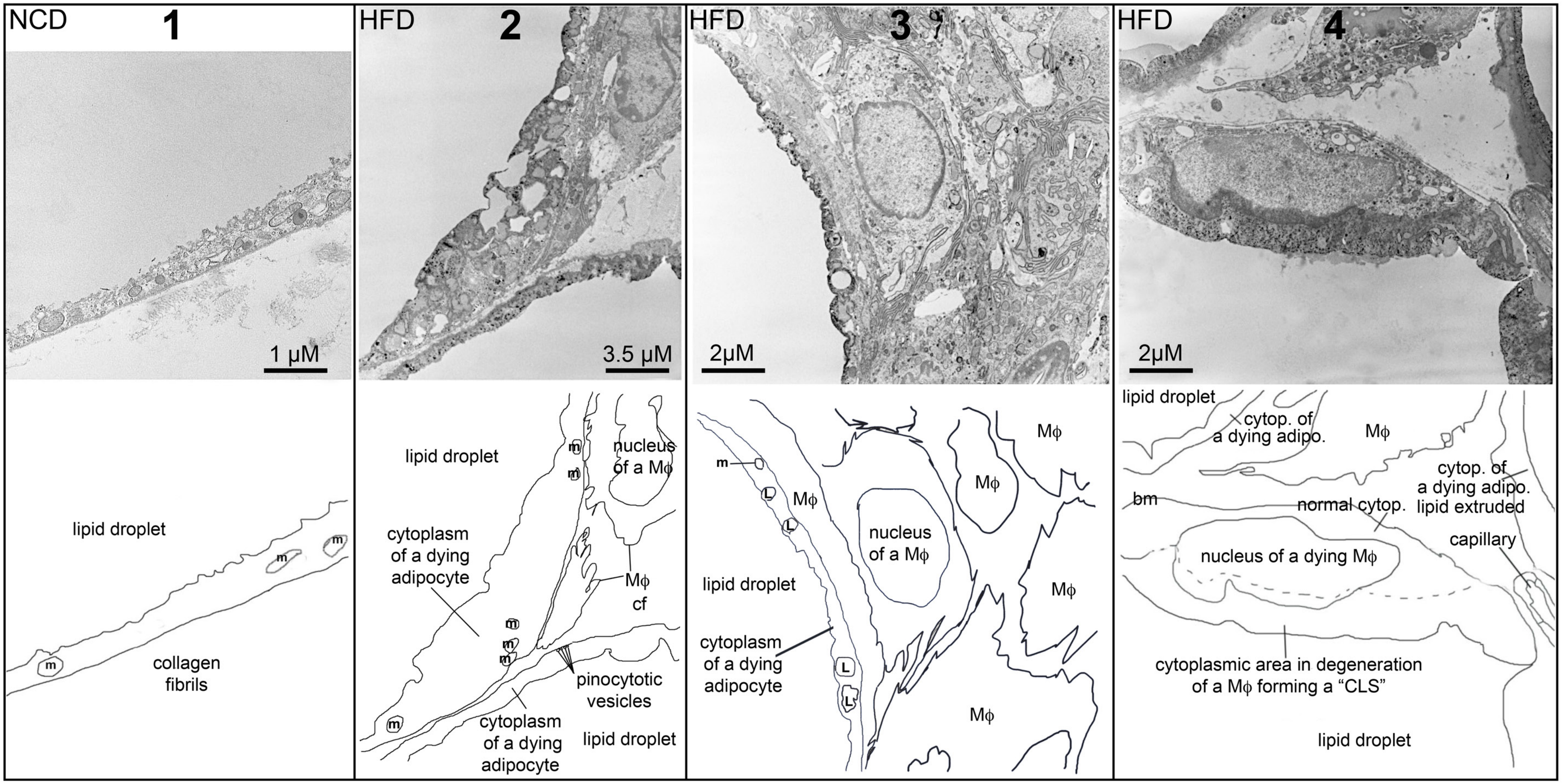

Supplementary Figure 4. Electron-microscopy analysis of adipocytes from obese WT mice revealed dying adipocytes surrounded by macrophages. 1: normal adipocyte. **2**: hypertrophic dying adipocytes at different stages of degeneration. In the upper left corner, some organelles such as mitochondria (m) are visible in adipocyte. In the bottom right, the cytoplasm of adipocyte is in advanced stage of degeneration and only few pinocytotic vesicles are visible. Two macrophages (MΦ) in close association with the two degenerating adipocytes are visible in the right part of the panel. cf: collagen fibrils. **3**: several macrophages (MΦ, right) are in close association with a degenerating adipocyte (left). L: small lipid droplets into the cytoplasm of the adipocyte. **4**: a macrophage with visually evident signs of degeneration inside the cytoplasm and the nucleus is visible. The degenerating macrophage is part of a crown-like structure, demonstrated by the absence of an adipocyte cytoplasm between the lipid droplet (remnants of dead adipocytes) and the macrophage. Furthermore, a distinct basal membrane (bm), owning to the dead adipocyte, is visible on the upper side (healthy side) of the macrophage.

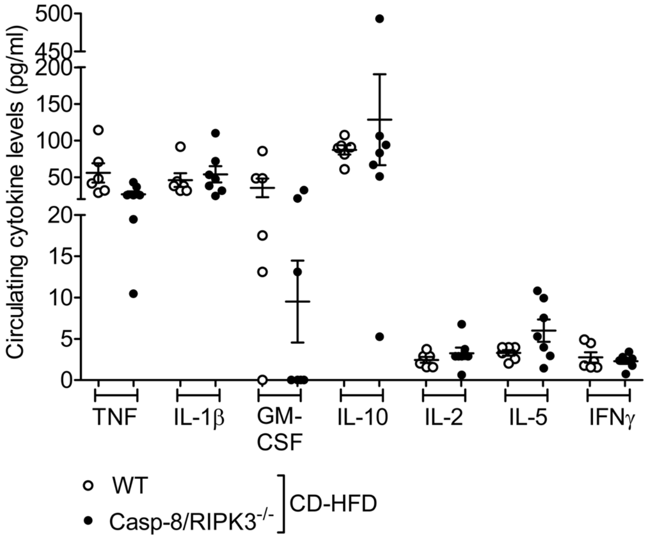

Supplementary Figure 5. Systemic inflammation induced by CD-HFD is unchanged between WT and DKO mice. Fasting serum concentrations of TNF, IL-1β, GM-CSF, IL-10, IL-2, IL-5 and IFNγ in CD-HFD-fed WT (n = 6) and Caspase-8/RIPK3$^{-/-}$ (n = 7) mice. Differences between WT and DKO mice were determined by Student t test. These data are expressed as mean ± SEM.

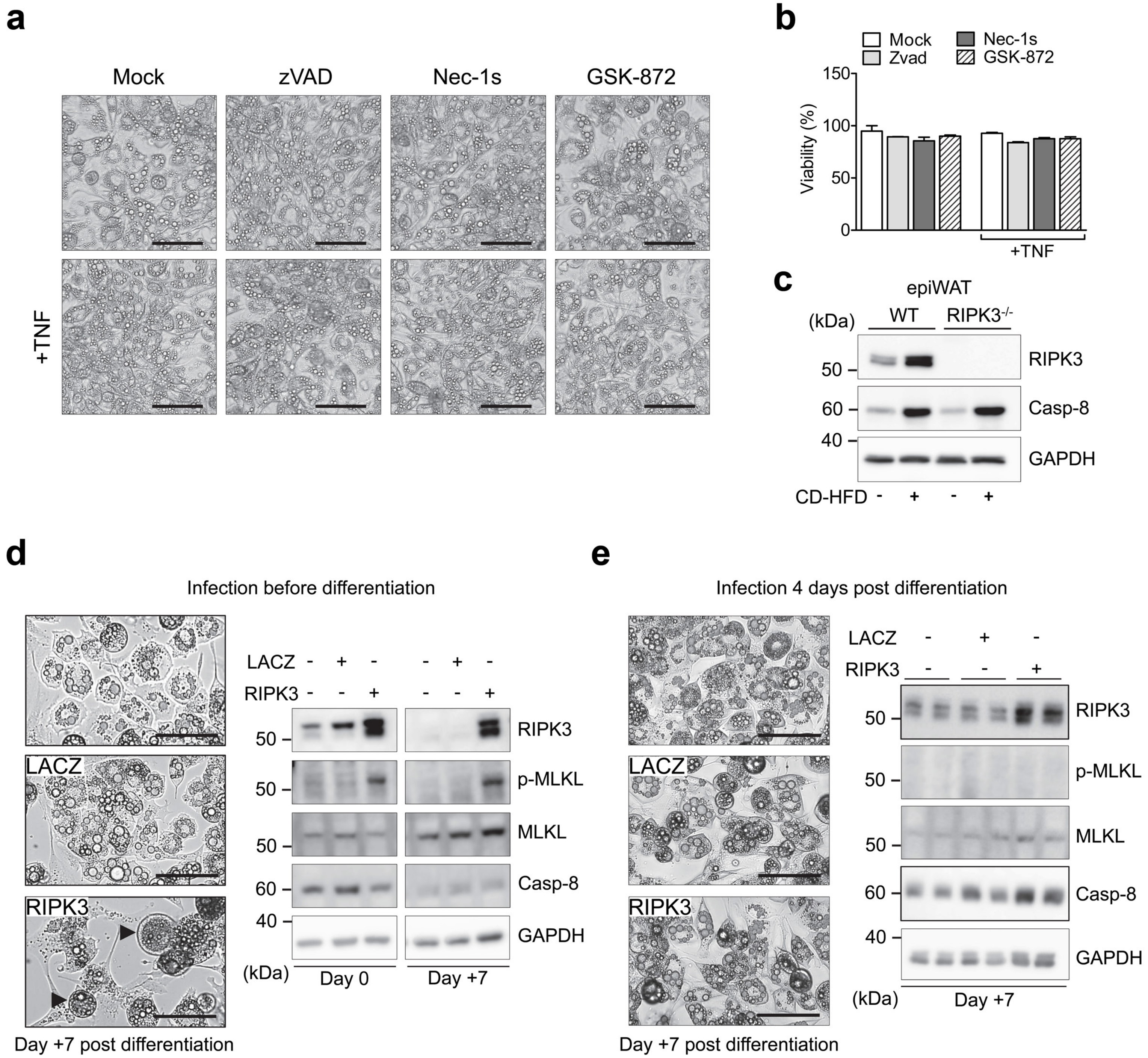

Supplementary Figure 6. RIPK3 overexpression does not trigger cell death in trans-differentiated 3T3-L1 cells. (**a**) Morphological features of transdifferentiated 3T3-L1 cells with an adipocyte phenoytpe that were treated for 19h with zVAD (inhibitor of caspases), Nec-1s (inhibitor of necroptosis) and GSK-872 (kinase inhibitor of RIPK3) in combination with or without TNF. (**b**) Representative cell survival analysis using a Cell Counting Kit-8 (CKK-8) assay after 19 hours of the indicated treatment. Error bars indicate SEM of duplicates. Scale bars, 100 µm. (**c**) Western blot analyses of RIPK3 and Caspase-8 in WT and RIPK3$^{-/-}$ mice fed with NCD or CD-HFD for 16 weeks. GAPDH is used as loading control. (**d,e**) 3T3-L1 fibroblasts were infected with adenovirus expressing RIPK3 or LACZ either 2 days before (**d**) or 4 days (**e**) after trans-differentiation. Western blots analyses for RIPK3, p-MLKL, MLKL, Caspase-8 and GAPDH were performed. Arrowheads indicate adipocytes with features of cells that underwent necroptosis.

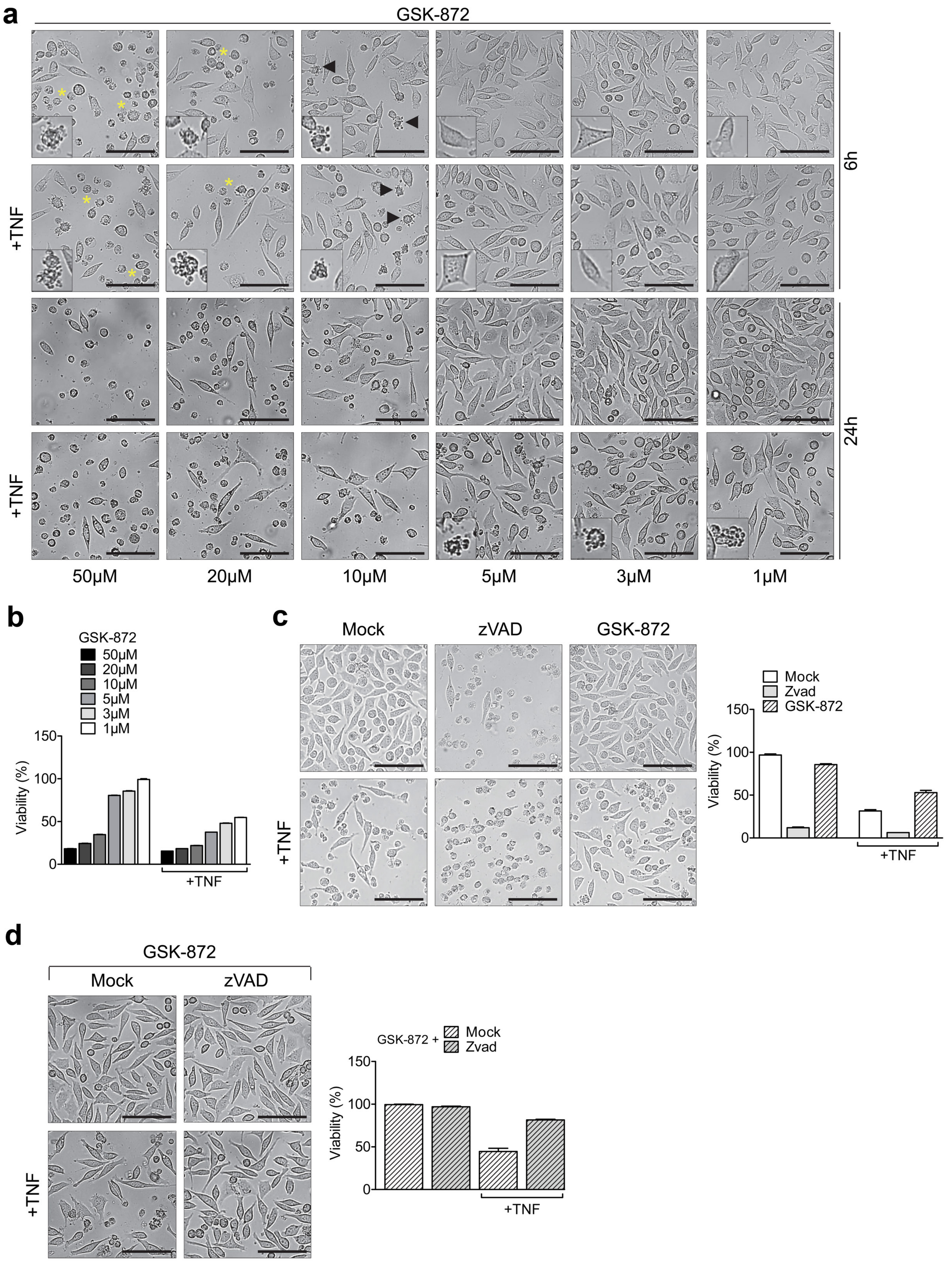

a
GSK-872
6h
24h
+TNF
+TNF
50μM
20μM
10μM
5μM
3μM
1μM
b
GSK-872
50μM
20μM
10μM
5μM
3μM
1μM
Viability (%)
150
100
50
0
+TNF
c
Mock
zVAD
GSK-872
+TNF
Mock
Zvad
GSK-872
Viability (%)
150
100
50
0
+TNF
d
GSK-872
Mock
zVAD
+TNF
GSK-872 +
Mock
Zvad
Viability (%)
150
100
50
0
+TNF

Supplementary Figure 7. Inhibition of RIPK3 kinase activity switches cell death mode from necroptosis to apoptosis upon TNF stimulation. (**a**) Morphological features of cultured L929 cells treated for 19h with GSK-872 (kinase inhibitor of RIPK3) in combination with or without TNF. Yellow asterisks designated groups of apoptotic cells and black arrowheads showed single apoptotic cells. Scale bars, 100 μm. (**b**) Cell survival analyses using a Cell Counting Kit-8 (CKK-8) assay after 24 hours of the indicated dose treatment of GSK-872. Data are representative of at least three independent experiments. (**c**) Morphological features (left panel) and statistical evaluation of survival (right panel) of L929 cells treated with zVAD (20μM) or GSK-872 (3μM) in combination with TNF (20ng/ml). Error bars indicate SEM of duplicates. Scale bars, 100 μm. (**d**) The induction of apoptosis by GSK-872 could be reverted by additional usage of zVAD.

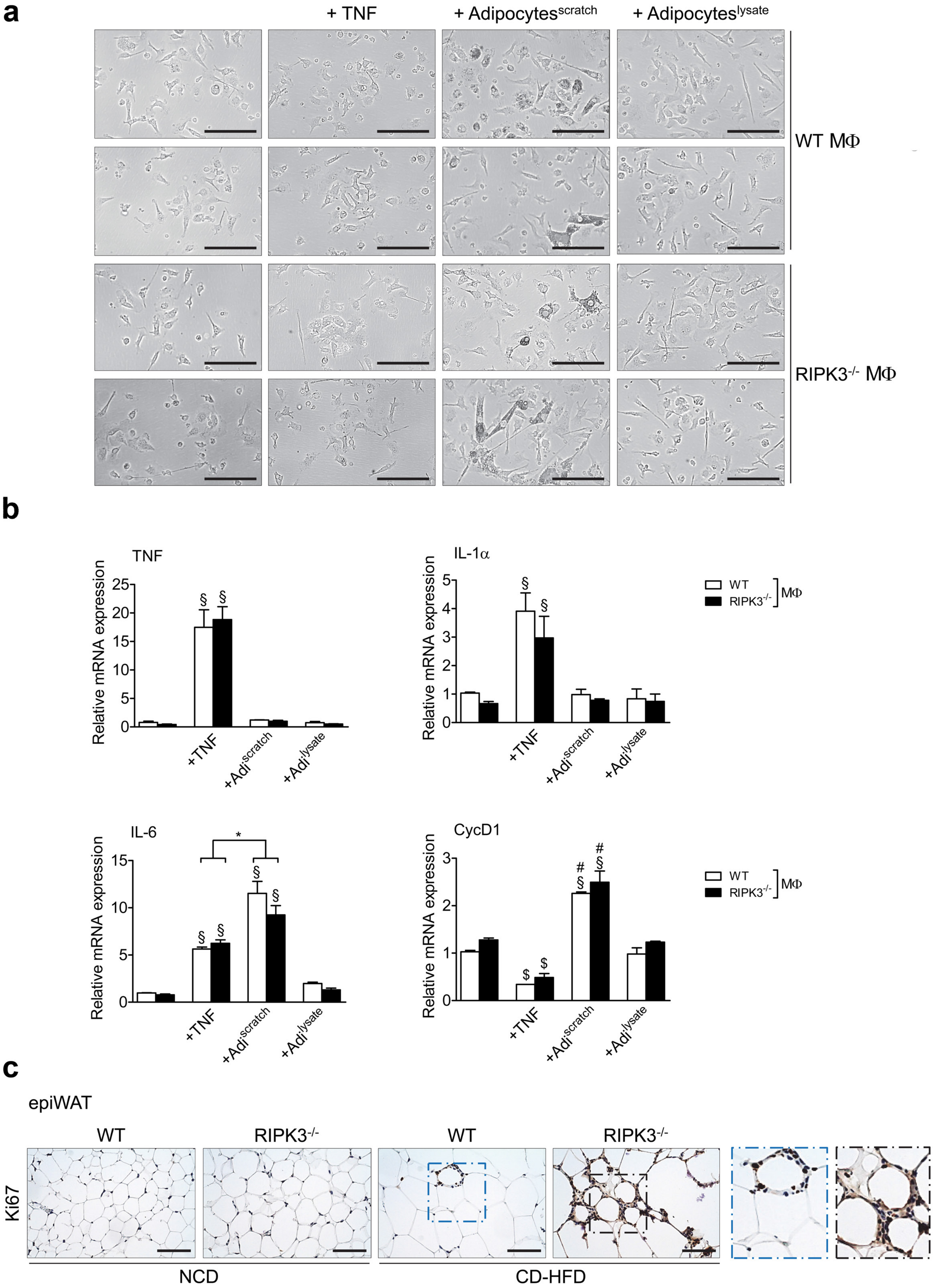

a
+ TNF
+ Adipocytes scratch
+ Adipocytes lysate
WT MΦ
RIPK3-/- MΦ
b
TNF
IL-1α
IL-6
CycD1
Relative mRNA expression
+TNF
+Adi scratch
+Adi lysate
WT
RIPK3-/-
MΦ
c
epiWAT
WT
RIPK3-/-
Ki67
NCD
CD-HFD

Supplementary Figure 8. WT and RIPK3$^{-/-}$ macrophages are similarly activated in response to TNF and adipocyte debris. (**a**) Morphological features of bone marrow derived macrophages isolated from WT and RIPK3$^{-/-}$ mice. WT and KO macrophages were stimulated with TNF and put in presence of scratched cultured 3T3-L1 adipocytes containing cell debris and few alive adipocytes (Adipocytes^{scratch}) or cleared from cell debris (Adipocytes^{lysate}). (**b**) TNF, IL-1α, IL-6 and CycD1 mRNA levels were assessed by RT-PCR in the different conditions seen in (a). Values were calculated relative to untreated WT macrophages and β-actin was used as an internal standard, n = 4 per group. Differences between the groups were determined by ANOVA with Bonferroni's *post hoc* test. Error bars represent SEM. § indicates that mRNA levels are significantly increased from untreated macrophages. # indicates that mRNA levels were significantly increased compared to all others groups. Cell debris from adipocytes activated proliferation of macrophages and expression of IL-6. (**c**) Representative images of immunohistochemical stainings for Ki67$^+$ cells in epiWAT of WT and KO mice fed 16 weeks with NCD and CD-HFD. Scale bars, 100 µm.

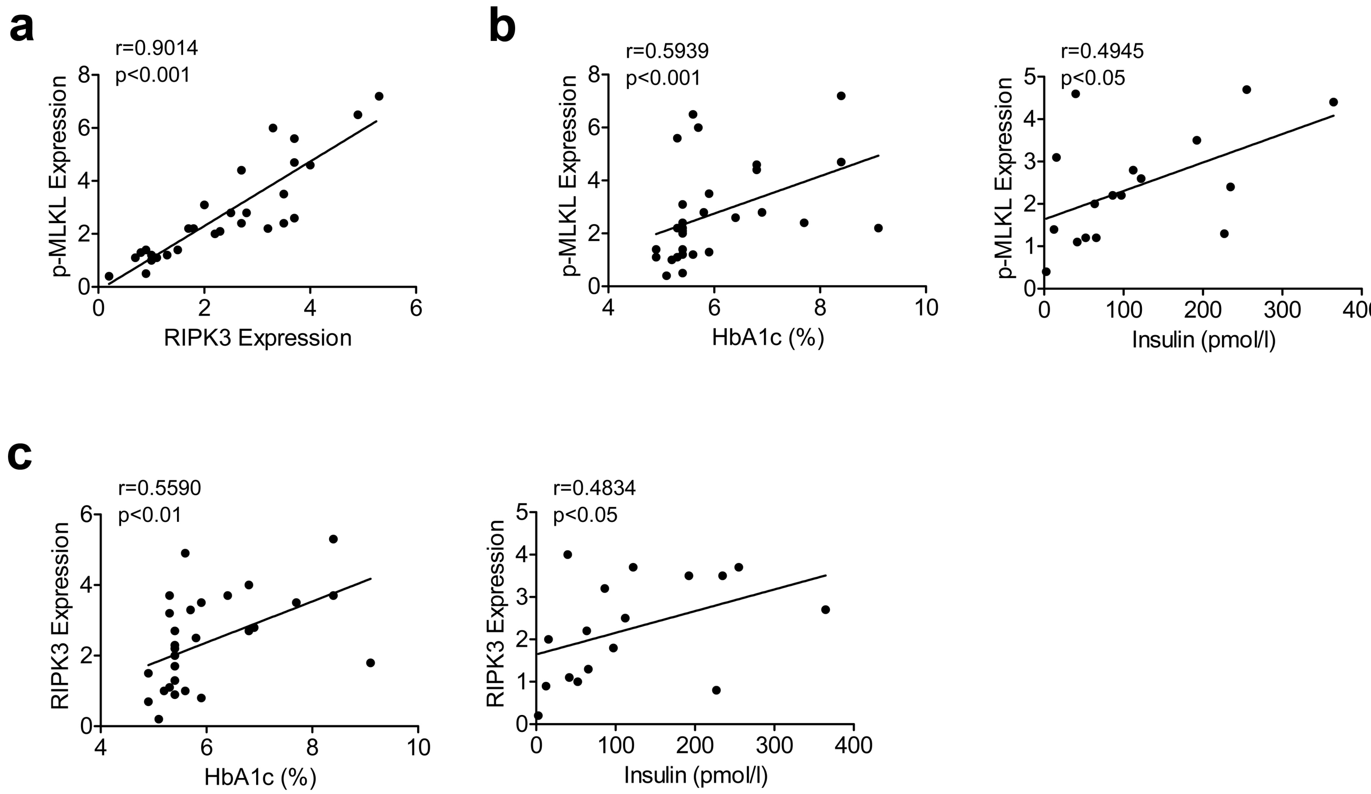

Supplementary Figure 9. RIPK3 is overexpressed in the visceral white adipose tissue (visWAT) of obese and diabetic human patients and correlates with metabolic serum markers. Data were obtained from lean controls, obese non-diabetic patients and obese patients with type 2-diabetes (T2D). Correlations were assessed using the non-parametric Spearman's test. (**a**) Correlation between p-MLKL and RIPK3 expression levels in the visWAT of all patients (n = 30). *** P < 0.001. (**b**) Expression levels of p-MLKL in visceral white adipose tissue of lean, non-diabetic obese as well as diabetic and obese patients were correlated with circulating serum levels of HbA1c, insulin (n = 30). * P < 0.05, *** P < 0.001. (**c**) Expression levels of RIPK3 in visceral white adipose tissue of lean, non-diabetic obese as well as diabetic and obese patients were correlated with circulating serum levels of HbA1c, insulin (n = 30). * P < 0.05, ** P < 0.01.

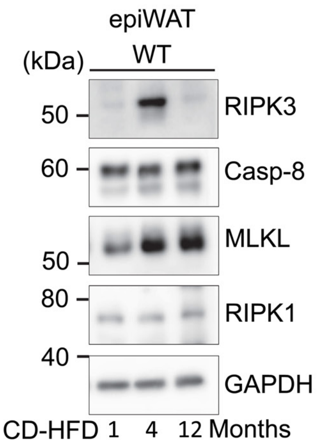

Supplementary Figure 10. Upregulation of RIPK3, MLKL and Caspase-8 in WAT after CD-HFD feeding. Western blots analyses in epiWAT of WT mice fed 1, 4 and 12 months with CD-HFD. RIPK3, Caspase-8, MLKL, RIPK1 are analyzed together with GAPDH as a loading control.

Supplementary Figure 11. Unedited scans for all Western blotting images. Black arrow and dashed line box show the lanes of the unedited gels correspond to those shown in the cropped images used in figures.

Full unedited gels for figure 2e | Skeletal muscle

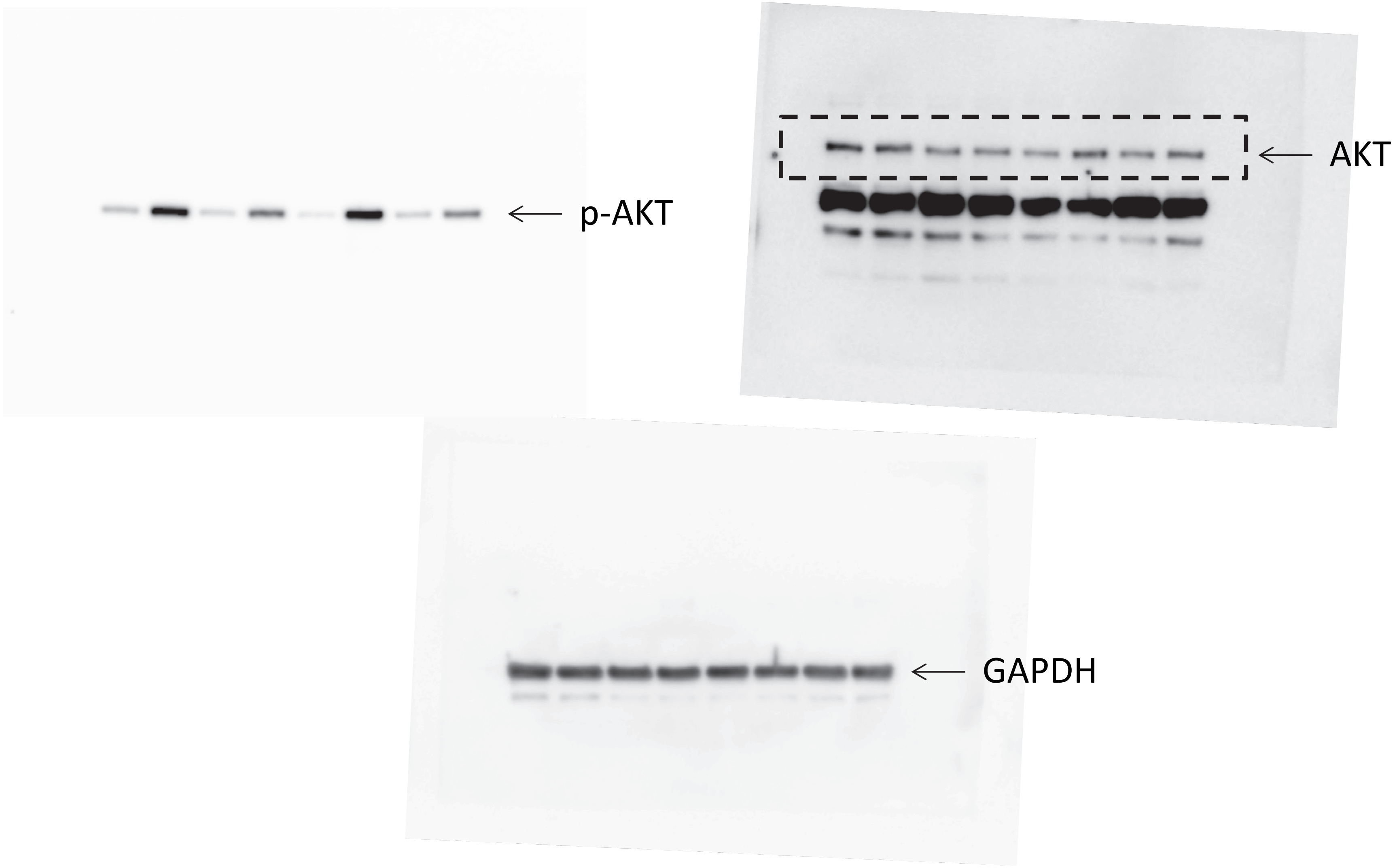

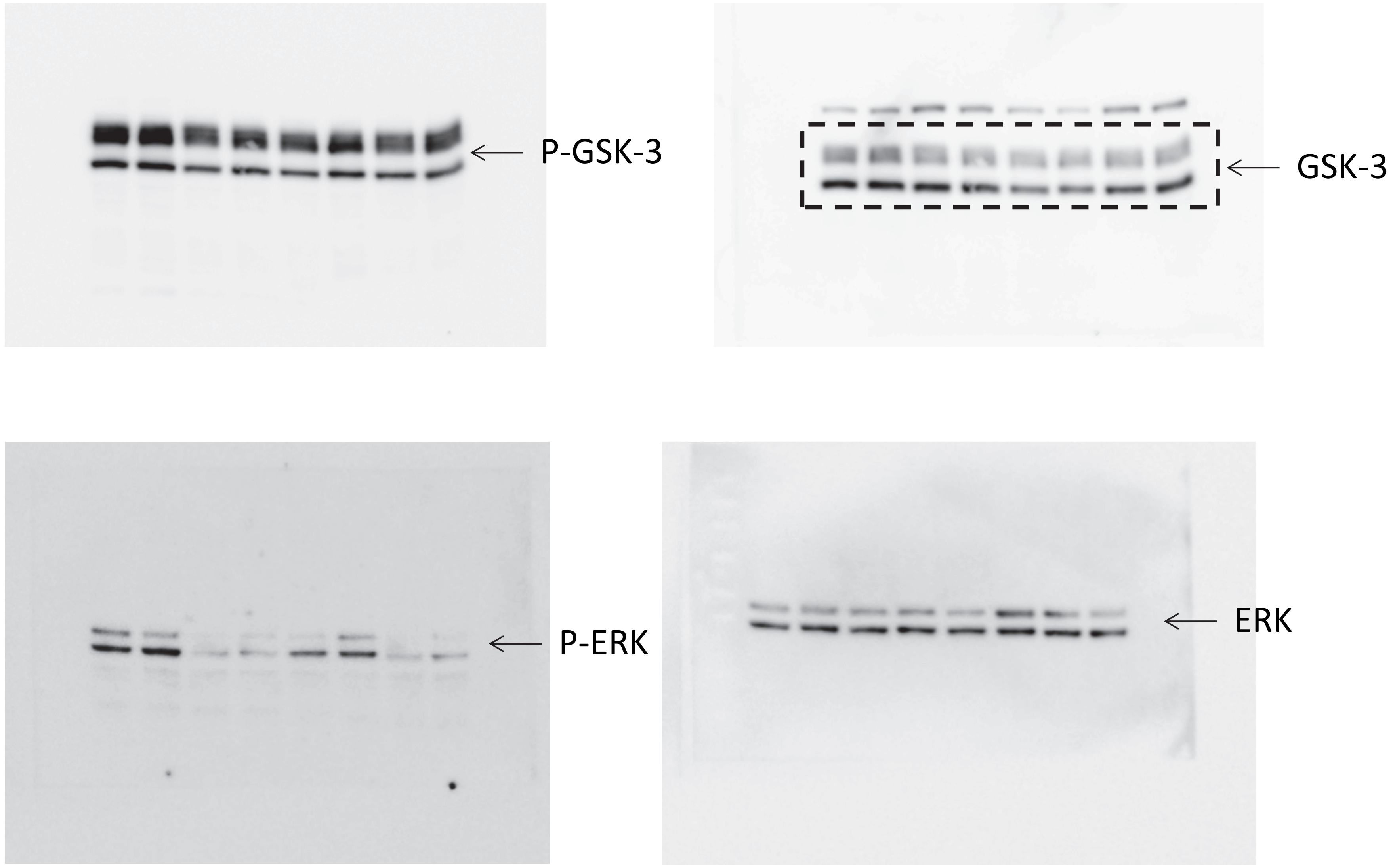
P-GSK-3
GSK-3
P-ERK
ERK

Full unedited gels for figure 2e | Liver

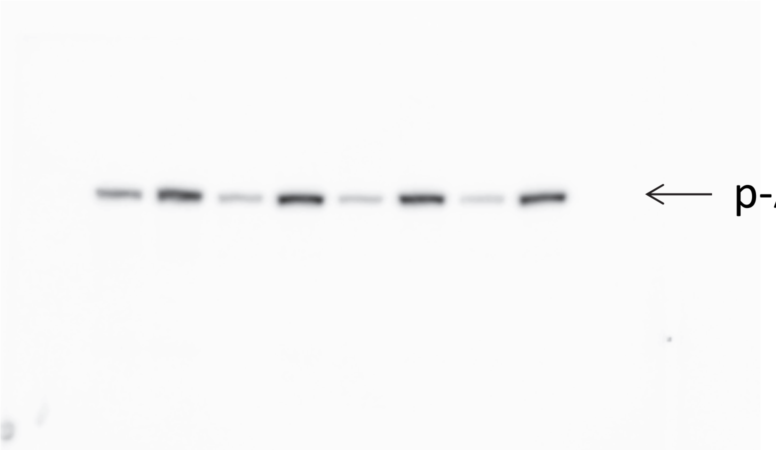

p-AKT

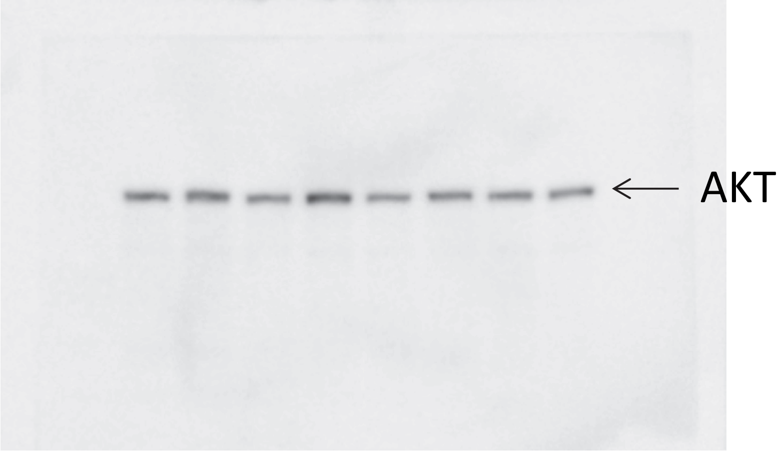

AKT

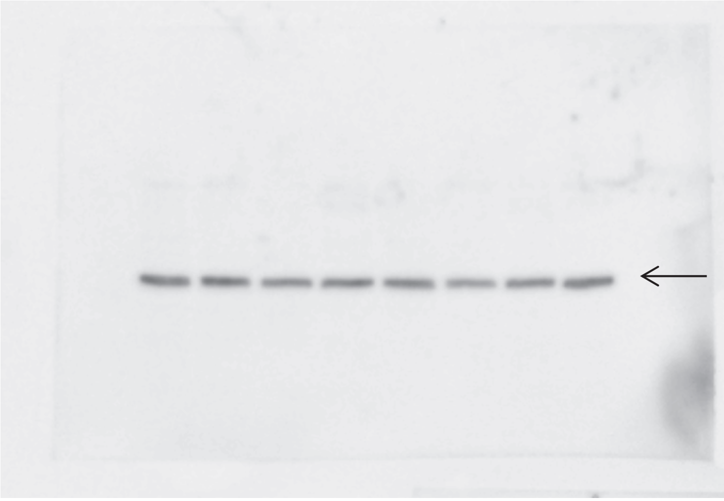

GAPDH

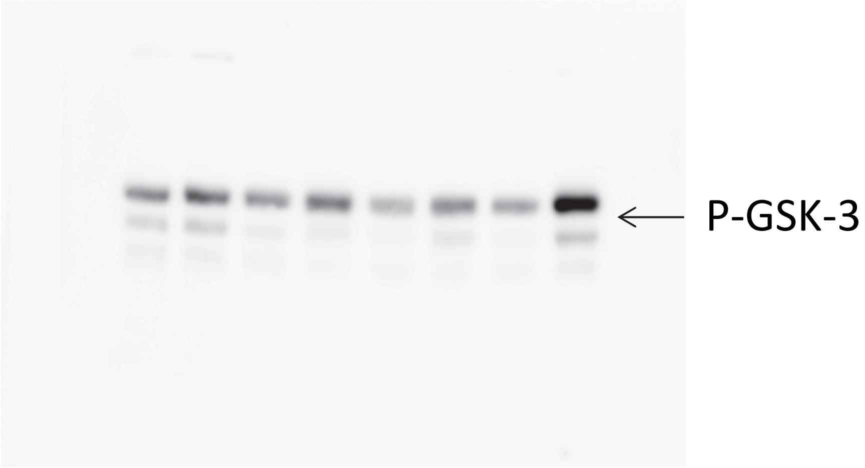
P-GSK-3

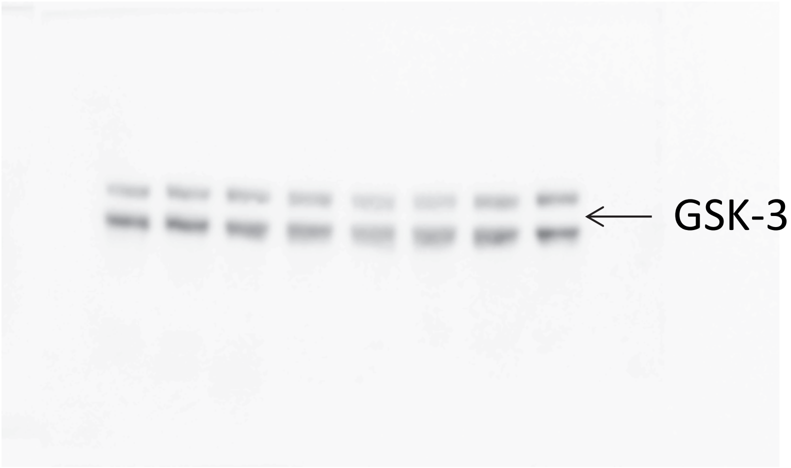
GSK-3

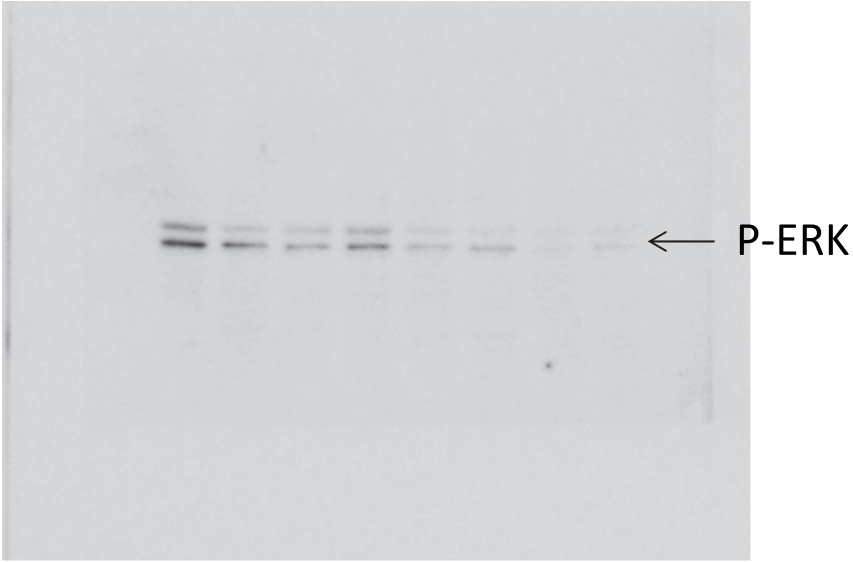
P-ERK

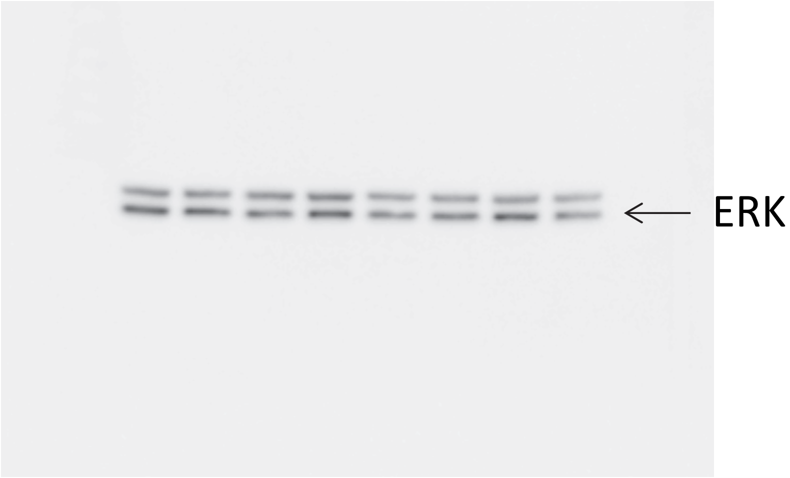
ERK

Full unedited gels for figure 2e | White adipose tissue

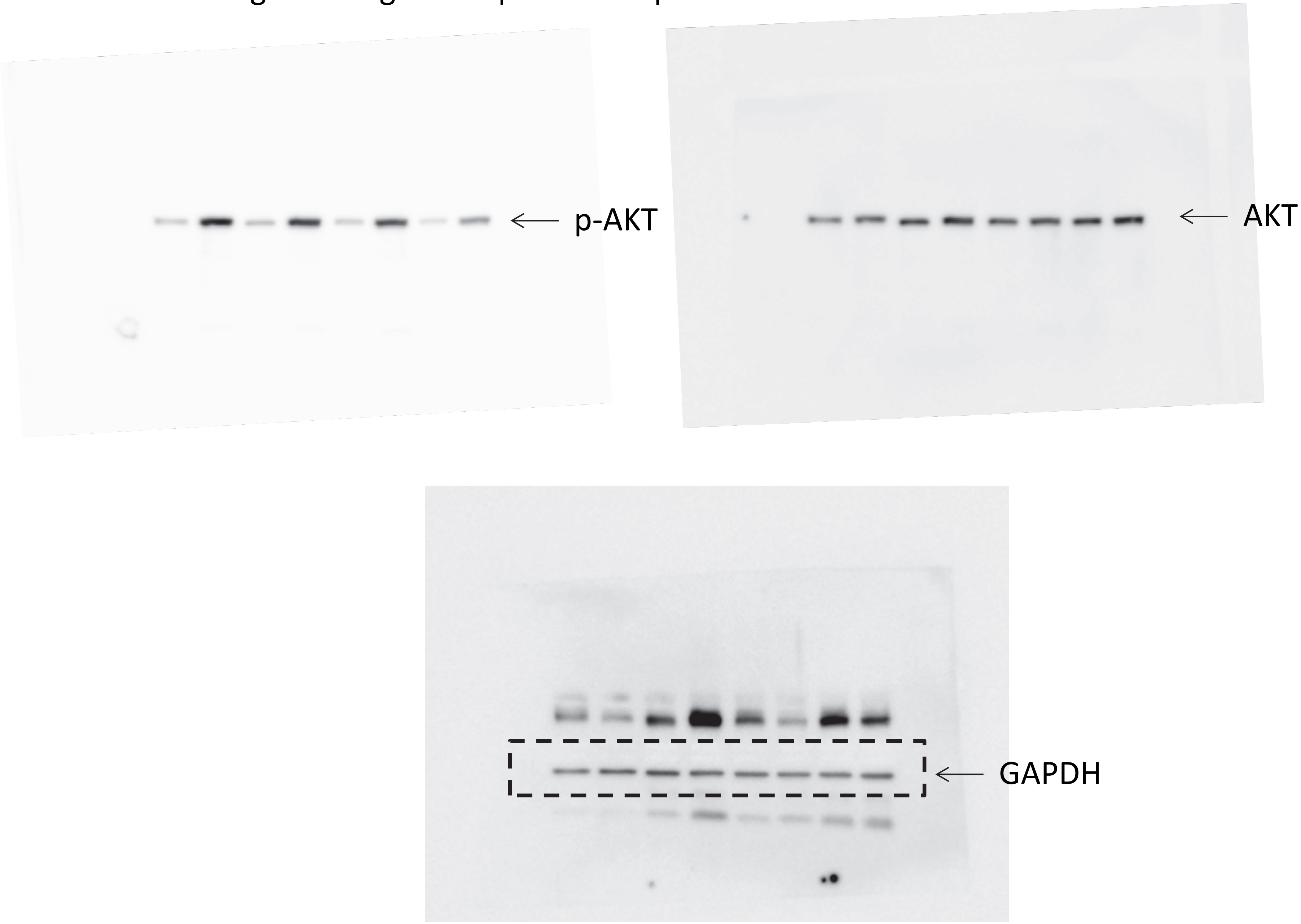

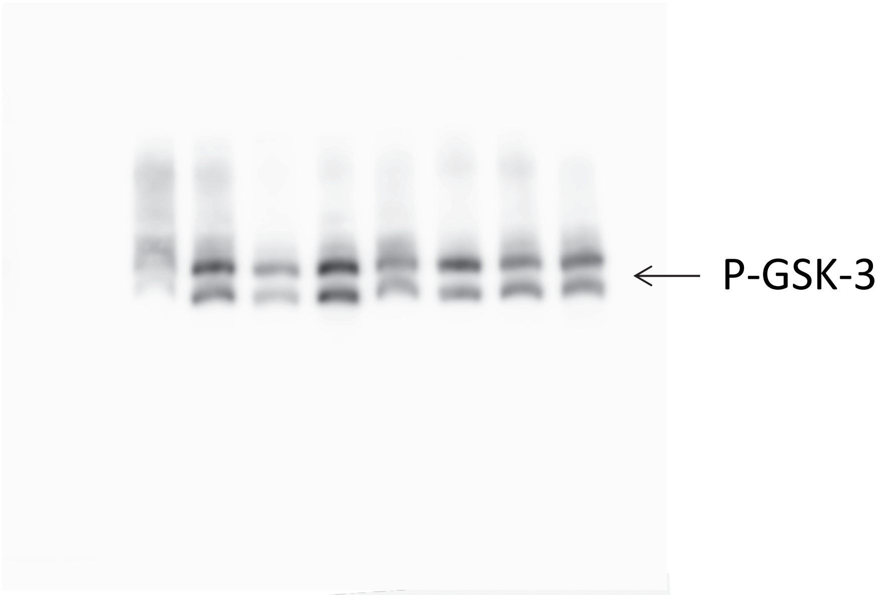
P-GSK-3

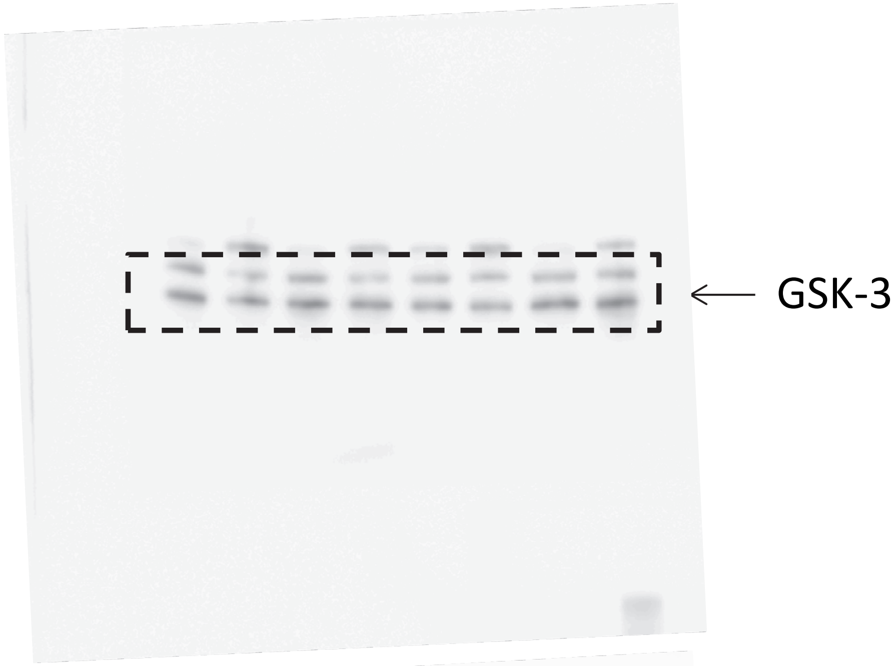
GSK-3

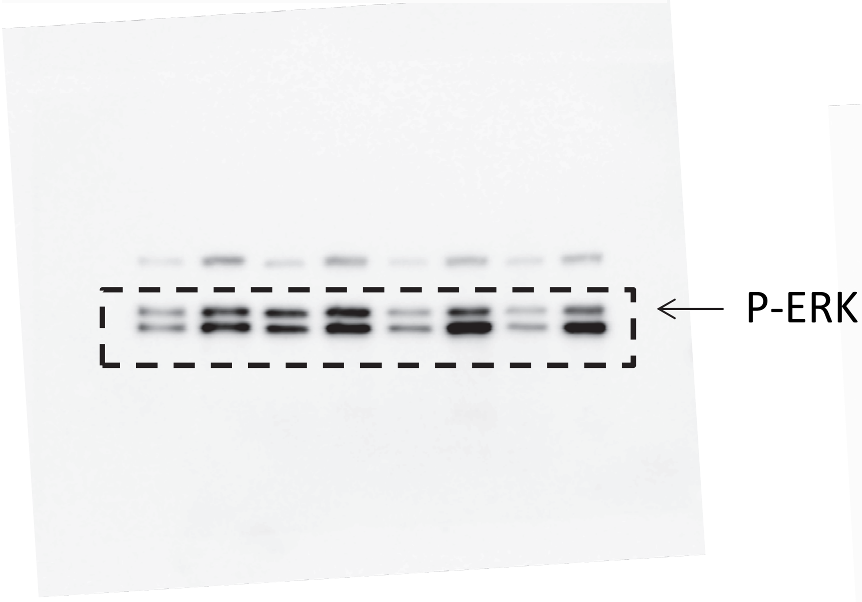
P-ERK

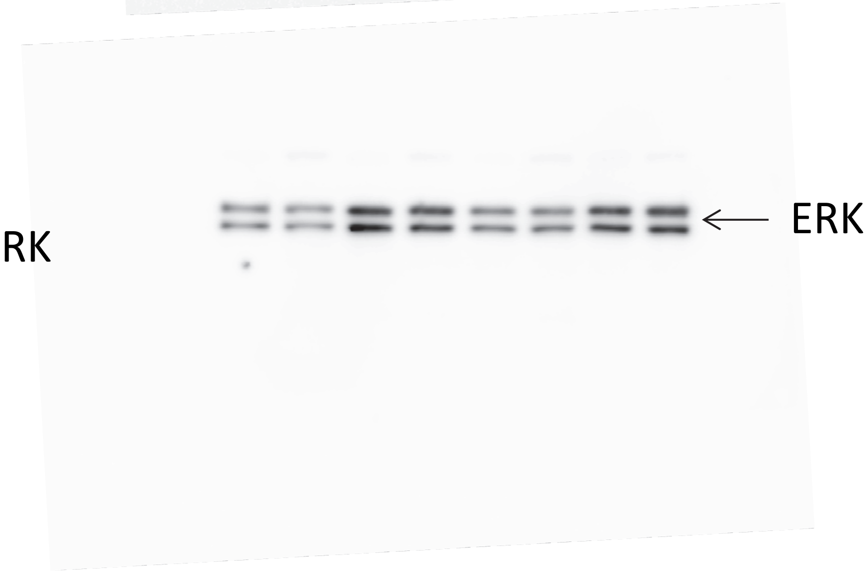
ERK

Full unedited gels for figure 3a | Liver

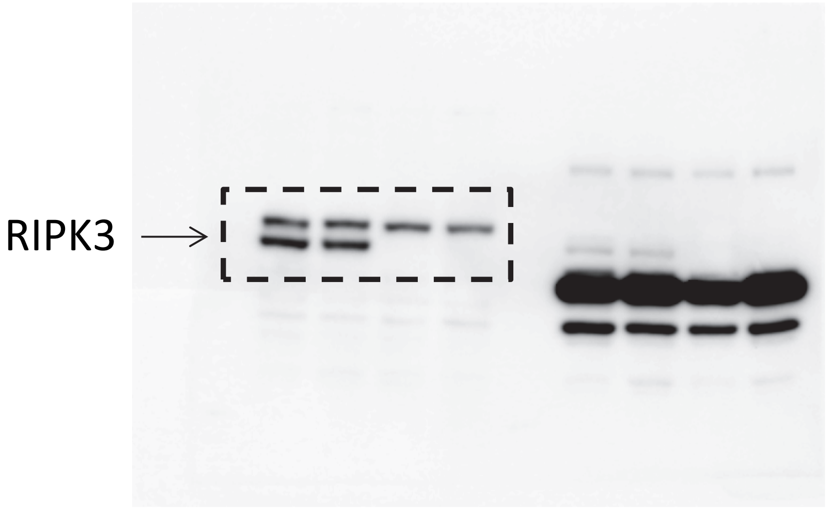

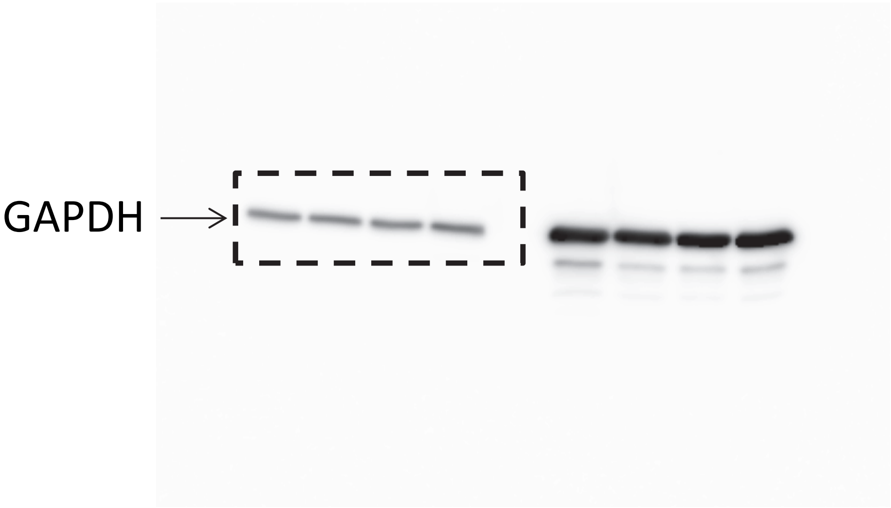

Full unedited gels for figure 3a | Skeletal muscle

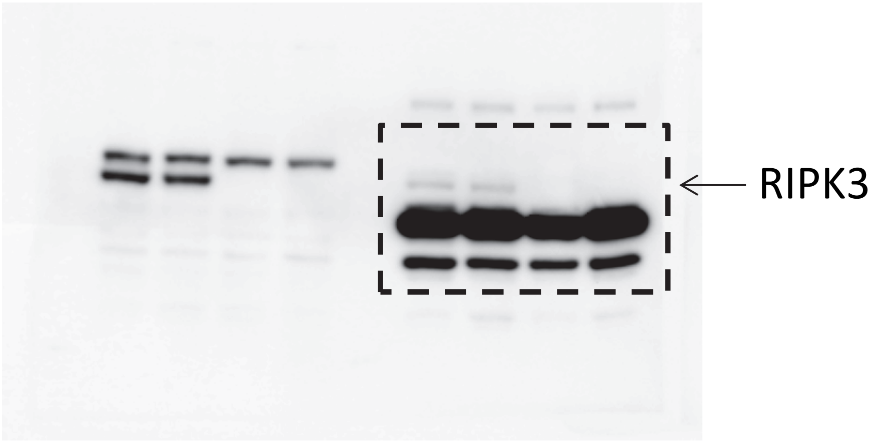

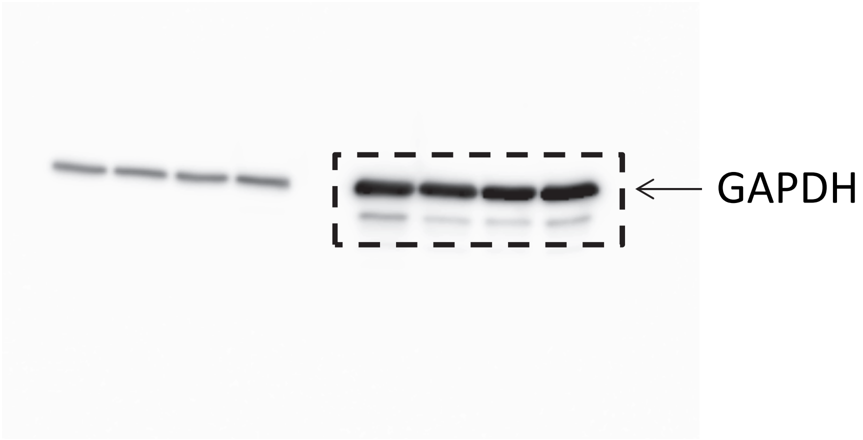

Full unedited gels for figure 3a | White adipose tissue

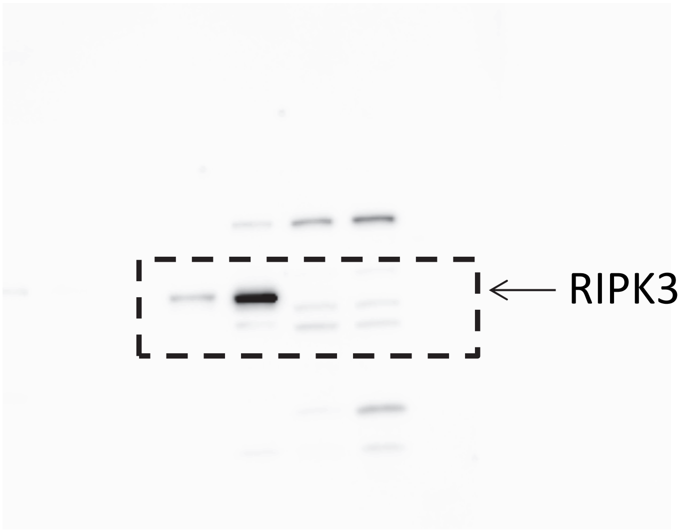

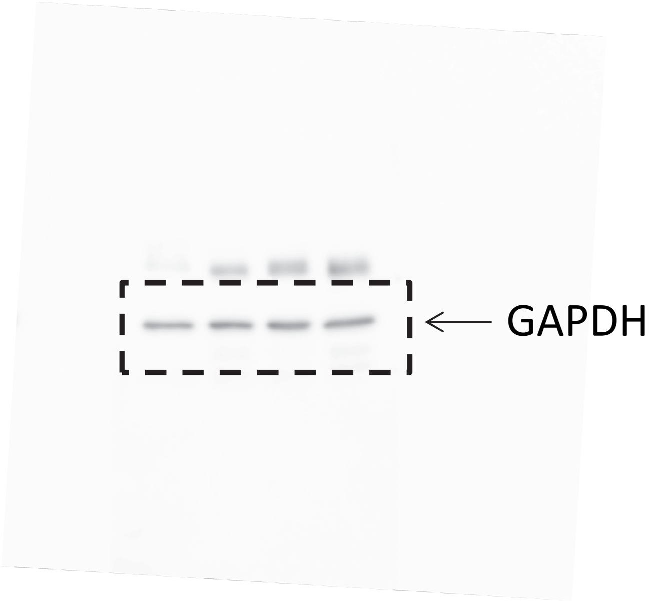

Full unedited gels for figure 3b | White adipose tissue

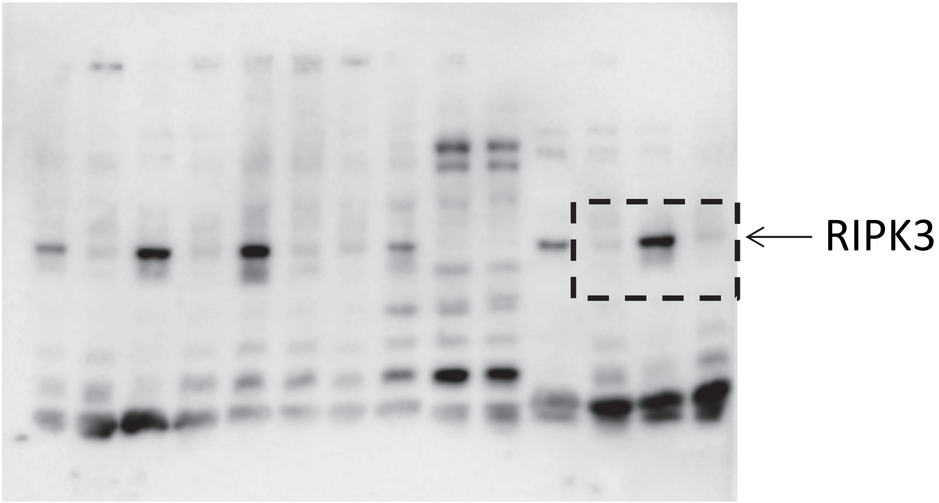

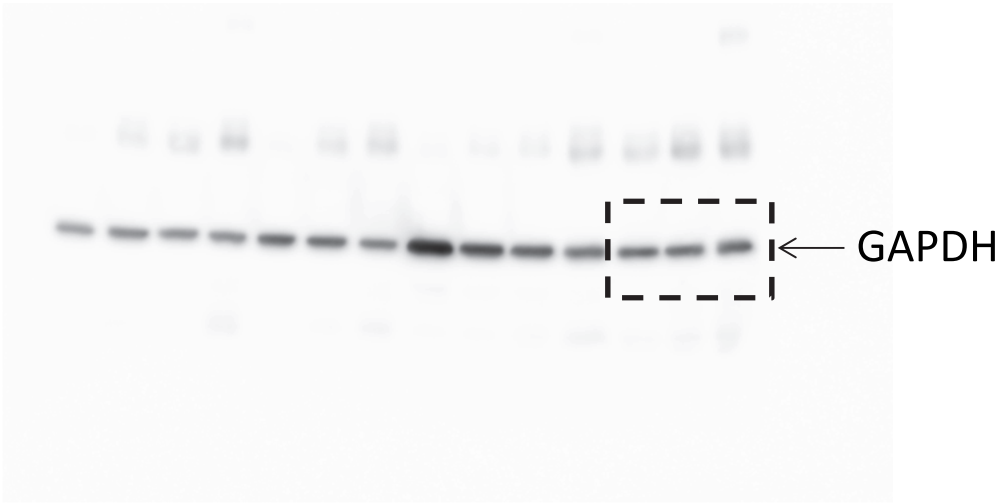

Full unedited gels for figure 3c | White adipose tissue / HFD

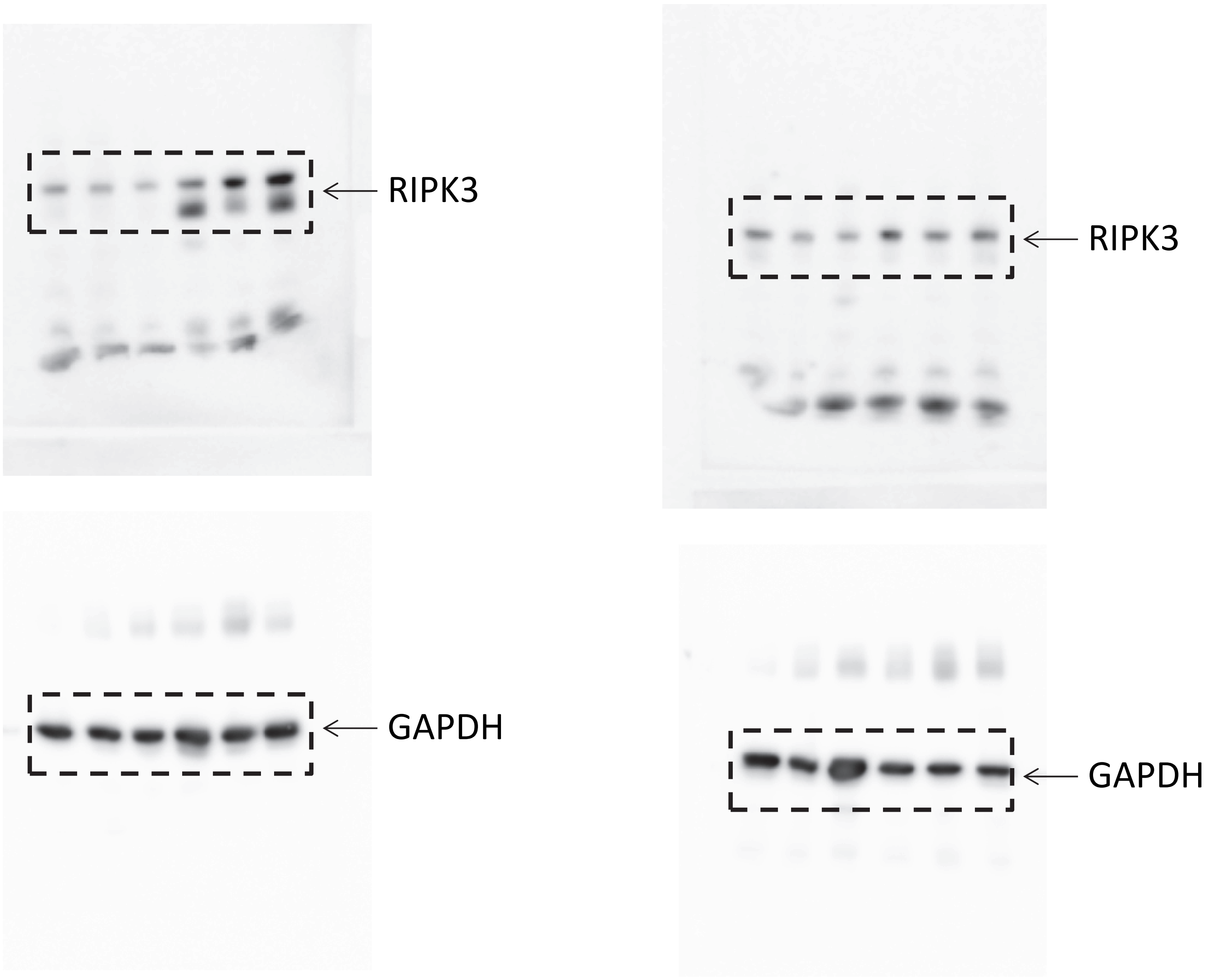

Full unedited gels for figure 5b | White adipose tissue

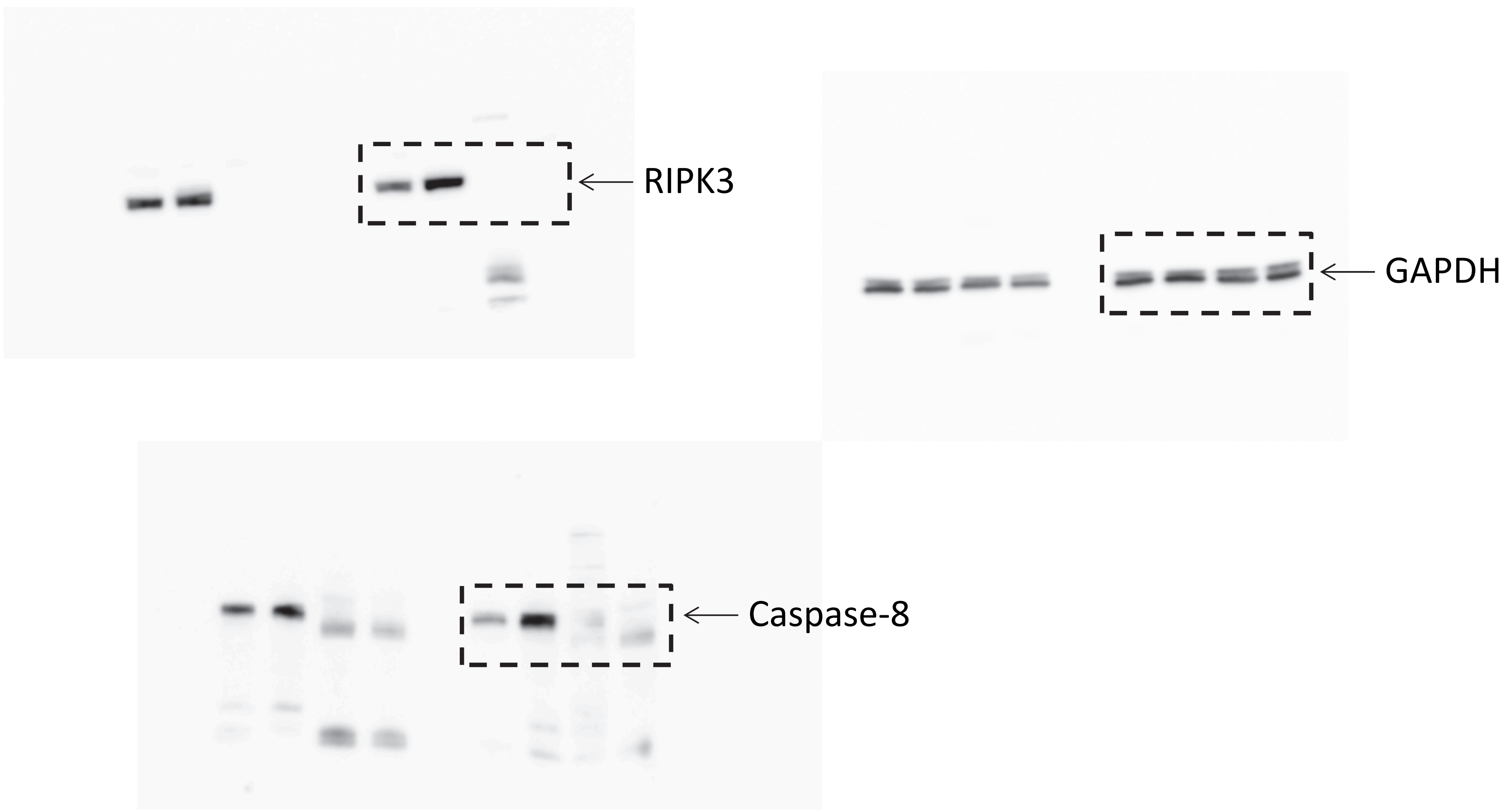

Full unedited gels for figure 6d | 3T3-L1

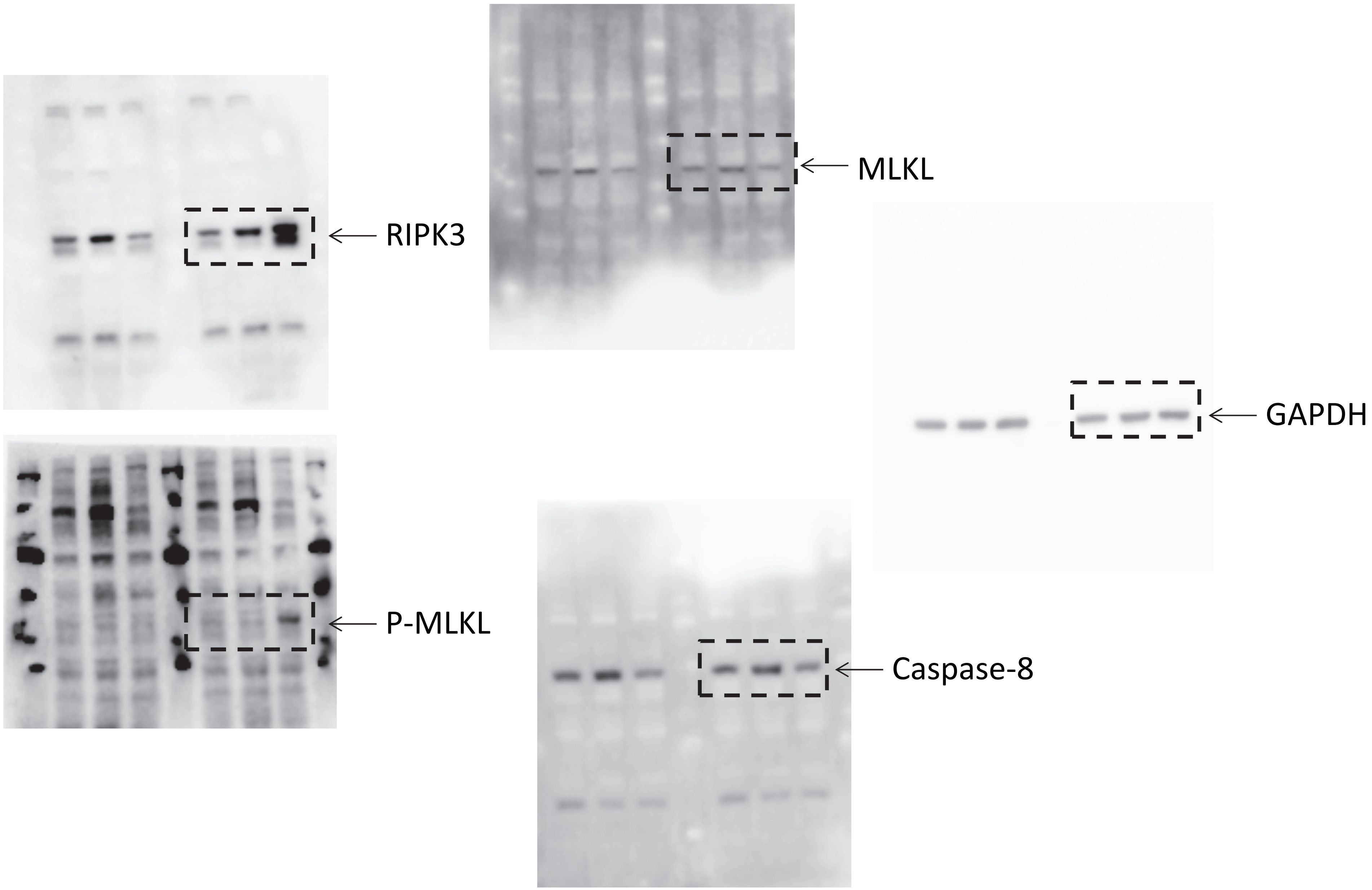

Full unedited gels for figure 6d | 3T3-L1

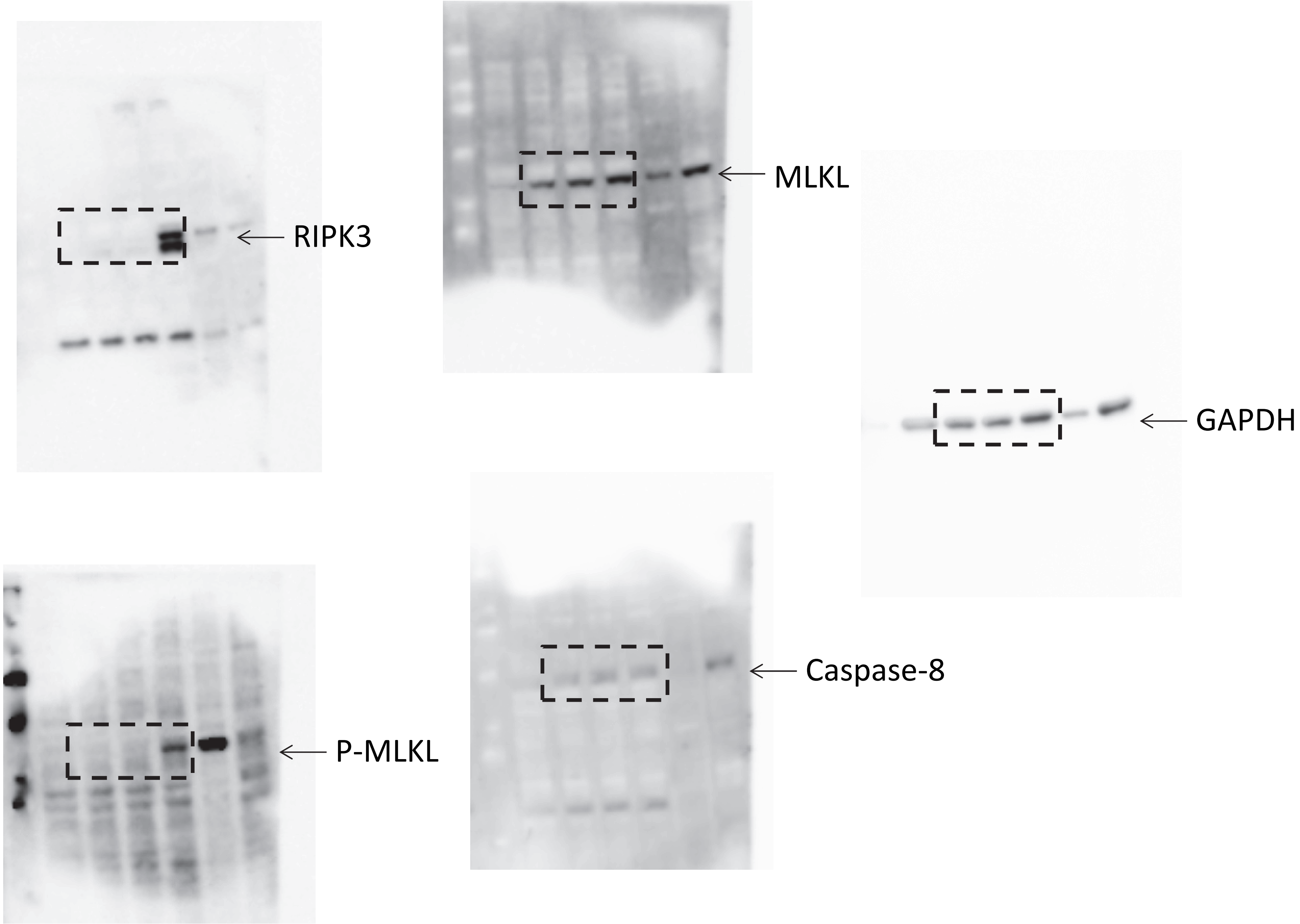

Full unedited gels for figure 6e | 3T3-L1

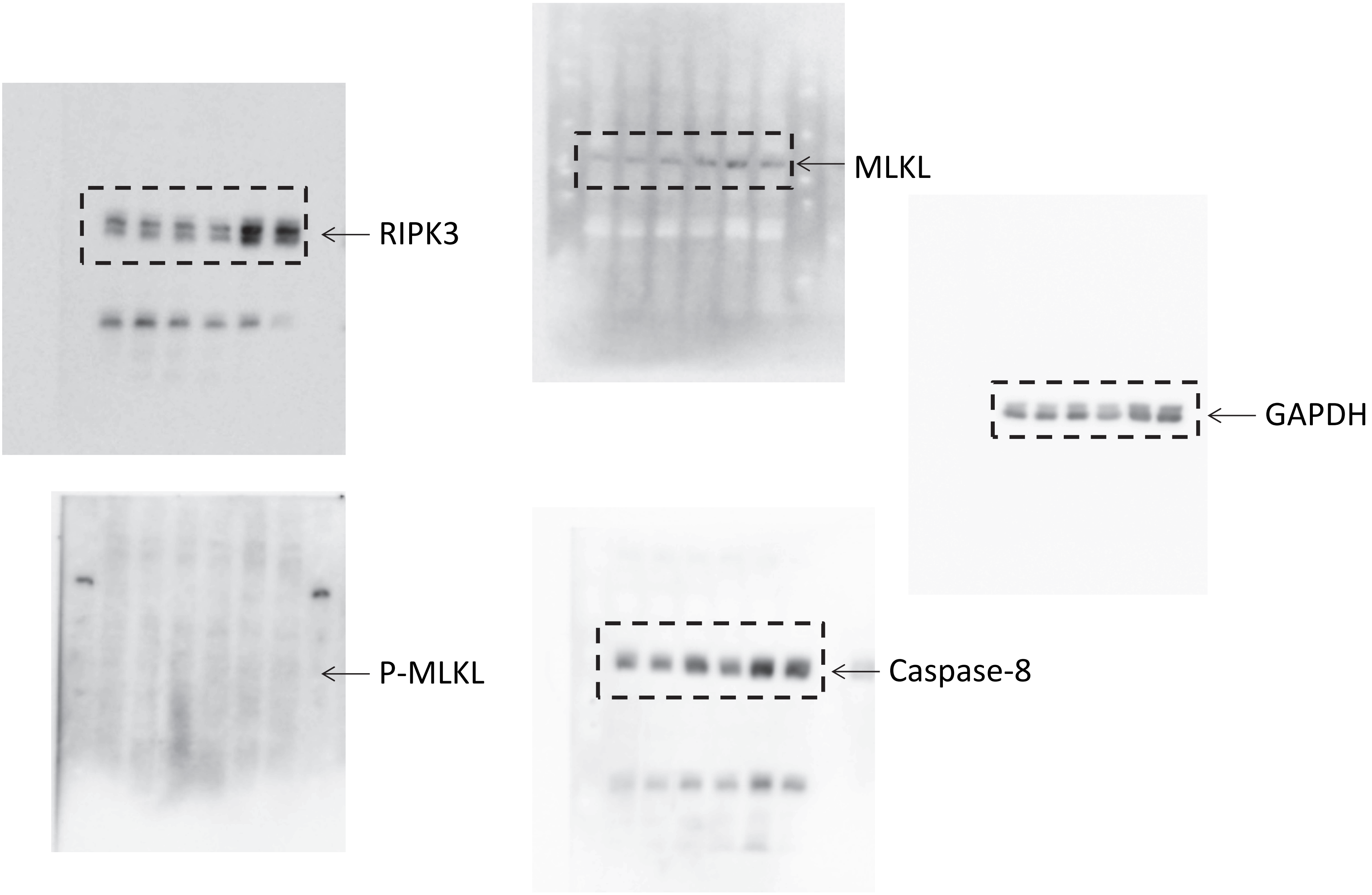

Full unedited gels for figure 7b | Macrophages

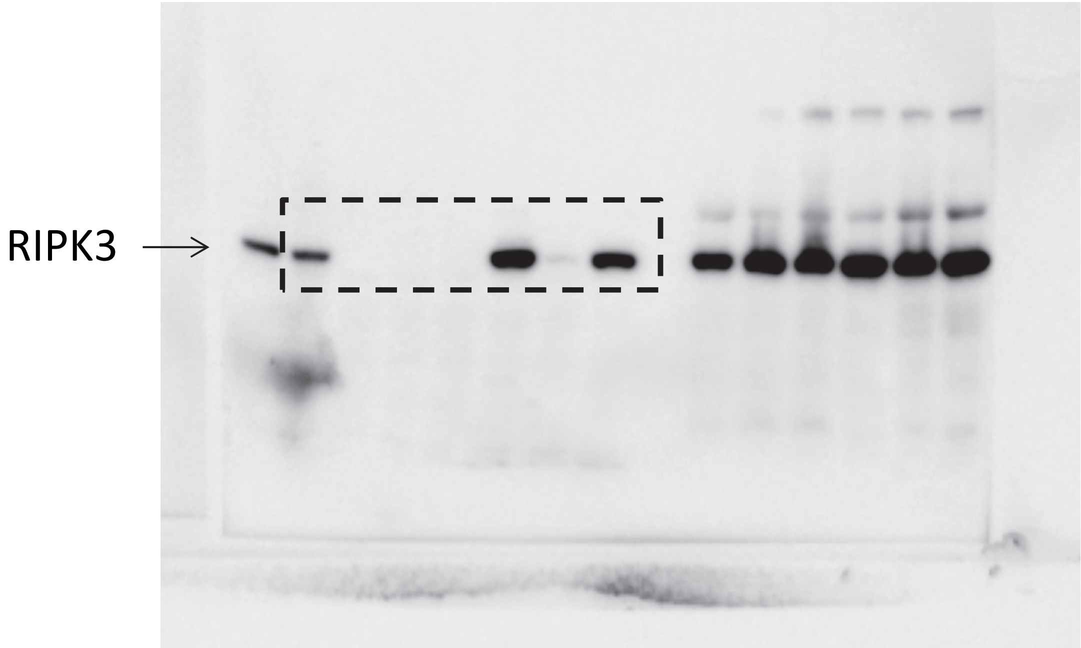

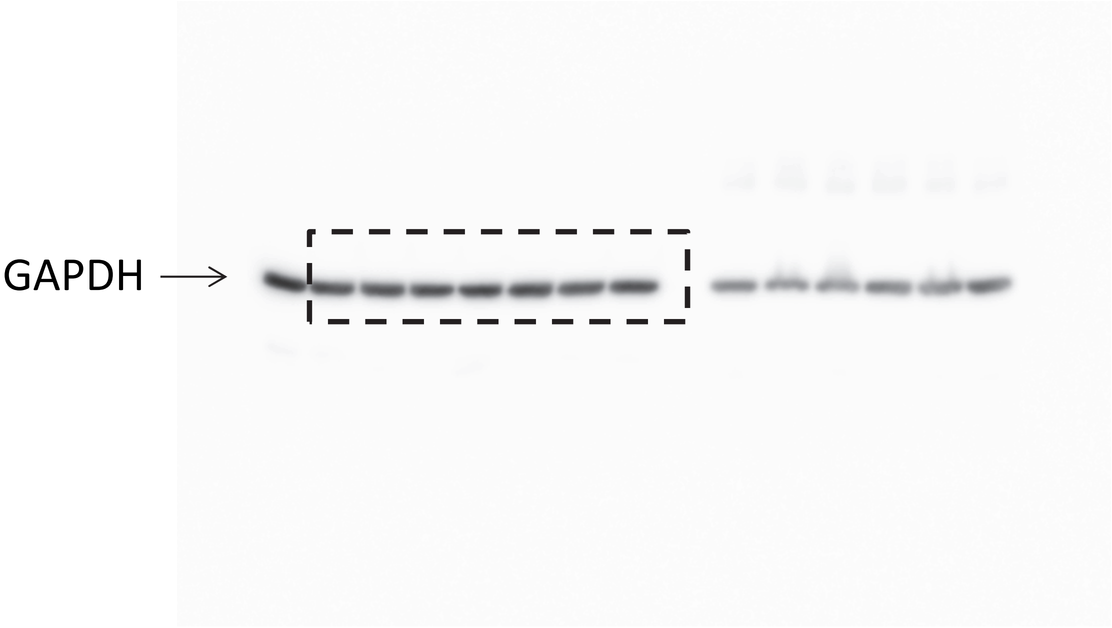

Full unedited gels for figure 7b | White adipose tissue

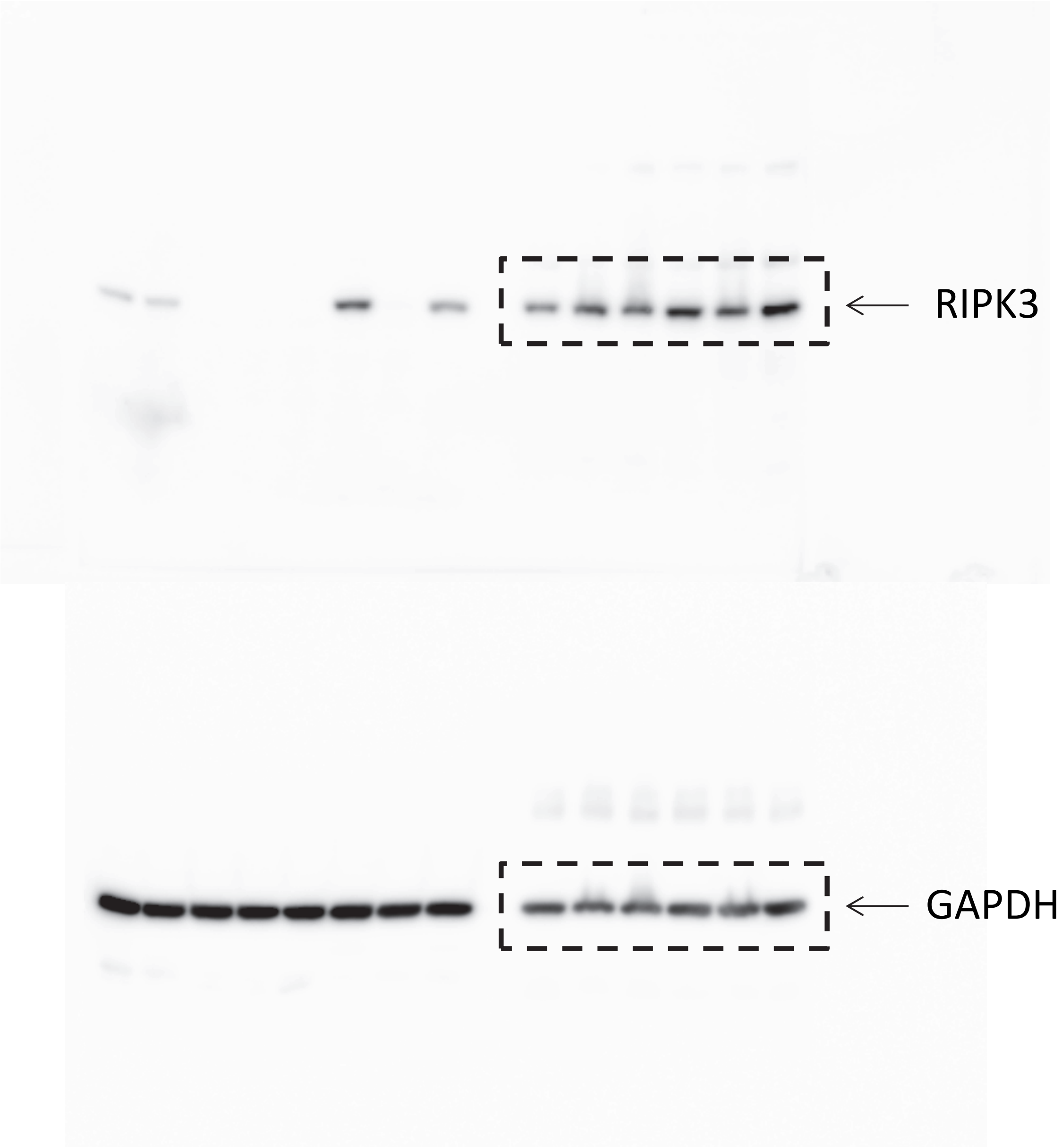

Full unedited gels for sup fig 5a | Visceral white adipose tissue Human

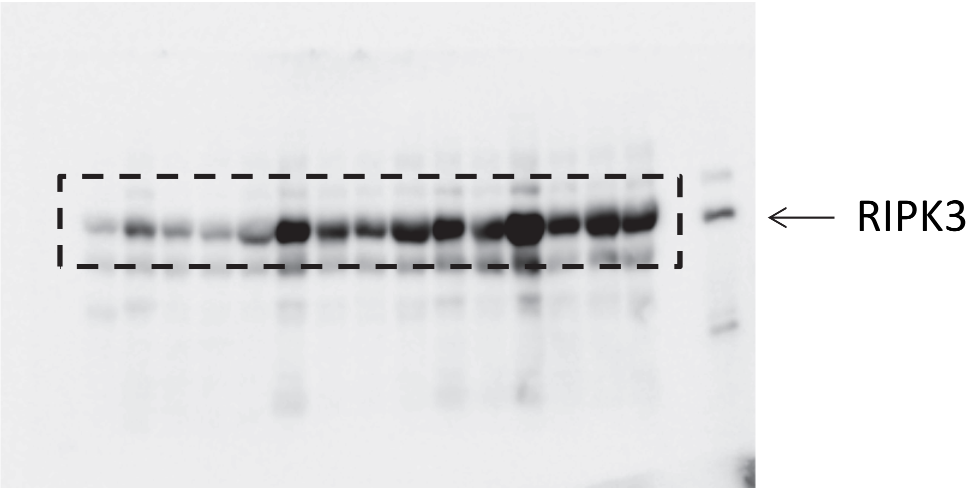

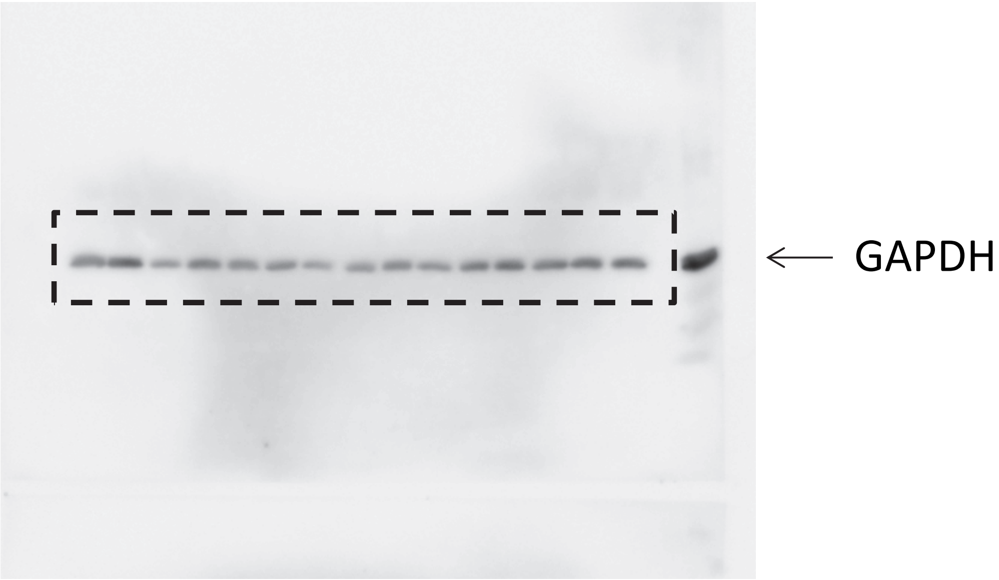

Full unedited gels for sup fig 5d | Visceral white adipose tissue Human

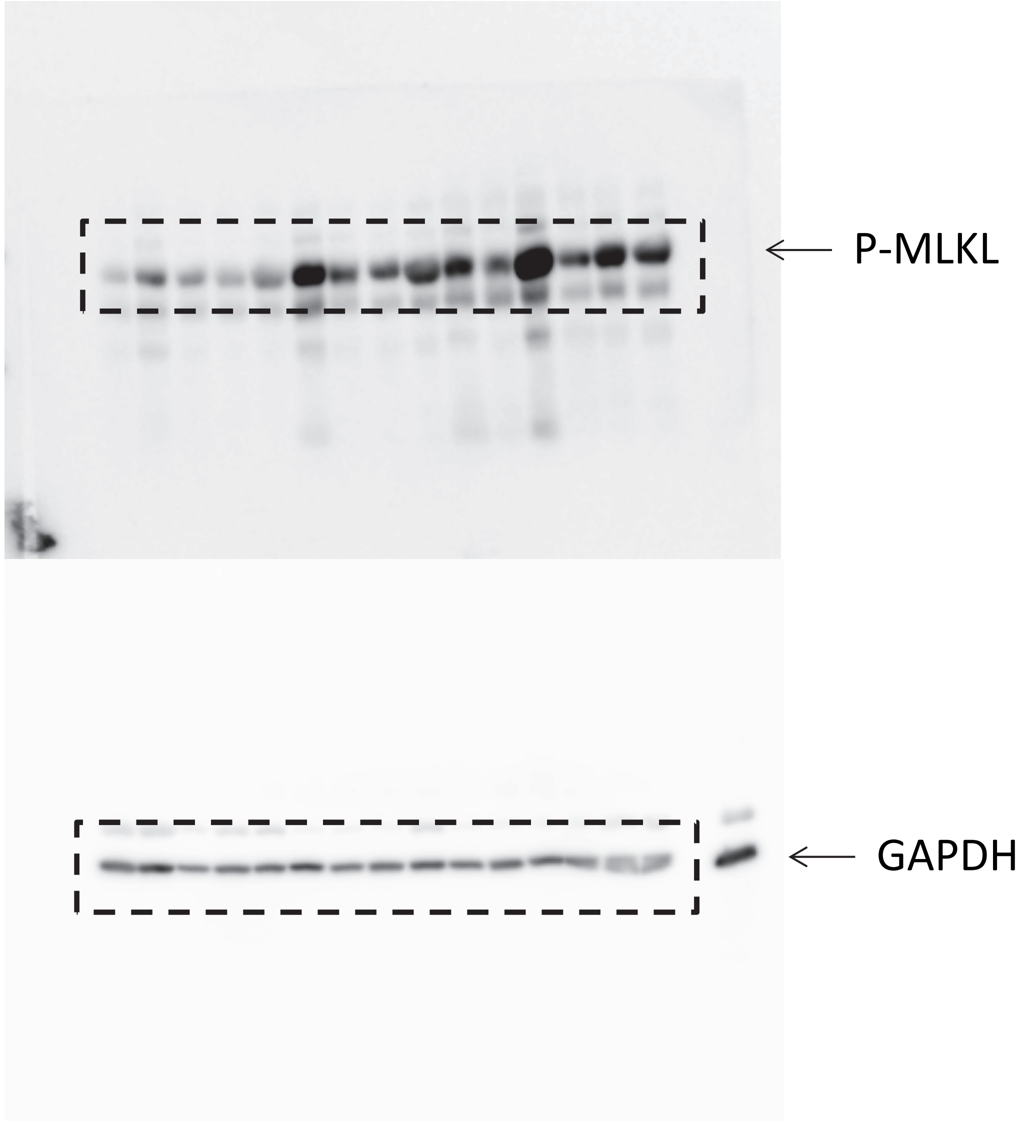

Full unedited gels for sup fig 6 | epiWAT Time Course

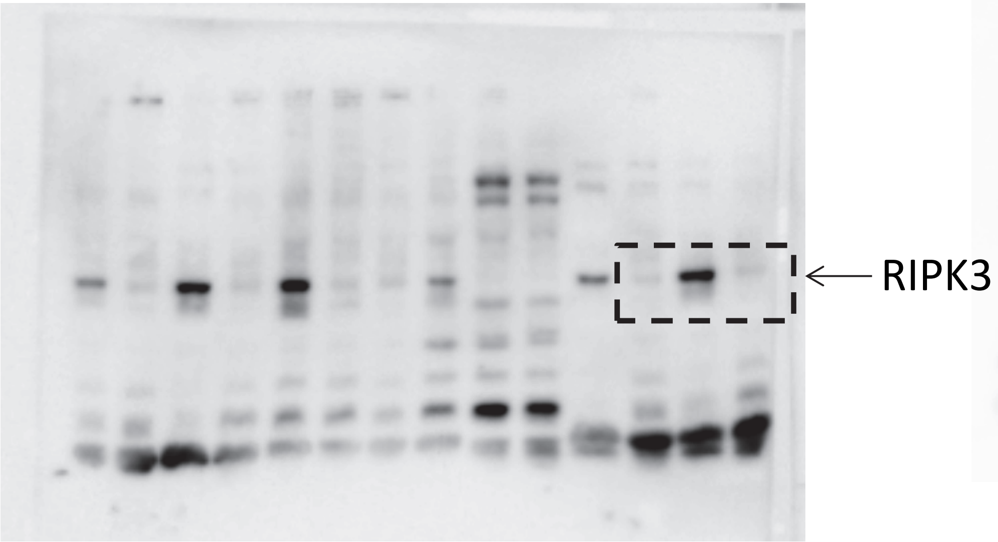

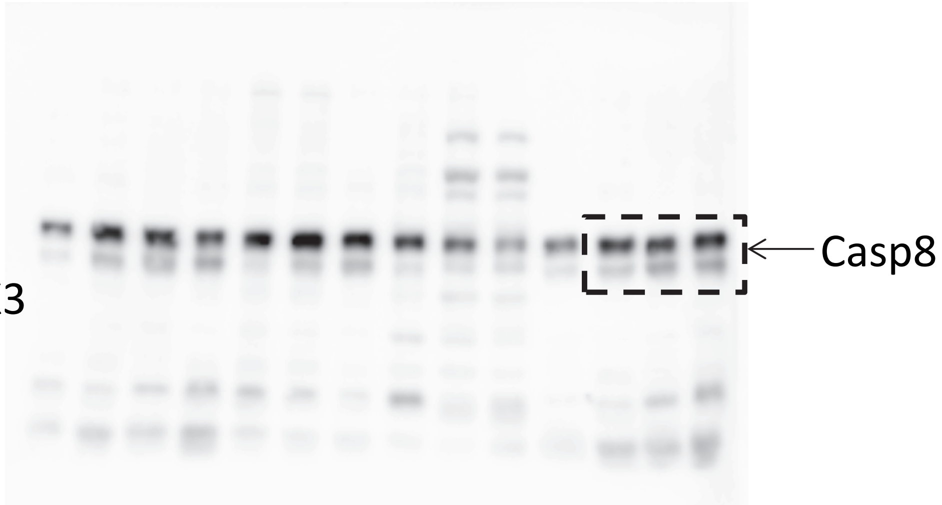

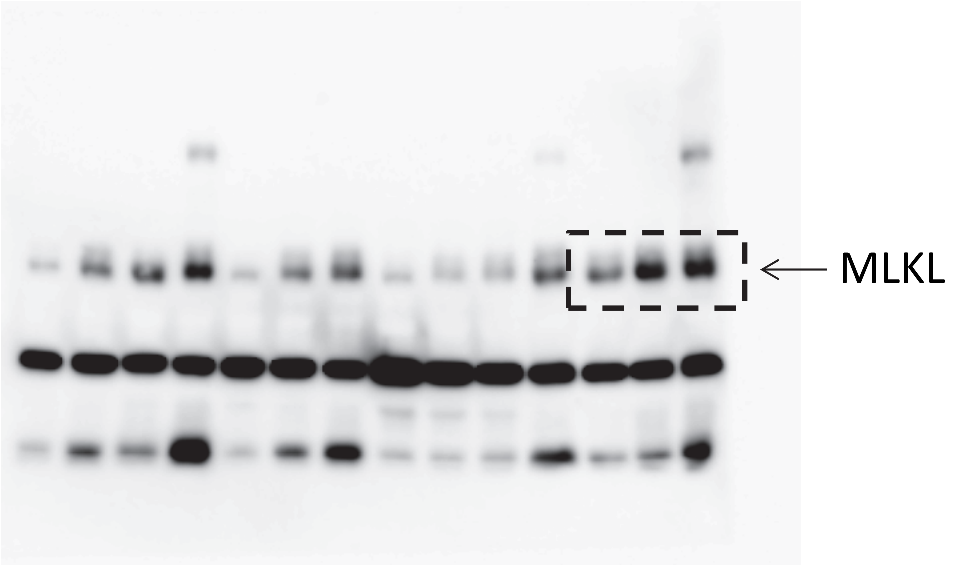

Full unedited gels for sup fig 6 | epiWAT Time Course

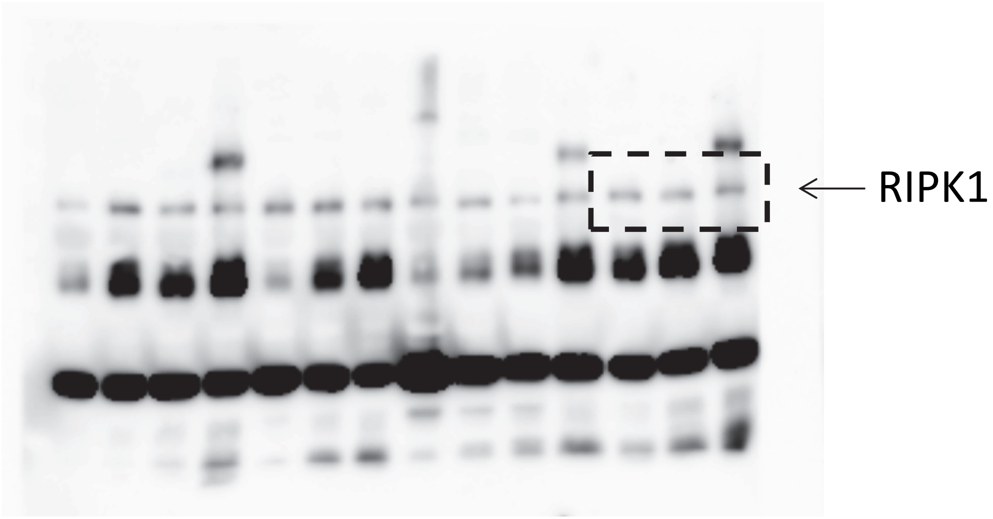

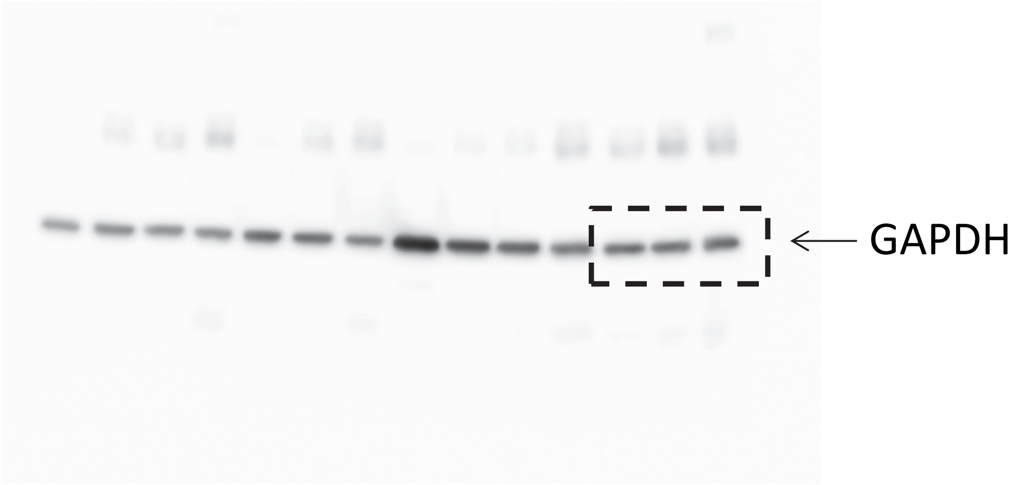

Supplementary Table 1. NAS score of mouse liver samples.

***$p<0.001$ for comparisons between lean and obese WT and RIPK3-KO groups.

WT and RIPK3-KO obese mice have a significant increase of NAS score compared to lean groups

	Fat	Ballooning	Inflammation	NAS	
	0	0	0	0	
WT	0	0	1	1	
NCD	0	0	1	1	
	0	0	1	1	
	2	1	3	6	
	2	1	1	4	
	1	0	1	2	
WT	1	0	1	2	
CD-HFD	2	2	2	6	***
	3	2	2	7	
	3	2	1	6	
	3	2	1	6	
	1	0	1	2	
	0	0	1	0	
RIPK3$^{-/-}$	0	0	0	1	
NCD	0	0	1	0	
	0	0	0	1	
	3	2	1	6	
	3	2	1	6	
	3	2	1	6	
RIPK3$^{-/-}$	3	2	1	6	
CD-HFD	3	2	1	6	***
	3	2	2	7	
	3	2	2	7	
	2	2	2	6	
	3	2	2	7	

Supplementary Table 2. Characteristics of study participants.

**p<0.01, ***p<0.001 for comparisons between lean and obese groups.
Data are given as mean±SD.

	Lean (n=10)	Obese (n=10) non-diabetic	Obese (n=10) diabetic
T2D	no	no	yes
Age (years)	65.2±9.5	52.1±16.5	54.1±9.9
Men/Women (*n*)	2/8	4/6	5/5
BMI (kg/m^2)	22.0±2.8	42.3±9.9***	45.9±12.3***
Body fat (%)	19.7±3.7	36.5±9.3***	42.3±7.3***
Fasting plasma glucose (mmol/L)	5.4±0.6	5.6±0.6	7.9±2.1***
Fasting plasma insulin (pmol/l)	22.1±27.6	58.3±35.9**	191±102**
Glucose infusion rate, clamp (µmol/kg/min)	101±13.3	73±26.5**	31±19.8***
HbA1c (%)	5.2±0.2	5.5±0.2	7.2±1.1**
Total cholesterol (mmol/l)	5.2±1.0	5.3±1.0	5.0±1.6
LDL-cholesterol (mmol/l)	3.1±1.4	2.9±0.7	3.0±1.1
HDL-cholesterol (mmol/l)	1.3±1.6	1.5±0.7	1.0±0.1**
Triglycerides (mmol/l)	1.1±0.3	0.9±0.3	2.1±1.2***
Free fatty acids (mmol/l)	0.2±0.2	0.4±0.3	1.2±0.3***
